# Flexible Tactile Sensing Systems: Challenges in Theoretical Research Transferring to Practical Applications

**DOI:** 10.1007/s40820-025-01872-4

**Published:** 2025-08-20

**Authors:** Zhiyu Yao, Wenjie Wu, Fengxian Gao, Min Gong, Liang Zhang, Dongrui Wang, Baochun Guo, Liqun Zhang, Xiang Lin

**Affiliations:** 1 Lab of Polymer Additive Manufacturing, School of Chemistry and Biological Engineering, University of Science and Technology Beijing, Beijing, 100083 People’s Republic of China; 2 Institute of Emergent Elastomers, Guangzhou International Campus, South China University of Technology, Guangzhou, 511442 People’s Republic of China; 3 Center of Advanced Elastomer Materials, State Key Laboratory of Organic-Inorganic Composites, Beijing University of Chemical Technology, Beijing, 100029 People’s Republic of China; 4 School of Chemical Engineering and Technology, Xi’an Jiaotong University, Xi’an, 710049 People’s Republic of China

**Keywords:** Tactile sensation, Flexibility, Multimodal, System integration, Robotic haptics

## Abstract

Since the first design of tactile sensors was proposed by Harmon in 1982, tactile sensors have evolved through four key phases: industrial applications (1980s, basic pressure detection), miniaturization via MEMS (1990s), flexible electronics (2010s, stretchable materials), and intelligent systems (2020s-present, AI-driven multimodal sensing). With the innovation of material, processing techniques, and multimodal fusion of stimuli, the application of tactile sensors has been continuously expanding to a diversity of areas, including but not limited to medical care, aerospace, sports and intelligent robots. Currently, researchers are dedicated to develop tactile sensors with emerging mechanisms and structures, pursuing high-sensitivity, high-resolution, and multimodal characteristics and further constructing tactile systems which imitate and approach the performance of human organs. However, challenges in the combination between the theoretical research and the practical applications are still significant. There is a lack of comprehensive understanding in the state of the art of such knowledge transferring from academic work to technical products. Scaled-up production of laboratory materials faces fatal challenges like high costs, small scale, and inconsistent quality. Ambient factors, such as temperature, humidity, and electromagnetic interference, also impair signal reliability. Moreover, tactile sensors must operate across a wide pressure range (0.1 kPa to several or even dozens of MPa) to meet diverse application needs. Meanwhile, the existing algorithms, data models and sensing systems commonly reveal insufficient precision as well as undesired robustness in data processing, and there is a realistic gap between the designed and the demanded system response speed. In this review, oriented by the design requirements of intelligent tactile sensing systems, we summarize the common sensing mechanisms, inspired structures, key performance, and optimizing strategies, followed by a brief overview of the recent advances in the perspectives of system integration and algorithm implementation, and the possible roadmap of future development of tactile sensors, providing a forward-looking as well as critical discussions in the future industrial applications of flexible tactile sensors.

## Highlights

- This review presents current advances in flexible tactile sensor research from multifaceted perspectives including mechanisms, materials, structural design, and system integration.
- It establishes performance-oriented rational design principles for sensors in practical.
- It summarized the challenges and strategies in translating flexible tactile sensing systems into practical applications, and proposed a research roadmap for future investigations.

## Graphical abstract

**Figure:**
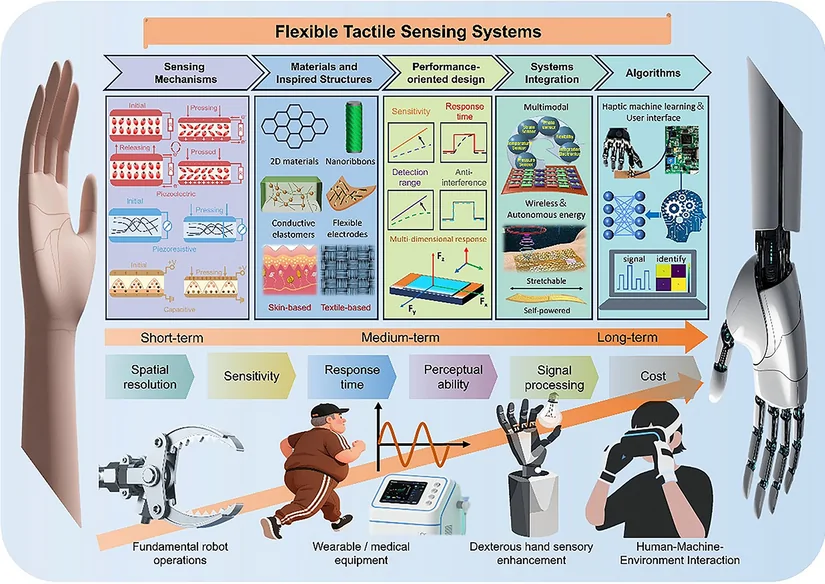

## Introduction

As an important sensory organ of the human body, skin is of great significance for object recognition, motion control, and the realization of social activities. Bioreceptors with various functional characteristics are distributed throughout the human tactile system to respond to different external stimuli (including but not limited to pressure, temperature, stretching, and compression) and thereby provide recognition and feedback. The specific process is that mechanical receptors on the skin receive external stimuli and convert them into neural impulses [1–4]. The main functions of adaptation, filtering, amplification, and memory between neurons and synapses process neural signals, which are then transmitted to the cerebral cortex to achieve advanced functions such as classification, recognition, and learning [5–8]. Inspired by the various perceptual abilities of the human skin, tactile sensors that convert applied stimuli into electronic signals to perceive and quantify mechanical stimuli have received widespread attentions. So far, tactile sensors mainly integrate four essential components: (i) stimuli-responsive sensing elements, (ii) electromechanical transduction elements, (iii) signal conditioning circuits for noise suppression and linearization, and (iv) integrated auxiliary modules (e.g., energy harvesting, wireless interfaces). With the integration of multifunctional sensing devices, the concept of tactile sensing system has been proposed, and various new types of touch screens and robotic arms with tactile sensing functions have been widely studied [9–12]. However, the essential properties, such as high sensitivity, fast response time, wide response range, and precise perception ability of human tactile organs, cannot be achieved by artificial tactile sensing systems.

In recent years, the emerging flexible electronic devices have made significant progress with the deepening of research in the field of nanomaterials, gradually replacing traditional rigid sensors and becoming an important research direction in the field of tactile sensing. The commonly used mechanisms for implementing tactile sensing include piezoresistive [13, 14], piezoelectric [15, 16], capacitive [17, 18], triboelectric [19–21], optoelectronic [22], and magnetoelectric inductions [23], which have been widely studied and continuously improved. For example, Yang et al. demonstrated a novel three-dimensional (3D) microconformal graphene electrode for ultra-sensitive and adjustable flexible capacitive pressure sensors, which enhances tactile sensing performance by increasing electrode roughness (a response speed of 30 ms and a detection limit of 1 mg) [24]. Based on the piezoelectric tactile sensing mechanism, Huang et al. proposed a method for preparing a rigid-in-soft structured tactile sensor array. The resulting tactile sensor array achieved relatively simple signal acquisition [25]. Inspired by the principles of bionics, Wang et al. developed a smart finger that goes beyond human tactile perception [26]. A sensor array composed of several typical materials with different frictional electrical signals was integrated into the smart finger to identify material types and roughness characteristics (recognition accuracy reaches 96.8%). In addition, researchers are still making numerous efforts to improve anti-interference ability and multidimensional sensing performance, including employing device functional layer based on bionics principles to achieve this goal.

Tactile sensing systems face core technical challenges in sensitive force perception, primarily centered around balancing precision, robustness, integration, and practicality. These mainly include inherent conflicts between sensitivity and measurement range, bottlenecks in resolution versus integration density as well as technical difficulties, such as dynamic response hysteresis and poor long-term stability. Besides, tactile is a multidimensional sensory system that contains various information, including but not limited to temperature [27, 28], humidity [29], and proximity [30]. Therefore, the integration of tactile sensors with various functional components has become a major focus of researchers. A multisensory integrated system can effectively promote the efficiency of human–computer interaction [31, 32]. Meanwhile, the coupling of multiple signals in the tactile sensing process greatly increases the complexity of the output signal. On the one hand, such progress has led to tactile signals becoming more precise and dense. On the other hand, this enhancement has also given rise to new challenges in the processing of tactile signals, including issues related to processing speed, signal decoupling, and information extraction [33–35]. Machine learning has achieved efficient perception and understanding in fields of computer vision and speech recognition. Some researchers have demonstrated tactile feedback systems and control interfaces based on machine learning [36–38]. Therefore, researchers hope to use this technology to gain a deeper understanding of tactile data and achieve further practical applications.

**Fig. 1:**
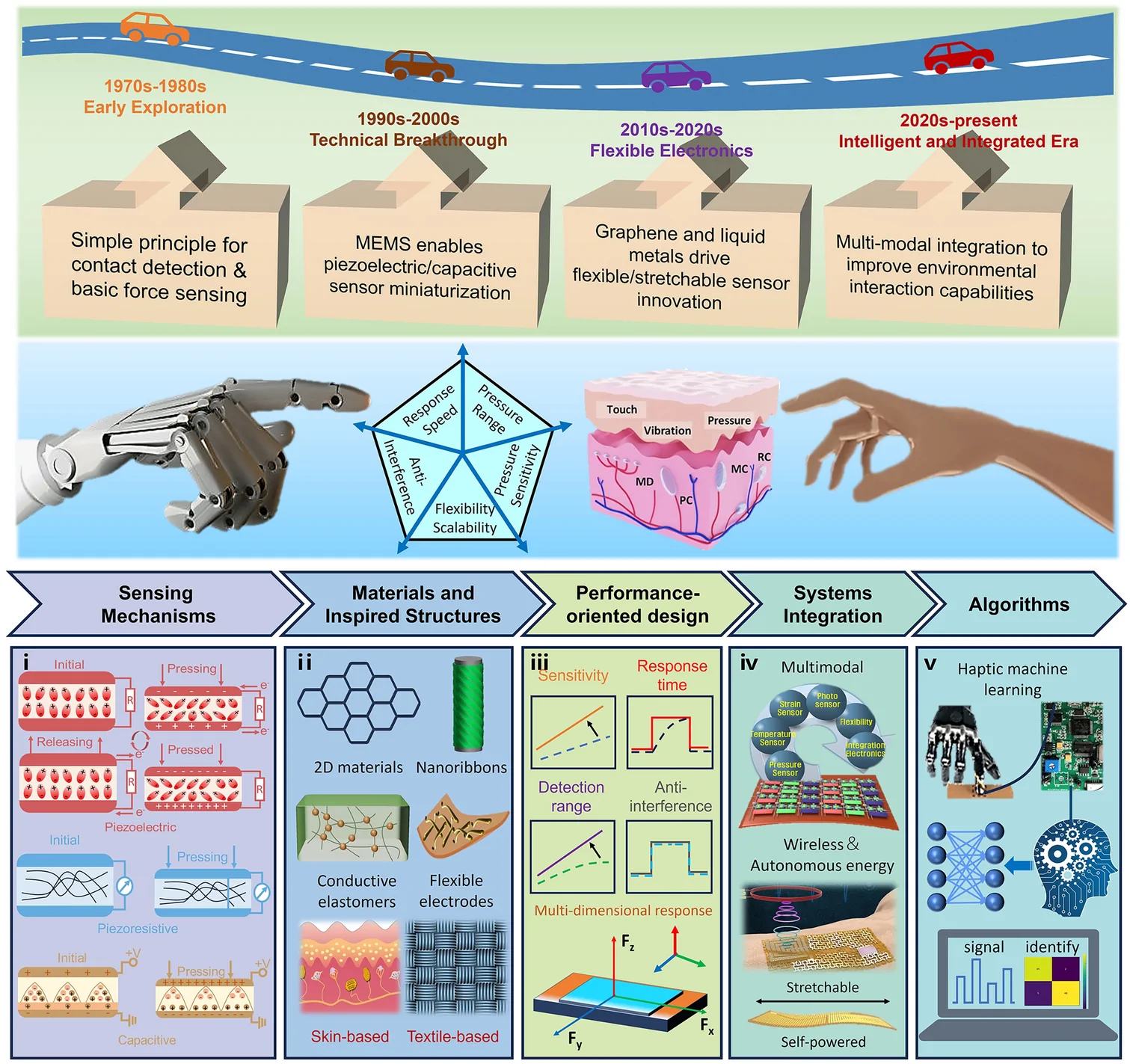
Flow structure of this review. i) Materials for tactile sensor components and two inspired structures for sensing implementation. ii) Common working mechanisms of tactile sensors. iii) Important performance parameters of tactile sensing. iv) System integration of tactile sensors. v) Machine learning tools and algorithms optimization for robot tactile perception

While numerous review articles have extensively discussed tactile sensing research, they predominantly focus on individual sensing mechanisms or isolated technological advancements, thereby constraining researchers' holistic understanding of integrated tactile system design. This review focuses on the updated development of tactile sensing systems, synthesizing cutting-edge research advancements to establish a conceptual blueprint for next-generation intelligent tactile perception architectures and taking a forward step in understanding their future applications. Figure 1 outlines an overview of the development framework for advanced tactile sensing systems. Through systematic analysis of operational mechanisms, material innovations with bioinspired structural designs, performance evaluation metrics, system-level integration strategies, and computational modeling algorithms, we methodically examine the systematic construction process of tactile sensing systems, providing foundational knowledge and forward-looking perspectives to inform future research trajectories in intelligent tactile perception technologies.

## Sensing Mechanisms

Mechanical arms are the earliest industrial robots and also the earliest modern robots. Since 1983, researchers have been committed to advancing the research process of advanced robotic arms and developing them toward flexibility and high precision [39, 40]. This has attracted much attention in the research of sensors for robotic arms. Furthermore, flexible tactile sensors are also used in other fields, including electronic skin, health monitoring devices and smart massage devices for medical service, smart wristbands in the consumer sector and metaverse gloves, and brain–computer interface interactions.

**Fig. 2:**
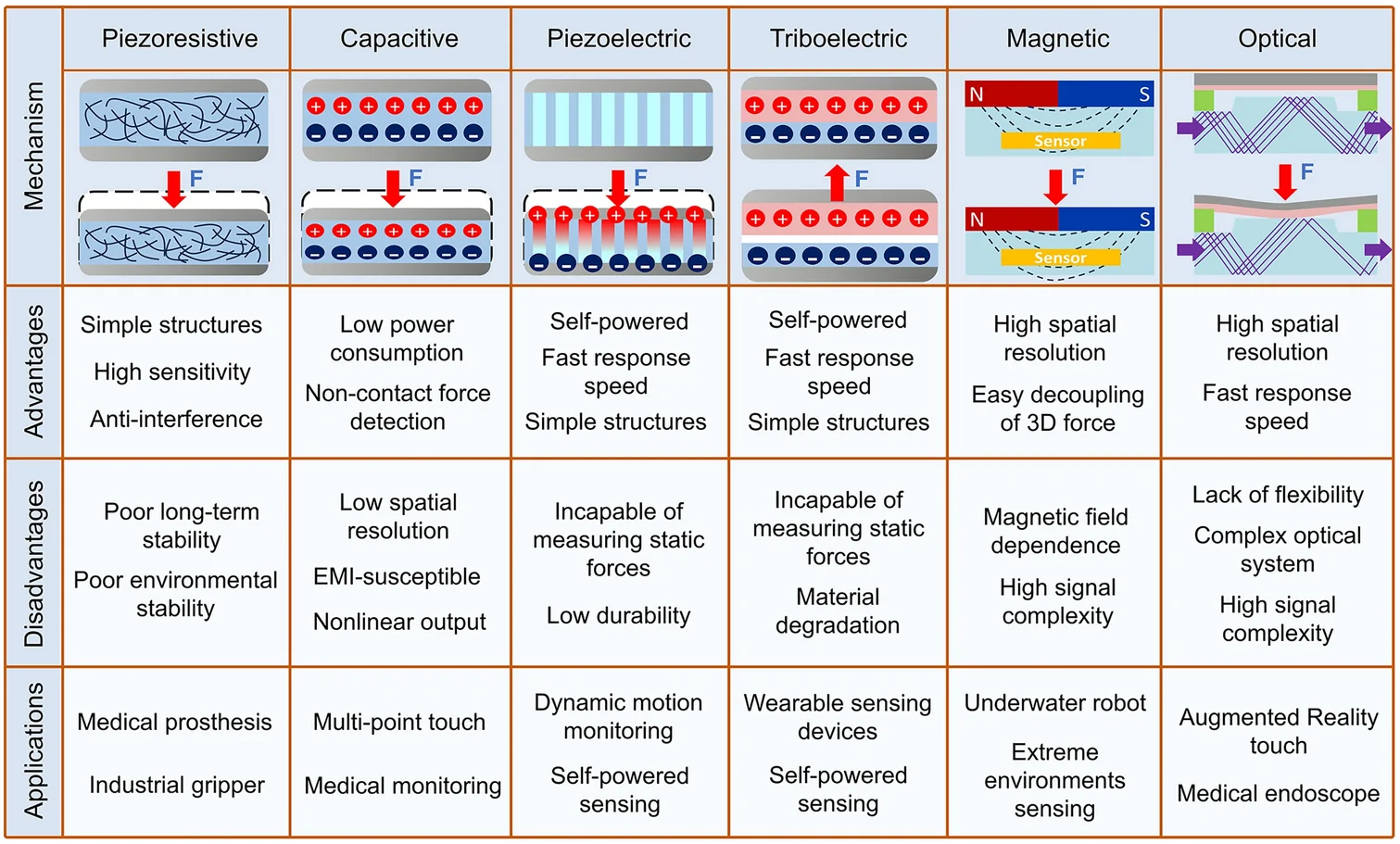
An overall summary: schematic diagrams, comparative advantages/limitations, and application scenarios across sensing mechanisms

The interactive behavior between objects in the real world depends on their weight and stiffness, the surface sensation during touch, the deformation during contact, and the way forces act when touched [41–44]. This research ultimately aims to overcome single-mechanism performance limitations, achieve full-dimensional tactile perception, and endow machines with biomimetic tactile intelligence. To achieve this goal, extensive researches have been conducted on sensor design, principles, and fabrications: (1) piezoresistance sensors [45], which are characterized by its relatively low cost. However, it is notably susceptible to temperature variations; (2) capacitance sensors [46], possessing high sensitivity, unfortunately, exhibits relatively poor anti-interference capabilities; (3) piezoelectric sensors [47, 48], which show outstanding performance in detecting dynamic forces, yet it tends to be less effective when it comes to the detection of static forces; (4) triboelectric sensors [49, 50], having the advantages include high sensitivity, simple structure, self-powered characteristics, and relatively fast response speed, but it is greatly affected by environmental factors; (5) magnetoelectric sensors [51]. The advantages of such type sensors include high sensitivity/resolution, fast response time, and anti-interference ability while drawbacks are also unsatisfactory, e.g., the high cost, magnetic field susceptibility, and complex structure; (6) optical sensors [52, 53], which reveal a strong anti-interference ability and a relatively high cost. Figure 2 presents a comparison on the working principles, advantages and limitations, and application scenarios for as-reported tactile sensors.

### Piezoresistive Sensing

Piezoresistive devices generally refer to a type of devices that achieve sensing through the changes in working resistance when the device undergoes mechanical deformation under external forces. Due to its simplicity in mechanism and structure, it is widely used in the field of tactile sensing. Resistance *R* of the working layer material of the device can be given as:

2.1.1 $$R = \, \left( {\rho L} \right)/S$$

where *ρ*, *L*, and *S* represent resistivity, length, and cross-sectional area, respectively. The gauge factor (GF) of a strain sensor is defined as (Δ*R*/*R*_0_)/ε, where ε denotes strain and *R*_0_ the initial resistance. Correspondingly, the sensitivity of a pressure sensor is given by (Δ*R*/*R*_0_)/*P*, where *P* represents the applied pressure. In the case where the dependence of resistance on pressure and strain is linear, GF is constant. The change in temperature will cause changes in the piezoresistive coefficient of the bridge arm resistance and the functional characteristics of electronic devices, and it will not be possible to maintain an ideal linear relationship between input and output characteristics. Usually, measures to eliminate the nonlinear influence of temperature are needed, namely temperature compensation [54–56].

**Fig. 3:**
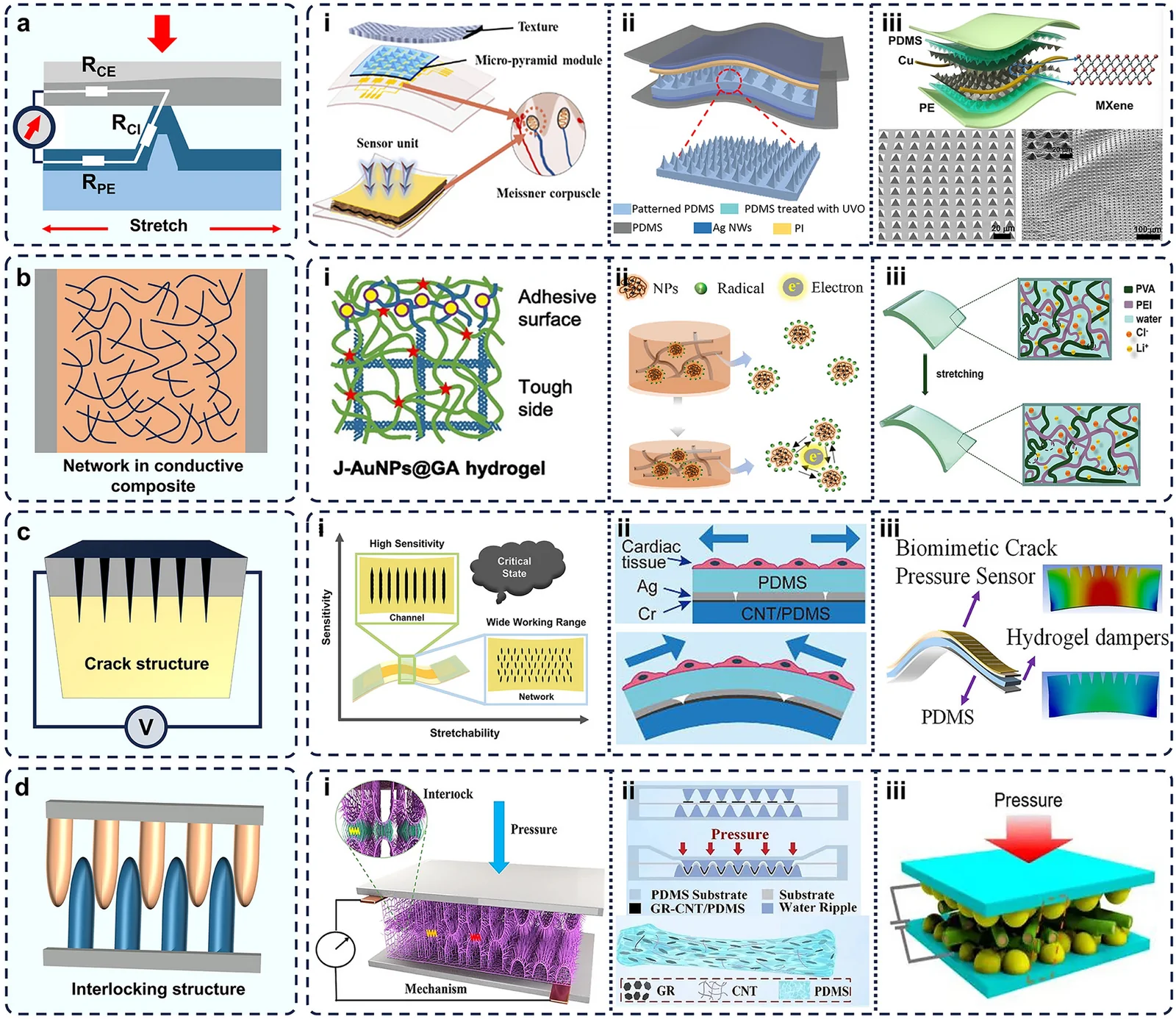
Multiple device structures based on piezoresistive principles. **a** Micropyramid structure piezoresistive devices [59–61]. Copyright 2023, WILEY–VCH. Copyright 2022, WILEY–VCH. Copyright 2022, The American Association for the Advancement of Science. **b** Internal network structure of conductive polymers [62–64]. Copyright 2021, Elsevier. Copyright 2021, Elsevier. Copyright 2020, WILEY–VCH. **c** Crack structure [68–70]. Copyright 2023, Elsevier. Copyright 2022, American Chemical Society. Copyright 2024, American Chemical Society. **d** Multilayer interlocking structure devices [71–73]. Copyright 2024, Donghua University. Copyright 2023, Wiley‐VCH. Copyright 2021, American Chemical Society.

To achieve the sensing of tangential force to normal force, researchers have developed various pyramid/cone structure arrays based on the principle of piezoresistance. Wen et al. designed multilayer microporous structures with different porosities and introduced them into sensors, enabling each sensing unit to have ultra-high sensitivity and a wide detection range, as shown in Fig. 3a(i) [57]. They developed customized micropyramid arrays and assembled them into sensor arrays to achieve the conversion from tangential force to normal force. Based on finite element analysis, Zhao et al. demonstrated that a uniform micropyramid structure was crucial for achieving ultra-high sensitivity, low detection limits, and fast response [58]. And a feasible new approach for developing micropyramid PDMS microstructures via low-cost ultraviolet ozone (UVO) irradiation technology was proposed. The schematic diagram applied to sensing devices is shown in Fig. 3a(ii). Similarly, Gou et al. designed a piezoresistive artificial eardrum device with a microcone array substrate, demonstrating the two-stage amplification effect of the cone structure in mechanical and acoustic sensing, as shown in Fig. 3a(iii) [59].

Another way to achieve changes in working resistance relies on conductive composite materials. By adding various conductive particles in the form of fillers to the insulation material, changes in the electrical resistivity of the composite material can be achieved. The change in electrical resistivity of this composite material mainly originates from the change in particle separation, which leads to the formation of internal microcircuits in the composite material under external stimulation. Zhang et al. prepared Janus dual network viscous hydrogel by guiding gold nanoparticles into gelatin polyacrylamide. The continuous three-dimensional network structure in the conductive hydrogel endowed the material with inherent flexibility and mechanical strength [60]. The preparation process is shown in Fig. 3b(i). By combining the metal nanoparticles with the gel, the composite gel would produce more connecting structures when stressed, thus realizing piezoresistive sensing, as shown in Fig. 3b(ii) [61]. In addition to the method of dispersing metal nanoparticles in conductive matrix, Wang et al. reported a binary network elastomer based on polyvinyl alcohol (PVA) and polyethylene imine (PEI) composite hydrogel [62], of which the gauge coefficient (GF) of the prepared elastomers under different tensile states closed to 22, and showed great stability under 6000 cycles. The structural concept diagram of this binary elastomer under tension and compression is shown in Fig. 3b(iii).

The inspiration of nano-crack structure comes from the slit organs on spider legs, and devices with such structure exhibit high sensitivity and ductility [63]. By pre-stretching or bending, cracks are generated on the metal film, and the resistance changes in the film caused by the opening and closing of these cracks under deformation are achieved, thereby achieving piezoresistive sensing [64, 65]. To regulate the morphology and propagation behavior of cracks, Wang et al. proposed a strain engineering strategy that by reasonably inducing the non-uniform spatial distribution of stress, and thereby controlling the generation and propagation behavior of cracks, it produced a clear crack density distribution in space [66]. Based on this strategy, the GF of the device was effectively increased from 11.40 to 690.95, as shown in Fig. 3c(i). To replace the commonly used thin and brittle metal film sensors with microcracks in the field of biosensing, Wang et al. significantly improved the stability of crack sensors by utilizing the synergistic effect of brittle ductile double-layer conductivity [67], achieving over 2,000,000 cycles in detecting weak contractility of single-layer myocardial cells. The structure of the improved device is shown in Fig. 3c(ii). Although piezoresistive devices based on crack structures have high sensitivity, they inevitably bring about noise interference issues. To solve this problem, Li et al. proposed a bionic crack pressure sensor based on a hydrogel damper [68]. This sensor had selective frequency response, with a relative change in resistance of over 200% at different vibration frequencies, as shown in Fig. 3c(iii).

Interlocking structures are widely used in piezoresistive sensor components due to their ability to generate significant changes in contact area under small deformations. Peng et al. developed a pressure sensor that combines two-dimensional materials with nanowires [69]. Such interlocking structure of the two materials can cause a significant increase in contact area under small deformation, greatly improving the sensitivity of the device, whose structure and working principle are shown in Fig. 3d(i). To achieve underwater force perception, Zhang et al. used a double interlocking water ripple structure to improve the sensitivity and force detection range of sensor components [70], as shown in Fig. 3d(ii). This sensor exhibits excellent resistance response, fast dynamic recovery, and mechanical and electrical stability in aquatic environments. Inspired by the rose structure, Yang et al. reported a flexible piezoresistive pressure sensor with high sensitivity and a wide pressure detection range [71]. Thanks to the interlocking microstructure sandwiched between graded polyaniline/polyvinylidene fluoride nanofiber (HPPNF) membranes, this bioelectronic device exhibited excellent mechanical flexibility and electrical performance, as shown in Fig. 3d(iii).

Additionally, piezoresistive devices featuring functional layers with hierarchical structures, porous architectures, and fiber-based designs have garnered significant research attentions. Hierarchical designs dynamically modulate contact area and improve stress distribution, enabling a broader linear response range [72]. Lattice, characterized by high aspect ratios and conductive network-forming capabilities, can be effectively integrated with microstructures to enhance sensitivity and mechanical adaptability [73]. Porous microstructures within piezoresive functional layers critically influence contact resistance. Their enhanced compressibility induces dynamic rearrangement of internal conductive networks under applied pressure [74].

### Capacitive Sensing

The capacitance of parallel plate capacitors can be expressed as ε*A*/*d*, where *A* is the area of a plate and *ε* and *d* are the dielectric constant and thickness of the dielectric materials between the plates, respectively, where *A* is the area of a plate and ε and *d* are the dielectric constant and thickness of the dielectric material between the plates, respectively. Similarly, the GF of a capacitive strain sensor is defined as (DC/C_0_)/ω, and the GF of a capacitive pressure sensor is defined as (DC/*C*_0_)/*P*, where ω represents strain and *P* represents pressure intensity. For elastic material-based tactile sensors, there is generally displacement of the material structure during the working process. In this case, variable *d* is used to measure the normal force and variable *A* is used to measure the shear force. Therefore, it can be regarded that both variables are able to measure strain. Researchers have developed capacitive sensors with a variety of dielectric layers, such as microcone structure, foam structure, microfiber structure, micropore structure, and micropillar structure.

**Fig. 4:**
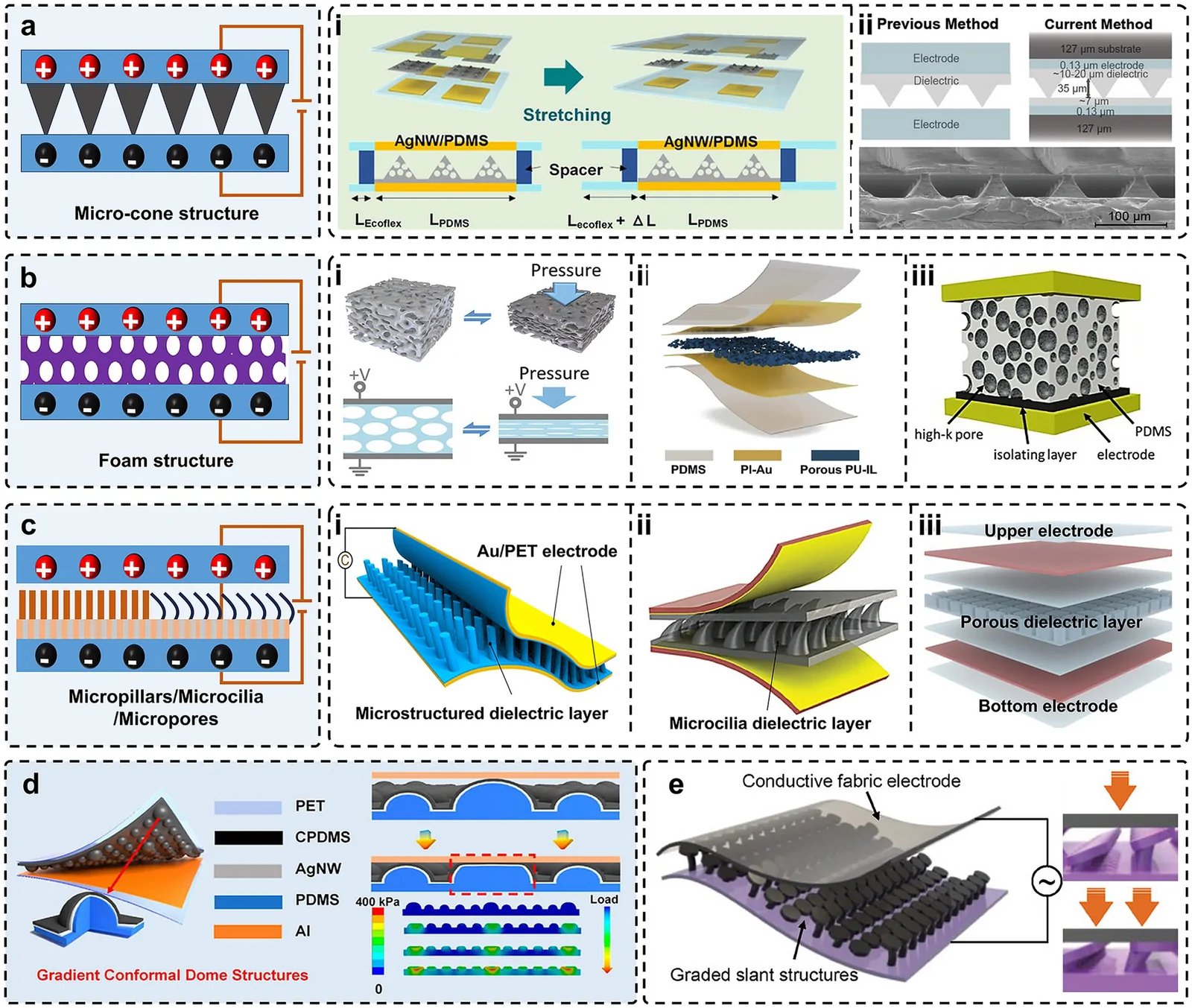
Multiple device structures based on capacitive principles. **a** Microcone/pyramid capacitive sensors [75, 76]. Copyright 2022, The Author(s). Copyright 2010, Springer Nature Limited. Copyright 2019, WILEY–VCH. **b** Foam structure capacitive sensors [77–79]. Copyright 2020, WILEY–VCH. Copyright Copyright 2021, The Author(s). Copyright 2019, The Author(s). **c** Other capacitive sensor devices with various structures, including microcolumn/microhair/micropore structure [82–84]. Copyright 2019, American Chemical Society. Copyright 2023, Wiley‐VCH. Copyright 2024, American Chemical Society. **d** Gradient conformal dome structure dielectric layer [85]. **e** Gradient slanted structure dielectric layer [86]

For capacitive sensors, the energy dissipation, which is related to viscoelastic materials and interface friction, is the main factor affecting the sensor response relaxation time. The strategy of surface microstructure construction has been frequently employed by researchers to reduce the contact area between the dielectric and the electrode, thus reducing the energy dissipation caused by interface friction and adhesion. Yang et al. reported an ultra-high-sensitivity capacitive pressure sensor based on a porous pyramidal dielectric layer, as shown in Fig. 4a(i) [75]. The enhanced sensitivity arisen from its lower compressive modulus and significant change in effective dielectric constant under pressure. Meanwhile, the quantitative parameters of microstructure design are very important for sensor structure design. Bao et al. designed a tunable pyramid microstructure capacitive pressure sensor and established a model (seen in Fig. 4a(ii)), which established an effective method for predicting the structure design and corresponding sensor performance of the sensor [76].

According to the formula, reducing the elastic modulus of dielectric layer is another effective method to improve the sensitivity of capacitive sensor. Yang et al. used foam material to soften the dielectric layer, so that the capacitor was more sensitive to touch, and the dispersed metal particles anchored within the pores of the foam structure facilitated the improvement of dielectric properties of the material [77]. The working mechanism of the composite is shown in Fig. 4b(i). Similarly, Liu et al. achieved ultra-high sensitivity of 9280 kPa^−1^ by combining high porosity and low stiffness foam with ionic liquid (IL) [78], as shown in Fig. 4b(ii). In addition, Philippe et al. proposed a similar composite foam filled with conductive carbon black particles, as shown in Fig. 4b(iii) [79].

In addition to the previously mentioned architectures, researchers have designed and fabricated advanced capacitive components featuring microscale columnar arrays, hair-like protrusions, and porous network configurations, with particular emphasis on enhancing operational stability. Because the small contact area and air gap at the interface between the microstructure dielectric layer and the counter electrode will lead to the physical separation between the layers [80, 81], Luo et al. proposed a flexible ultra-thin large-scale capacitive pressure sensor with inclined microstructures [82], as shown in Fig. 4c(i). The hair-like structure provided a novel bionic method for the flexible tactile sensor for sensing shear force and friction because of its asymmetric inclined structure. Capacitive tactile sensors with inclined microhair array (tmhas) was also proposed, which had an asymmetric structure and took the hair-like structure array as the dielectric layer [83], as shown in Fig. 4c(ii). As an efficient processing method, 3D-printing technology has been applied to the construction of patterned dielectric layer. Li et al. used 3D-printing technology to prepare an ordered porous microstructure as dielectric layer, as shown in Fig. 4c(iii) [84]. This method not only increased the compressibility and dynamic response of the dielectric layer, expanded the pressure range, but also expanded its contact area with the electrode.

Systematically controlling microstructure deformation and electrode spacing proves to be an effective strategy for simultaneously achieving high sensitivity and a broad detection range. Zhong et al. developed a gradient microdome structure that leveraged progressive deformation behavior of gradient dielectrics and a significant improvement in relative distance variation between electrodes through conformal design, thereby significantly enhancing both sensitivity and detection range, as shown in Fig. 4d [85]. Similarly, Wu et al. proposed a novel dielectric layer featuring a beetle-inspired gradient slanted structure (GSS) (Fig. 4e) [86]. The bending deformation of the tilted pillars promoted the dielectric layer with exceptional compressibility, significantly boosting the sensor's sensitivity. Concurrently, the progressive contact between the dielectric layer and electrodes achieves a broad linear response range.

Compared with resistive devices, overall, the capacitive tactile sensor has inherent advantages such as higher sensitivity, better frequency response, smaller temperature effects, and lower power consumption. However, due to the need for integration, smaller size of capacitive tactile sensors is required, which limits the sensing capacitance to the pF level. This inevitably generates parasitic capacitance, causing problems such as signal delay, low circuit speed, and low signal-to-noise ratio.

### Piezoelectric Effect

The phenomenon of piezoelectricity was first discovered in 1880, which referred to the phenomenon where a substance produced an electric current when subjected to pressure. Piezoelectric materials exhibit spontaneous polarization and are of great technological interest for a myriad of applications, notably in microelectronics, spintronics, and micro/nanoelectromechanical systems. Piezoelectricity is thought to occur through the separation of electrical charges in the lattice. The piezoelectric effect exists widely in non-centrally symmetric crystals [87–90], such as quartz, LiNbO_3_, BaTiO_3_, and PbZrO_3_(PZT), and also in some ceramics, nanomaterials, polymers, and composites, even in noncrystalline materials including deoxyribonucleic acid (DNA) [91], viral proteins [92, 93], and amino acids [94]. Piezoelectric components have become a strong competitor to tactile sensor materials due to their high sensitivity to mechanical stimuli and transient sensing ability, as they do not require external power sources.

**Fig. 5:**
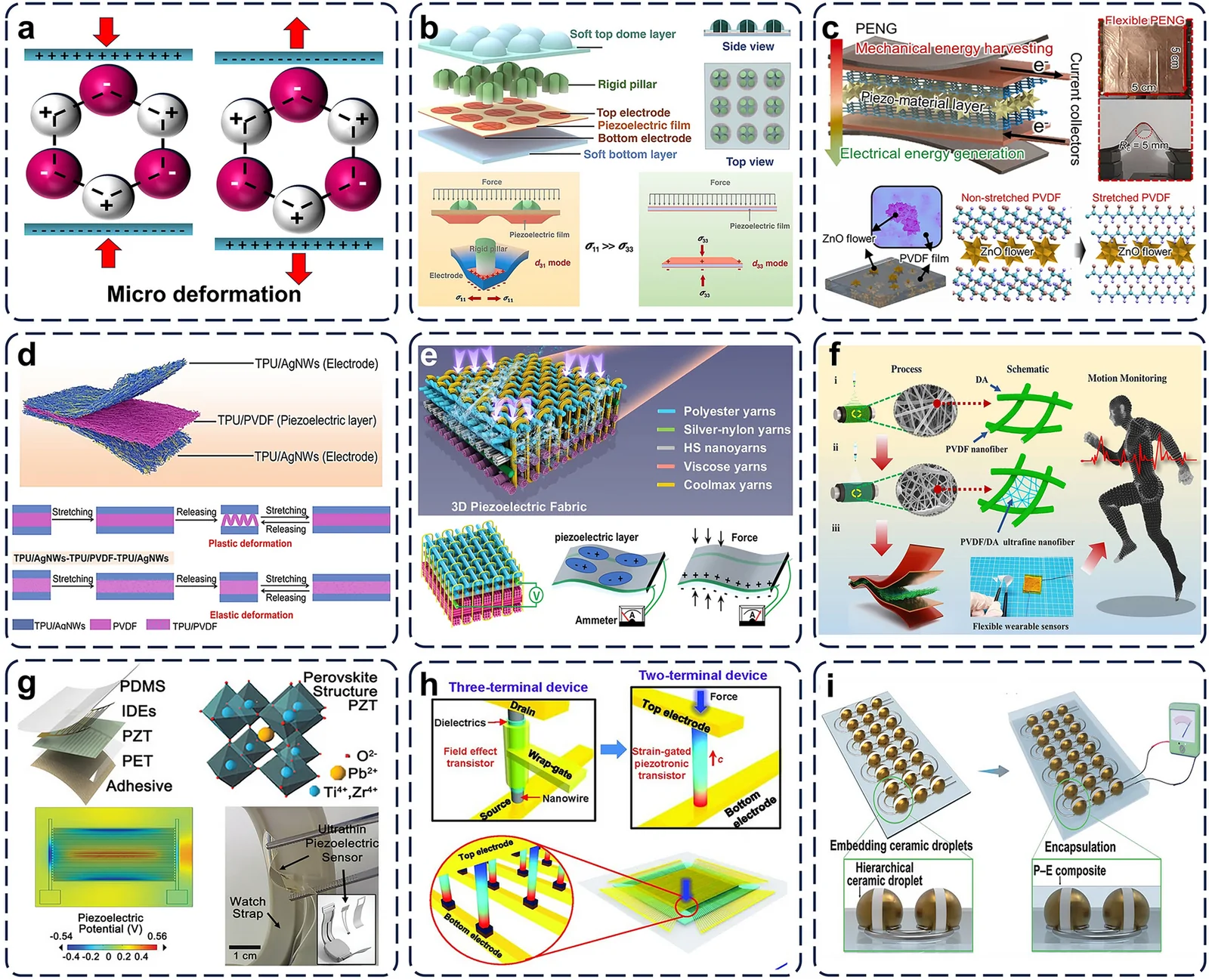
Mechanism of piezoelectric effect and tactile sensor based on piezoelectric effect. **a** Microexplanation of piezoelectric principle; **b** finger-inspired soft hard hybrid piezoelectric tactile sensor array and its working mode [95]. Copyright 2022, The Author(s). **c** PENG based on ZnO nanoflowers and PVDF [96]. Copyright 2023, Elsevier. **d** Fiber pad tactile sensor with core shell structure [97]. Copyright 2023, Elsevier. **e** 3D piezoelectric fabric sensor [98]. Copyright 2024, The Author(s). **f** PVDF/DA composite nanofiber piezoelectric sensor [99]. Copyright 2024, The Author(s). **g** PZT-based wearable piezoelectric sensor [100]. Copyright 2023, Wiley‐VCH. **h** Adaptive high-resolution tactile imaging device array [101]. Copyright 2013, The American Association for the Advancement of Science. **i** Hierarchical drop-like piezoelectric ceramic sensor [102]. Copyright 2024, The Authors

Piezoelectric materials have many working modes such as d_11_, d_33_, and d_31_. Inspired by the muscle tissue with fingertips embedded in bones, Zhang et al. prepared a piezoelectric tactile sensor using a rigid soft hybrid force transfer layer combined with a soft substrate, as shown in Fig. 5b [95]. The sensor uses the d_31_ working mode instead of the usual d_33_ mode, and thereby the ultra-high sensitivity of 346.5 pC N^−1^ at 30 Hz is obtained. With the development of modern robotic arms from rigidity to flexibility, some new piezoelectric materials with flexibility have been widely studied to replace commonly used piezoelectric materials such as quartz and ceramics in industry. Polyvinylidene fluoride (PVDF), which has the characteristics of high-voltage coefficient, simple processing technology, stable size, and chemical inertness, is a promising candidate material. The flexible characteristics make it possible to integrate embedded systems with robotic arms. Theoretically, the principle of microstructure design suitable for capacitive sensors is also suitable for piezoelectric sensors.

Piezoelectric thin films containing piezoelectric ceramics as dispersion fillers have been widely studied. In the process of the composite of ceramic powder and polymer matrix, it is necessary to consider the micromorphology of the powder, so as to regulate the polarized interface of the piezoelectric film. Bhavana et al. prepared piezoelectric sensing films on copper foil by ultrasonic spraying highly complex 3D flower-shaped ZnO and PVDF and obtained highly flexible and mechanically strong PENG films, as shown in Fig. 5c [96]. In addition, piezoelectric sensors with fiber and fabric structures were also well documented. Wei et al. prepared core–shell structured fiber piezoelectric pads of TPU and PVDF through electrospinning, showing a stretchability of 300% and a sensitivity of 20.3 mV N^−1^ (Fig. 5d) [97]. However, the tightness of the traditional wearable sensor packaging process has led to a decline in comfort. Fan et al. developed a PVDF piezoelectric nanoyarn with 313.3 MPa ultra-high strength [98]. Using advanced 3D textile technology, three-dimensional piezoelectric fabric (3DPF) sensor was woven with different yarns, as shown in Fig. 5e. The content of β phase has been proved to significantly affect the piezoelectric properties of PVDF based materials. Xiong et al. successfully prepared a high-performance composite nanofiber membrane with a coherent and uniformly dispersed two-dimensional (2D) network topology composed of polyvinylidene fluoride (PVDF)/dopamine (DA) nanofibers and ultrafine nanofibers by electrospinning technology [99]. In their work, dopamine was introduced into the matrix during electrospinning, which facilitated the formation of hydrogen bond and dipole interaction and thereby successfully induced PVDF and ultrafine PVDF nanofibers to form higher β-crystal orientation. Finally, a large piezoelectric output improvement (66.7%) was obtained, and the film structure is shown in Fig. 5f.

As a well-known perovskite piezoelectric material, PZT has been widely used in the field of sensing. Min et al. protected PZT piezoelectric film by adding polydimethylsiloxane (PDMS) passivation layer and medical grade adhesive layer and achieved high normalized sensitivity (0.062 kPa^−1^) in blood pressure sensing (Fig. 5g) [100]. Moreover, the spatial resolution of tactile behavior is an important parameter to evaluate the sensing performance. Wu et al. reported the large array three-dimensional circuit integration of piezoelectric transistors based on vertical zinc oxide nanowires, realizing shape adaptive high-resolution tactile imaging and self-powered, multidimensional active sensing, which will be effectively applied to the field of robot tactile [101]. Such array structure is shown in Fig. 5h. Also based on bionics, Xu et al. prepared a drop-shaped piezoelectric sensor with arched surface and rounded corner structure, as shown in Fig. 5i [102]. The soft circuit of the sensor was constructed by patterning technology, which showed excellent sensitivity and durability. After 5,000 tensile cycles at 60% strain and 5,000 torsional cycles at 180°, the open-circuit voltage remained stable.

### Triboelectricity Sensing

The concept of triboelectric nanogenerators originates from Maxwell displacement current, which can complete the process of converting mechanical energy into electrical energy [103, 104]. During the relative movement of two materials, the potential difference generated at the contact interface drives electrons to move between the back electrodes of the material. The mechanical energy generated by external movement is converted into electrical energy and generates electrical signals. The tactile sensor based on friction motor system has the characteristics of high sensitivity, simple structure, and wide availability of materials. However, its sensing performance largely depends on the rate of change of applied stress, so obtaining stable static pressure detection on the same device is a key issue for its applications as tactile sensing [105–107].

**Fig. 6:**
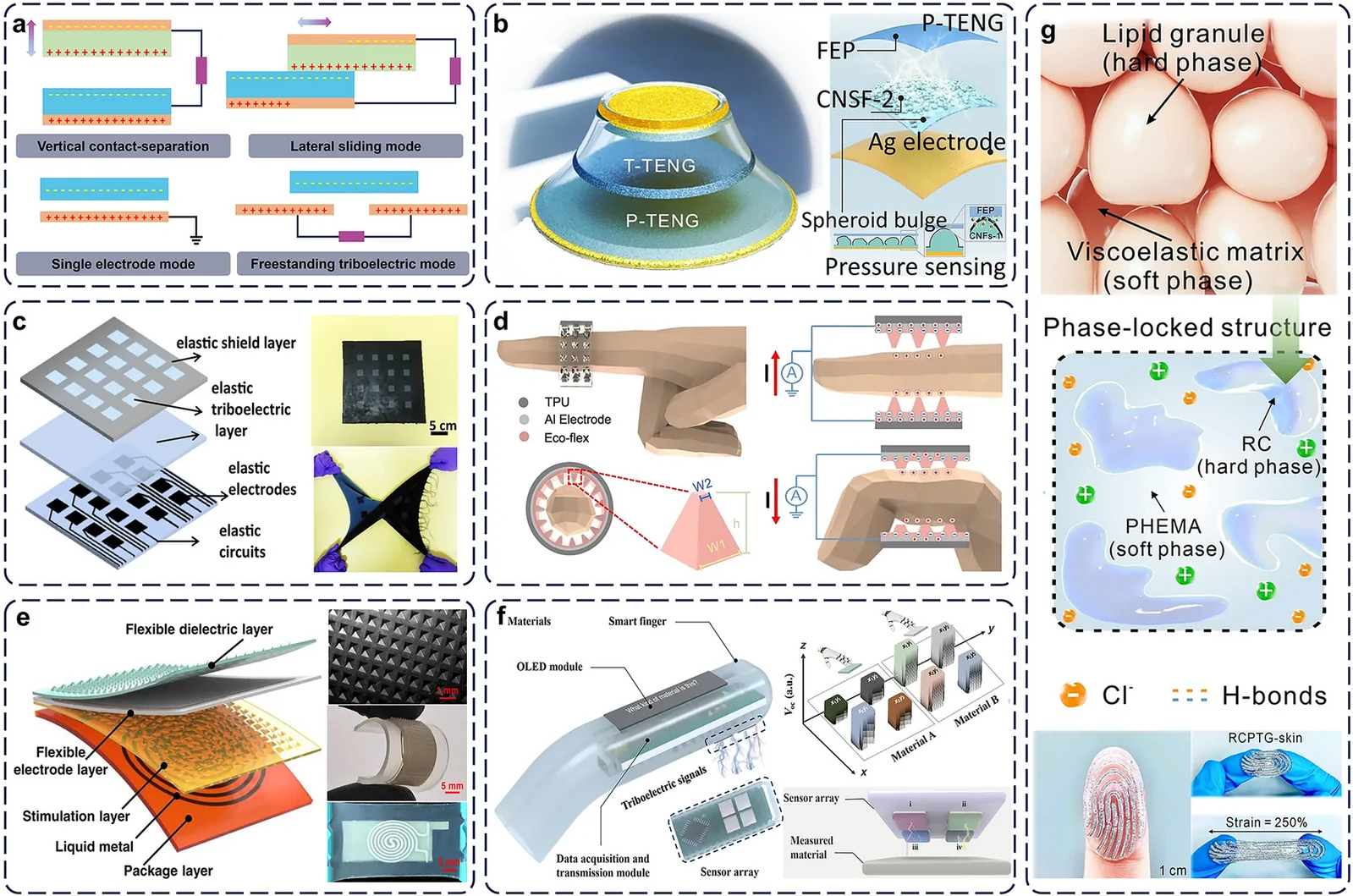
Triboelectric mechanism in tactile sensor field. **a** Four working modes of triboelectric devices. **b** Intelligent soft robot gripper system based on triboelectric nanogenerator sensor. Coprright 2023, Institute of Physics Publishing [109]. **c** Unconstrained triboelectric electronic skin (UTE skin) [110]. Copyright 2020, The Author(s). **d** ATH-Ring for continuous bending sensing [111]. Copyright 2024, The Author(s). **e** Design and sensing mechanism of the tactile/touchless flexible bimodal smart skin (FBSS) [112]. Copyright 2022, The Author(s). **f** Triboelectric intelligent finger for material identification [26]. Copyright 2022, The American Association for the Advancement of Science. **g** Iontronic triboelectric gel for tactile sensing [113]. Copyright 2024, The Author(s)

The main working modes of triboelectric devices can be categorized as four categories: contact separation mode, interlayer sliding mode, single electrode mode, and freestanding triboelectric mode. The schematic diagram of the four working modes is shown in Fig. 6a. For triboelectric tactile sensors, enhancing performance relies critically on maximizing effective contact area and optimizing surface characteristics. Therefore, hierarchical patterns, porous textures, and fiber-based micro/nanostructures are widely leveraged for this purpose. Besides, by combining friction potential with semiconductor devices, the transport of charge carriers in semiconductor channels can be directly controlled through mechanical stimulation [108]. The external force applied to TENG is converted into a voltage spike, which is then captured by a neural morphology transistor to generate a PSC response. Benefiting from the self-powering characteristics of friction electrical components, the tactile integrated system constructed based on this device has lower energy consumption and higher stimulus recognition ability.

The triboelectric nanogenerator uses soft materials with a Young's modulus that matches well with silicone rubber and TPU and has the characteristic of adaptive signal output. It has been widely applied in the field of soft robot sensing. The simplicity of signal processing circuit design makes it easy to integrate with soft or rigid actuators as tactile sensing skin. Liu et al. designed a structurally asymmetric dual-layer sensor using silicone as the outer shell to facilitate rapid response to pressure stimuli (Fig. 6b) [109]. Additionally, the chemically modified cellulose material, whose spherical surface morphology enhances triboelectric performance and sensing sensitivity, achieved a pressure sensing sensitivity of 9.21 kPa^−1^.

For large-area stretchable tactile sensing networks, the single electrode mode self-powered tactile sensor based on frictional nanogenerators is one of the best solutions with ideal properties. In order to solve the misidentification interference generated during the manufacturing process of large-area sensor array circuits, Shao et al. proposed a large-scale unconstrained multiplexed TENG tactile sensing array, which used an omnidirectional stretchable carbon black Ecoflex composite shielding layer to effectively reduce the electrostatic interference of wiring. The prepared electronic skin showed working stability under high tensile strain, which was expected to be applied to tactile electronic skin for robots, as shown in Fig. 6c [110]. Besides, perception and feedback functions are two important aspects for achieving human–computer interaction. Based on mechanisms such as frictional electricity and pyroelectric electricity, Sun et al. proposed an enhanced tactile perception and tactile feedback loop (ATH Rings) for VR applications, in which all sensors and tactile stimulators were integrated into the ATH Ring [111]. The structure of the tactile sensor is composed of a layer of silicone rubber film with a pyramid structure on one side, as shown in Fig. 6d.

Based on the non-contact working mode and the pyramid array structure of the friction layer, Liu et al. developed a flexible bimodal intelligent skin (FBSS) based on friction nanogenerator and liquid metal sensing, which was able to simultaneously perform tactile and non-contact sensing and distinguish between these two modes in real time [112]. It was implied that such pyramid-shaped flexible dielectric layer effectively increased the working charge density between the friction layers while maintaining excellent flexibility and stretchability, as shown in Fig. 6e. Based on same working mode, Qu et al. designed a frictional electric intelligent finger that surpasses human tactile perception for use in intelligent robots or artificial prostheses (Fig. 6f) [26]. Integrating multiple materials with different frictional and electrical properties into smart fingers enables recognition of different material roughness and types. Inspired by the soft-hard interlocked structure of human skin, Nie et al. reported a mechanically compliant iontronic triboelectric gel with an interlocked architecture, as illustrated in Fig. 6g [113]. The synergistic combination of low modulus, high elasticity, and strong adhesion enabled long-term conformal contact and synchronous deformation with working interfaces.

### Magnetic Induction

The key components of a flexible magnetic tactile sensor are the magnetic film and Hall element. The magnetic film (sensing module) can convert the position and magnitude of external forces into changes in the magnetic field, and the Hall element plays a role in signal reception and processing. Due to the decoupling perception of magnetic tactile sensing elements, which can be integrated into Hall elements, wireless transmission of signals can be achieved. Magnetic tactile sensors have excellent resistance to electromagnetic interference, but their cost increases due to the intrinsic characteristics of material.

**Fig. 7:**
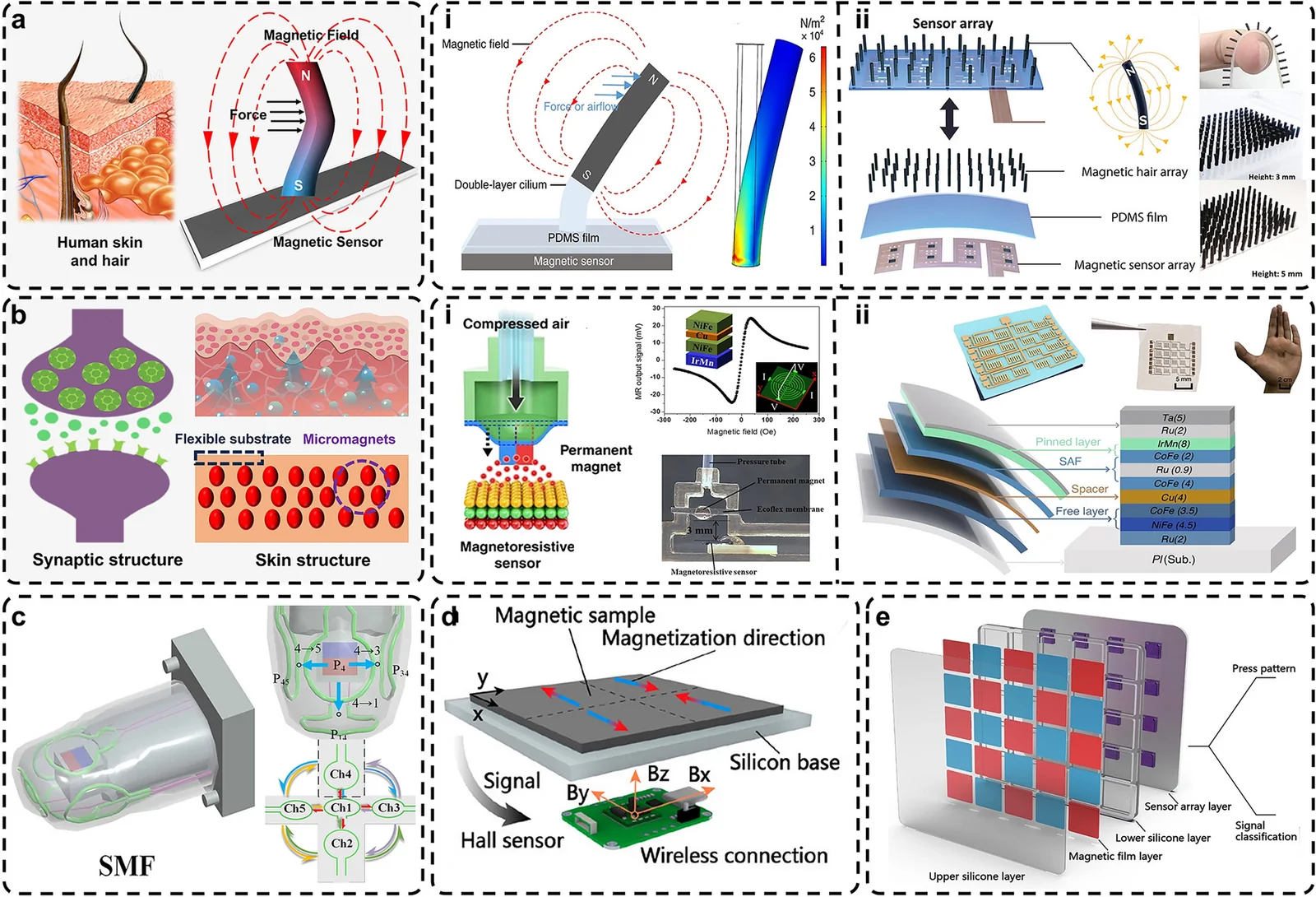
Magnetic tactile sensor with various structures. **a** Magnetic ciliary structure similar to human skin cilia: **i** Double-layer flexible magnetic cilia [114]. Copyright 2023, The Author(s). (ii) Magnetic cilia array sensor [115]. Copyright 2024, The Authors. **b** Magnetic tactile sensor mimicking synapse and skin: i Tactile tip magnetic synapse integrated system [118]. Copyright 2017, The Author(s). (ii) Tactile sensor based on giant magnetoimpedance (GMI) material [119]. Copyright 2024, The Author(s). **c** Soft magnetoelectric finger for robot multidirectional tactile [120]. Copyright 2024, The Author(s). **d** Tactile sensor composed of multidirectional magnetized flexible film and non-contact Hall sensor [121]. Copyright 2022, American Chemical Society. **e** Magnetoelectric tactile sensor with sandwich structure with super-resolution and self-decoupling function [122]. Copyright 2021, The American Association for the Advancement of Science

Ciliary structures, such as sweat and eyelashes, are considered to be the most sensitive structures in nature. The magnetic cilia can realize sensitive tactile perception with small changes of magnetic field during the bending process. Magnetic ciliated tactile sensor has been also studied widely, as shown in Fig. 7a. Demolding method is the most commonly used method for preparing magnetic cilia. First, the hard mold is perforated by laser processing or photolithography etching. Then, the mixture of flexible matrix and magnetic particles is poured into the hole in the mold. After solidification, the mixture is stripped from the mold to form cilia. However, magnetic cilia based on this method inevitably reveal the limitation of the content of magnetic particles, which leads to the inability to maximize the magnetism of cilia. In addition, the high content of magnetic particles also caused the destruction of ciliary flexibility. To solve this problem, Chen et al. developed a double-layer magnetic ciliary sensor [114]. The upper layer of the cilia was a flexible material mixed with magnetic particles, while the lower layer was a pure flexible material. This double-layer structure significantly improved magnetism while maintaining the flexibility of cilia, as shown in Fig. 7a(i).

For robot tactile sensors, large-area and multidimensional sensing are two important criteria. Therefore, researchers prepared the magnetic cilia array in order to achieve large-area and multidimensional tactile sensing. Chen et al. arrayed the magnetic cilia and developed a flexible tactile sensor based on the magnetic cilia array, which had high sensitivity and stability [115]. The upper layer of the sensor was a plurality of magnetic cilia containing magnetic particles, while the lower layer was a serpentine flexible circuit board with a magnetic sensor array. When the magnetic cilia were forced to bend, the magnetic sensor array detected the change of the magnetic field, so as to obtain the magnitude and direction of the external force. This proposed sensor showed a resolution of 0.2 mN and a working range of 0–19.5 mN and had the ability to identify the direction of external force. The magnetic cilia array is shown in Fig. 7a(ii).

Human skin has a variety of functions to detect external stimuli, such as pressure, shear force, tension, sliding, vibration, and temperature, with high accuracy and reliability [116, 117]. The sensing signals of the mechanoreceptors in the fingertips are transmitted through nerve cells through non-contact methods in synapses and finally processed by the central nervous system. Inspired by the working structure of synapses, Lim et al. developed a tactile sensing system integrated by touch tips and magnetic synapses. The tactile sensing system consisted of a remote touch tip that generated air pressure through external touch, an air tube that provided the generated air pressure, and a magnetic synapse that converted air pressure into electrical signals, as shown in Fig. 7b(i) [118]. It can realize tactile stimulation with a minimum detection pressure of 6 Pa and a maximum frequency of 1,000 Hz. At the same time, human skin also has a very high response ability to weak pressure stimuli, which constitutes a wide range of human tactile perception. Therefore, the biomimetic tactile sensing system integrating stimulus sensing and neuron like information processing function under low pressure has become a hot spot for researchers. Zhang et al. developed a novel magnetoelectronic, touchless interactive system utilizing a flexible giant magnetoresistive (GMR) sensor array, which was fabricated using an electrochemical delamination process [119]. The ultra-thin flexible electronic system possessed both ultra-thin and non-destructive characteristics. The flexible magnetic sensor was capable of achieving a bending angle of up to 90°, maintaining its performance integrity even after multiple repetitive bending cycles, as shown in Fig. 7b(ii).

In addition to the above special structure, researchers have also made many explorations on magnetoelectric tactile sensors for robot tactile applications. Human fingertips are the most commonly used tactile sensing parts. For robots, tactile perception in a variety of complex environments is an important direction beyond human perception. In addition to the above special structure, researchers have also made many explorations on magnetoelectric tactile sensors for robot tactile applications. Human fingertips are the most commonly used tactile sensing parts. For robots, tactile perception in a variety of complex environments is an important direction beyond human perception. Xu et al. showed a soft magnetoelectric finger (SMF) based on LM coil array, which was capable of realizing self-generated signals and multidirectional tactile sensing [120]. The SMF was composed of a "finger" skin-like flexible sheath, which contained five LM coils and a designed "phalanx," with magnets inside. In order to realize tactile perception in an environment that cannot be visualized, SMF had been integrated into the manipulator to explore the black box of design, including identifying obstacles on the wall/ground and accurately judging the type of objects, as shown in Fig. 7c.

Magnetoelectric sandwich tactile sensor has also attracted the attentions of researchers because of its simple structure and the ease of preparation. Its flexible characteristics enable it to be applied in different working environments. Hu et al. proposed a wireless flexible magnetic tactile sensor (FMTs), which was composed of a multidirectional magnetized flexible film (sensing module) and a non-contact Hall sensor (signal receiving module) [121]. This flexible magnetic film was constructed by NdFeB particles and soft silicone elastomer particles. It converted the clear transduction of the position and size of the external force into magnetic signals. Such device structure was capable of array integration, as shown in Fig. 7d. Similarly, Hu et al. introduced a soft tactile sensor, which had super-resolution and self-decoupling functions, and could imitate the force sensing ability of human skin [122]. These characteristics were realized by sandwich structure, which was composed of flexible magnetic film, silicone elastomer layer, and Hall sensor. It had the ability of self-decoupling and super-resolution, as shown in Fig. 7e.

### Optical Sensing

The light source, photosensitive elements, and flexible touch layer are the main components of optical tactile sensors, which have the advantages of low cost, easy miniaturization, and integration. Flexible optical fiber materials are widely used in the field of tactile sensing due to their inherent electrical isolation characteristics. So far, researchers have developed various types of tactile sensors using photosensitive components, such as fiber Bragg gratings [123], interferometers, and micro/nanofibers [124, 125]. To endow sensors with mechanical flexibility and variability, photosensitive components are typically encapsulated in polymer materials.

**Fig. 8:**
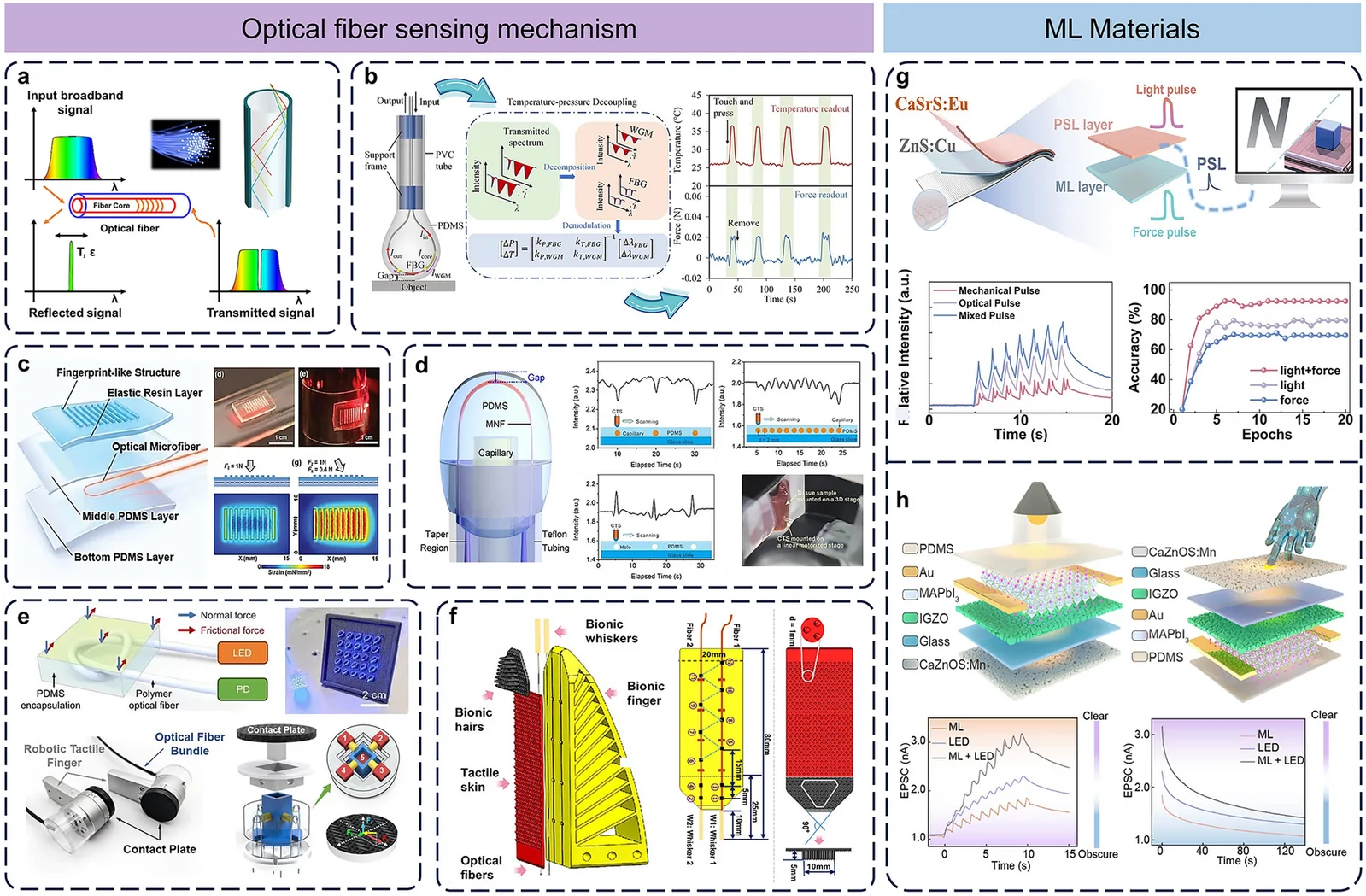
**a** Tactile sensing mechanism of optical fiber materials; **b** SBFT sensor fabricated by encapsulating a macrobent fiber Bragg grating (FBG) in an elastomeric droplet [126]. Copyright 2023, American Chemical Society. **c** Finger-skin-inspired flexible optical sensor and working mechanism [128]. Copyright 2021, Wiley‐VCH. **d** MNF-embedded compact tactile sensor [127]. Copyright 2021, American Chemical Society. **e** Construct optical fiber knot (OFN) sensors and Exploded diagram of the robotic tactile finger [129]. Copyright 2023, The author(s). **f** The bionic finger based on optical fiber sensor implemented grab operation [130]. Copyright 2024, The author(s). **g**, **h** Mechanoluminescent-based tactile sensing system [131, 132]

The sensing principle of FBG depends on Bragg wavelength modulation in response to external physical stimuli (such as strain, temperature, and pressure). When subjected to such stimulation, the mechanical deformation or refractive index change of the grating will change its periodicity, thus changing the Bragg wavelength, which reflects the size and nature of the applied stimulation. In addition, the integration of multiple gratings in a single fiber makes it possible to synchronously measure various physical parameters, and realizes the diversification of sensing information. By combining optical fiber with PDMS matrix, Shang et al. proposed the design and manufacture of a soft bionic optical fiber tactile (SBFT) sensor, which could simultaneously sense and distinguish the temperature and pressure stimuli. The sensor structure is shown in Fig. 8b [126]. The PDMS substrate in the device structure showed high thermal optical coefficient and low elastic modulus, which substantially improved the sensitivity of pressure and temperature sensing. The pressure and temperature could be completely decoupled with high accuracy of 0.2 °C and 0.8 mN, respectively. Similarly, with polymer materials as the matrix and curved optical fibers as the sensing materials, Tang et al. introduced a compact tactile sensor (CTS) with a diameter of 1.5 mm, which can transmit touch and pressure stimuli into interpretable optical signals with high fidelity, and was expected to be applied in the fields of minimally invasive surgery and palpation information acquisition. The structure diagram of the device is shown in Fig. 8d [127].

Because of its unique structure and high sensitivity, human finger skin has become the design template of a new generation of tactile sensors. It simultaneously features multiple sensing functions, incorporating physical quantities that are hard to sense, such as hydrostatic pressure and shear force within a specific area. In order to achieve more advanced sensing, large-scale force sensing and sliding force detection have become a major challenge. Jiang et al. proposed a flexible tactile sensor based on optical microfibers inspired by finger skin (FIFO) for force sensing and sliding detection in the grasping process of manipulator, as shown in Fig. 8c [128]. The parallel ridge structure introduced in the device structure was used to simulate the fingerprint structure of human fingertips. The optical microfibers encapsulated in soft layers with different rigidity detected external stimuli in a way similar to the sensory receptors and nerve fibers in the skin. The mechanical hand equipped with bionic sensors was able to detect and distinguish contact force and sliding. Researchers pointed out that the key to decoupling the normal force and friction was the anisotropy of the sensor structure or material. A cost-effective friction sensing strategy based on optical fiber knots (OFN) was proposed [129]. It is suggested that the knotted structure changes the load distribution along the fiber, making a single polymer fiber sensitive to normal force and friction. The device based on optical fiber knot structure is shown in Fig. 8e. The OFN sensors achieved high sensitivity in both normal force detection and friction force detection, with values of 2.67 and 5.59 N^−1^, respectively.

In addition, to further realize the application of fiber optic tactile sensing in the field of soft robots, Mao et al. proposed a novel multimodal tactile sensing soft robot finger, as shown in Fig. 8f. This robotic finger integrated a distributed fiber optic sensing system as part of its tactile sensory nervous system [130]. By training a neural network model, the mechanical finger achieved accurate recognition in terms of roughness, material stiffness, and finger pad position. Meanwhile, it demonstrated a perceptual ability that surpasses human touch in specific operational requirements, such as transferring fragile objects and efficiently picking up underwater objects.

Mechanoluminescent materials, also referred to as mechanochemiluminescent materials, are a kind of special materials capable of directly converting mechanical energy into light energy when subjected to mechanical stimuli like compression, impact, friction and stretching, thus producing luminescence phenomena. Mechanoluminescent (ML) materials enable self-powered operation and autonomous recovery through direct force-to-light conversion, eliminating external excitation sources, thus offering significant potential for scalable visuo-tactile perception arrays. The integration of photoreceptors with optoelectronic synaptic devices presents a promising approach for advancing tactile sensing systems. Pan et al. developed a bimodal sensing architecture combining ML materials with IGZO/(MAPbI)₃ heterostructure-based optoelectronic synapses, achieving integrated visual-tactile information acquisition, processing, and neuromorphic learning/memory functions (Fig. 8g) [131]. The hybrid structure demonstrated spectrally tunable synaptic responses across the visible spectrum, enabling neuromorphic adaptation to optical stimuli. Building upon analogous design principles, Hao et al. demonstrated direct mechano-optical transduction through ZnS:Cu-based ML composites, with emitted photons subsequently coupled to a photostimulated luminescence (PSL) active layer utilizing CaSrS:Eu phosphors (Fig. 8h) [132]. This innovative circuit-free architecture achieved synergistic visuo-tactile perception integration while exhibiting considerable potential for complex signal processing applications.

### Hybrid Mechanisms

**Fig. 9:**
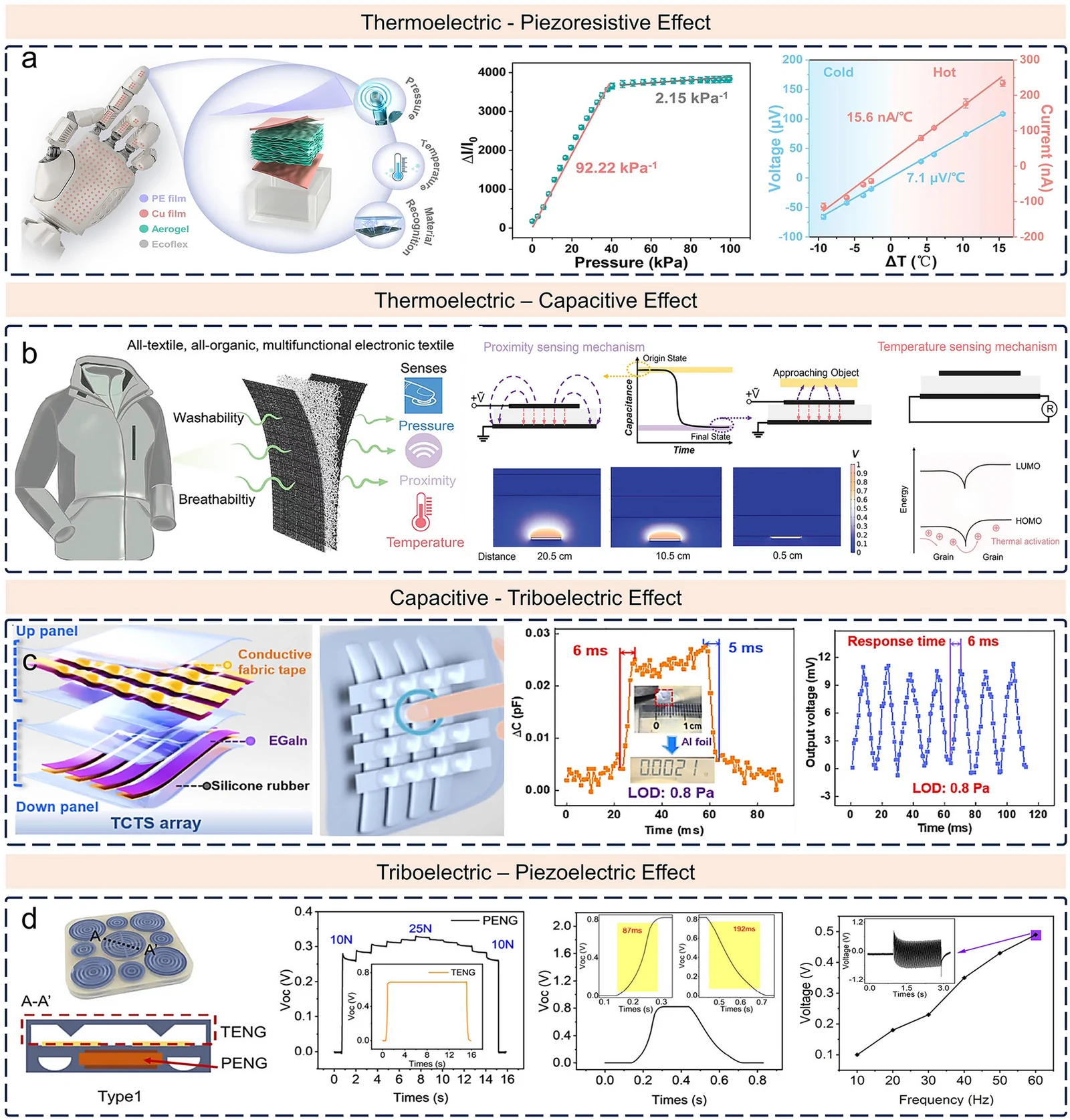
**a** The aerogel tactile sensor combined thermoelectric and piezoresistive effects [133]. **b** A tactile sensor with thermal response and pressure/proximity response capabilities [134]. **c** Integrating capacitive sensing arrays and frictional electric sensors through a multilayer structure [135]. **d** A piezoelectric frictional synergistic tactile sensor with the ability to perceive dynamic and static stimuli [136]

In multimodal tactile sensing, integration of multiple transduction mechanisms enables crosstalk-free simultaneous detection of diverse stimuli. To date, synergistic thermoelectric effects have been successfully employed to achieve decoupled temperature and pressure sensing. For example, Zhao et al. developed a tactile sensor based on the thermoelectric-piezoresistive coupling effect in nanolaminated carbon aerogels [133]. Here, the piezoresistive response arises from force-induced compression enhancing conductive pathways, while the thermoelectric effect stems from temperature gradient-driven carrier migration, converting thermal signals into electrical outputs (Fig. 9a). Similarly, Ye et al. designed a multifunctional sensor using cotton fabric as an active pressure/proximity layer, integrated with thermoresponsive conductive polymer textile electrodes in a sandwich architecture [134]. This approach enabled simultaneous detection of pressure, proximity, and temperature (Fig. 9b).

Meanwhile, the cooperative interplay between piezoelectric, triboelectric, and other mechanisms has also been leveraged to distinguish dynamic and static mechanical stimuli. Xie et al. developed a dual-mode flexible triboelectric-capacitive tactile sensor (TCTS) array [135]. This multilayered device integrated a capacitive sensor array for static load distribution mapping with a triboelectric component for dynamic pressure variation detection (Fig. 9c). Based on the synergy of triboelectric and piezoelectric effects, Liu et al. synergized piezoelectric sensing with triboelectric multi-dimensional detection and embedded pneumatic feedback, achieving simultaneous static/dynamic signal acquisition (Fig. 9d) [136].

## Summary and Outlook

The core distinction among tactile sensors with different mechanisms lies in the distinct changes, that is, they exhibit in specific parameters (e.g., resistance, capacitance, magnetic field) in response to external stimuli. Among these, optical tactile sensors demonstrate the most remarkable overall development potential for high-end applications, while triboelectric sensors show significant promise in the realm of self-powered Internet of Things (IoT). For optical tactile sensors, ultra-high resolution represents their irreplaceable core advantage. Advancements in micro-nano-optical devices, high-speed imaging chips, and AI-based image processing have significantly enhanced their potential in applications demanding fine tactile perception (such as prosthetics and precision manipulation). Their inherent synergy with machine vision constitutes a significant advantage. The disruptive advantage of triboelectric tactile sensors is their self-powering capability; however, the quantification of signal stability and sensing precision remains the critical challenge in requiring urgent resolution. Furthermore, the dynamic energy harvesting capability of piezoelectric sensors and the high environmental adaptability of magnetoelectric sensors are also notably advantageous.

The robustness of functional devices is a key performance metric of significant research focus, which has evaluation dimensions depending on the application scenario. Magnetoelectric sensors demonstrate the highest overall robustness in extreme environments. Conversely, optical sensors exhibit superior robustness in high-interference environments. Moreover, sensors based on different mechanisms exhibit unique advantages in collecting various types of multimodal signals. This indicates that single-mechanism sensors are sometimes insufficient for complex applications, making multimodal fusion an inevitable trend.

## Primitive Structural Configuration and Inspired Structure

The primitive structure configuration of a tactile sensor refers to the minimal unit essential for the core process of converting tactile stimuli into measurable electrical signals. This configuration typically comprises three fundamental components:

1. Sensing elements: The foremost component that directly perceives and responds to measurand variations (including, but not limited to, pressure, temperature, and humidity), inducing changes in its intrinsic properties (e.g., resistance or capacitance alterations).
2. Transduction element generates corresponding electrical signals synchronized with the state changes of the sensing element.
3. Minimalist circuit provides essential operating conditions for the sensing/transduction elements and extracts primary electrical signals. Examples include voltage divider circuits for resistive tactile sensors and wheatstone bridge configurations for capacitive tactile sensors.

The original structural configuration serves as the starting point for the development of flexible electronics technology and is already widely researched and understood. Currently, the research focus in the field of flexible tactile sensors is shifting toward the combination of high performance, multifunctionality, high integration, biomimetic tissue-like properties, and intelligent interaction with the human body/environment. Various bio-inspired structures have driven flexible tactile sensors beyond fundamental performance, enabling the realization of bio-system-like functionalities.

The unique physiological structures of the human body and other organisms in nature have brought many inspirations to the structural design of tactile sensors. Mechanoreceptors within human skin are responsible for discriminating a variety of mechanical stimuli, including the fast-adapting (FA) receptors (Meissner and Pacinian corpuscle) that respond to dynamic forces [137, 138] and the slow-adapting (SA) receptors (Merkel disk and Ruffini endings) that can detect static pressures[139, 140]. In addition, some arthropods (such as spiders and octopuses) are known for their limb structures that provide powerful sensing capabilities, while some plants (such as roses and lotus) are well known for their superhydrophobic surfaces. Researchers have successfully combined the principles of bionics with the structural design of tactile sensors to design and develop many high-performance flexible tactile sensors.

Meanwhile, with the rapid development of wearable devices, electronic fabric tactile sensors have received widespread attention. As the "second skin" of the human body, textiles have a soft and close fitting wearing performance, as well as a flexible and versatile structure, making them a good carrier for various electronic components in smart wearable devices. Flexible fabric tactile sensors are integrated with textiles as substrates and sensing materials or components in different ways to meet the needs of various wearable devices. This type of sensors not only meets the physical and mechanical properties of the sensor, but also maintains the texture and flexibility of the fabric, demonstrating enormous potential for application in the field of robot tactile sensing.

### Skin-Inspired Structures

There are mainly seven types of sensory receptors in the human skin: pain receptors, cold receptors, warm receptors, and four types of mechanical stimulus receptors [141]. Four types of mechanical sensors can measure forces at different time and spatial scales, with slow adaptation receptors (SA-I and SA-II) responding to static pressure while fast adaptation receptors (FA-I and FA-II) responding to dynamic pressure (pressure derivative over time) and fluctuations. So far, humanoid skin tactile sensor devices with various structures, materials, and mechanisms have been proposed, and many artificial somatosensory systems with both tactile perception and real-time feedback capabilities have been developed.

**Fig. 10:**
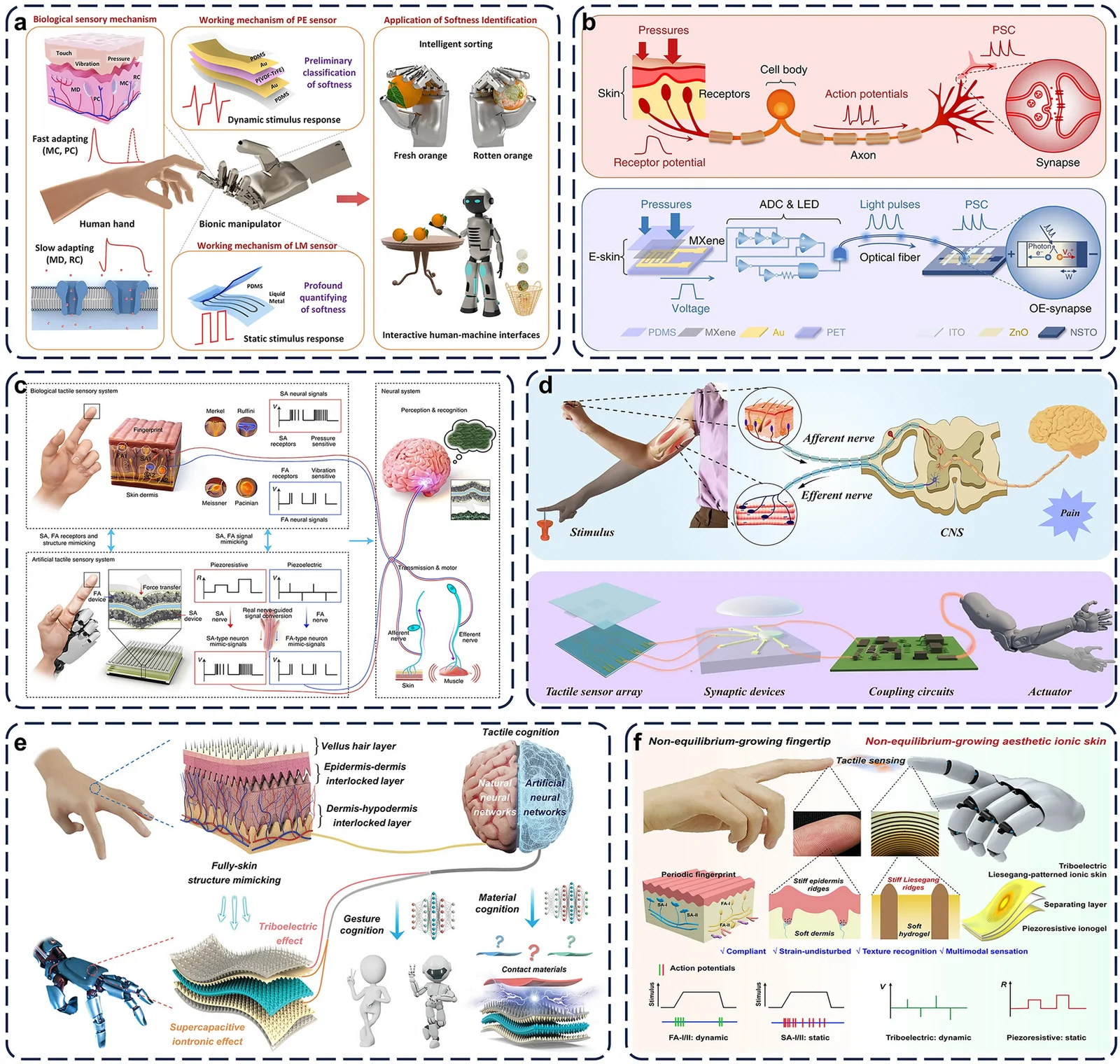
Haptic sensing system inspired by human tactile sensing mechanism. **a** Bionic multisensory electronic skin [142]. Copyright 2022, The Author(s). **b** Schematic representation of a biological afferent nerve and an artificial afferent nervous system based on the integration of Mxene and light emitting diodes (LEDs) [143]. Copyright 2020, The Author(s). **c** An artificial tactile system that integrates piezoresistive and piezoelectric effects [144]. Copyright 2021, Springer Nature. **d** Artificial sensory systems that mimic biological pain perception systems [145]. Copyright 2022, The Author(s). **e** Full-skin bionic structure e-skin [146]. Copyright 2022, Wiley‐VCH. **f** Artificial ionic skin [147]. Copyright 2023, Wiley‐VCH

Achieving the goal of noninvasive quantitative identification of softness in soft materials is still a challenging task, Qiu et al. realized non-destructive identification of material softness through a bionic multifunctional sensing system integrating piezoelectric and strain sensing modules [142]. The piezoelectric module integrated in the bionic robotic hand was inspired by the physiological function of human skin and mimiced the perceptual function of FA receptors to detect high-frequency dynamic stimuli, enabling initial differentiation of softness through transient pulse signals (Fig. 10a). Meanwhile, the piezoresistive module captured static stimuli similar to SA mechanoreceptors, enabling the liquid metal (LM) sensor to maintain the signal from the beginning to the end of the pressure.

Also inspired by biosensory systems, Tan et al. reported an optoelectronic pulse afferent nerve with neural coding, perceptual learning and memory capabilities that mimic tactile sensing and processing (Fig. 10b) [143]. The artificial system mimiced a biological SA-I afferent nerve by detecting pressure information through multiple MXene-based flexible receivers, converting and encoding the detected information into light spikes by coupling light-emitting diodes (LEDs) to a ring oscillator and an edge detector (used as a special digital-to-analog converter ADC), and then the encoded optical spikes were then integrated using ITO/ZnO/NSTO-based optoelectronic synapses (OE synapses). Due to the advantage of non-contact integration, this allowed photometric memory thyristors to process multiple sensory inputs via optical spikes.

Besides, a human-like artificial neurotactile skin system was reported by Chun et al. The system consisted of sensors that mimiced both SA and FA mechanoreceptors and a neurostimulator that achieved simultaneous detection of pressure and vibration (Fig. 10c) [144]. A human-skin sensing thin film was fabricated by adding conductive piezoresistive (reduced graphene oxide flakes) and piezoelectric particles (BaTiO_3_) to an elastic polymer matrix. BaTiO_3_ particles generate piezoelectric potential under physical stimulation, sensitively detecting vibration (or high-frequency dynamic pressure), imitating FA mechanical receptors, and acting as an energy source. The rGO film mimiced the SA mechanical sensor and utilized the difference in conductivity between densely connected rGO films to achieve sensitive piezoresistive response to static pressure. To further achieve tactile feedback function, Sun et al. developed a biomimetic artificial somatosensory system consisting of four main components: flexible tactile sensors, multigate synaptic transistors, coupling circuits, and artificial muscles (Fig. 10d) [145]. It combined perception, encoding, and processing of tactile stimuli and thereby triggers the activity of artificial muscles when strong stimuli are detected.

The structural characteristics of the human chorion, epidermis, dermis, and subcutaneous tissue are of significant importance in the composition of human tactile sensation. Niu et al. proposed an artificial intelligence (AI)-driven full-skin simulation (FSB) electronic skin (Fig. 10e) [146]. This FSB e-skin was an assembly of a triboelectric e-skin stacked with a piezocapacitive (supercapacitive iontronic) e-skin exhibiting a three-layer structure configuration: A double-sided heterogeneous top layer was used to imitate the human vellus hair and epidermis; a middle layer based on double-sided layered microcone structure (LMS) ionic gel was used to imitate the skin dermis; and a bottom layer of single-sided LMS was utilized to imitate the skin hypodermis. By combining FSB e-skin with six layer MLP neural network technology and various units such as signal acquisition, transmission, processing, and display functions, an advanced intelligent material recognition system had been constructed.

In order to synergistically apply the seemingly contradictory characteristics of structural compliance, strain undisturbed pressure sensitivity, and fine texture recognition for advanced tactile sensing, Qiao et al. were inspired by the unique hard soft hybrid structure of human fingertips and designed an artificial ionic skin with tactile abilities similar to human fingertips (Fig. 10f) [147]. The pressure sensitivity under high strain was finally obtained by simulating the fingerprint like periodic hard ridge embedded in soft hydrogel matrix. Fine texture recognition of contact objects was achieved through the vibration of periodic hard ridges in the structure.

### Fabric-Inspired Structures

Due to its lightweight, flexibility, low cost, and ease of processing, textile structures have been widely used as substrates or functional materials for tactile sensors. The common tactile sensors for fabrics are mainly divided into three categories: (1) smart fibers are directly made into 2D fabrics through weaving, knitting, weaving and other methods; (2) sensor components can be obtained by coating functional materials on commercial fabrics, which can be achieved through technologies such as inkjet printing, screen printing, and 3D printing; (3) using natural fabrics such as cotton, linen, and silk to obtain a complete conductive network through high-temperature carbonization process, thereby achieving tactile sensing function. In recent years, fabric tactile sensors with diverse structures have been widely studied.

**Fig. 11:**
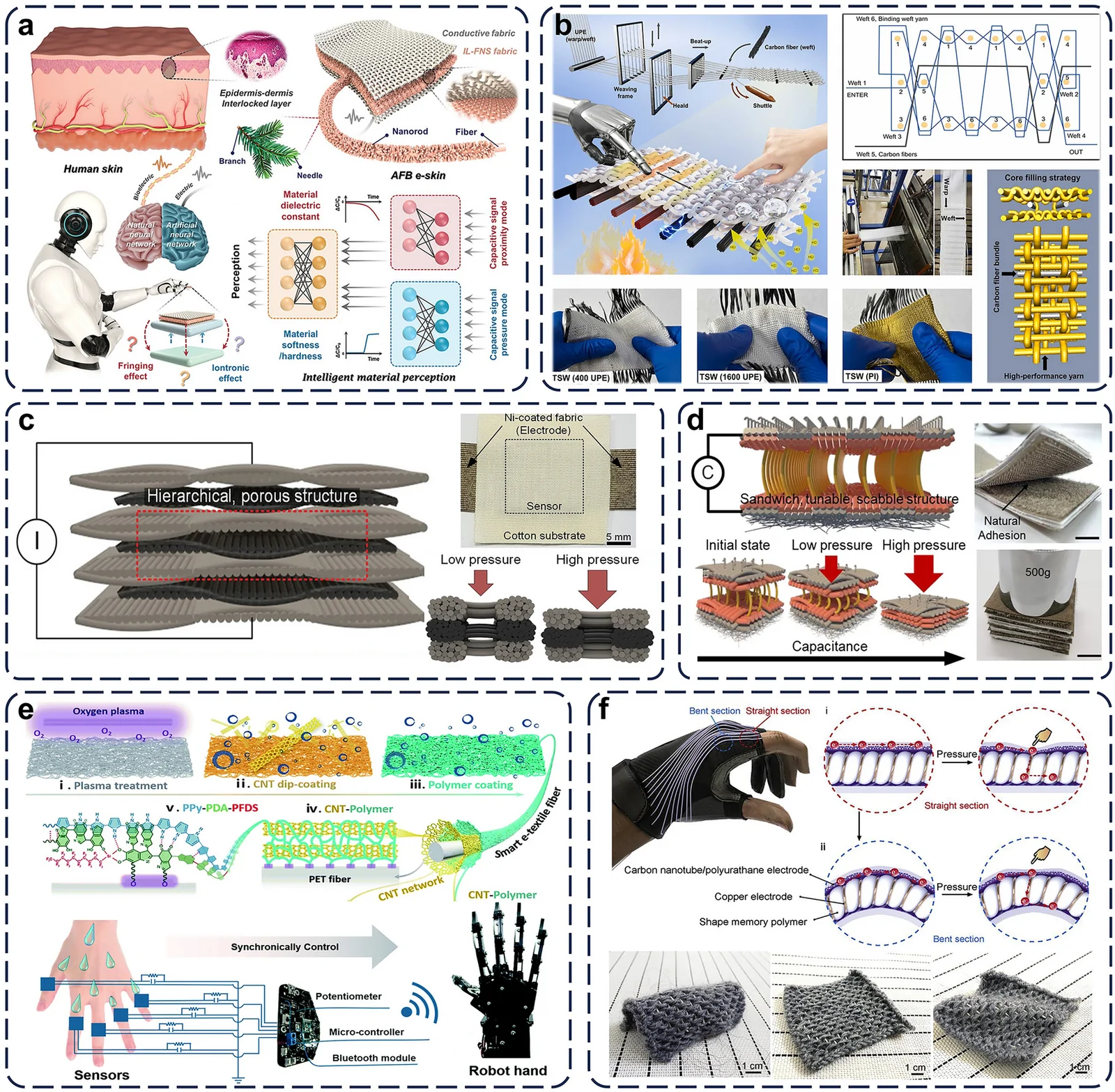
Fabric structure tactile sensor. **a** Artificial intelligence (AI)-motivated all-fabric bionic (AFB) e-skin [148]. Copyright 2023, Wiley‐VCH. **b** Three-layer sandwich woven skin (TSW e-skin) capable of self-powered tactile sensing [149]. Copyright 2024, Elsevier. **c** A full fabric tactile sensor with multiple fabrics stacked alternately [150]. Copyright 2019, WILEY–VCH. **d** A sandwich structure flexible tactile sensor consisting of silver-coated velcro fabric and spacer fabric [151]. Copyright 2023, American Chemical Society. **e** Adaptive tactile interactive smart gloves [152]. Copyright 2024, Springer Nature. **f** The spiral expansion structure of the core sheath fibers used for tactile sensing fabrics [153]. Copyright 2019, Elsevier

Niu et al. proposed a full fabric biomimetic electronic skin with bimodal sensing based on intuition (fringing effect) and touch (iontronic effect), as shown in Fig. 11a [148]. These tactile sensors consisted of conductive fabric electrodes and an ionic liquid-poly(vinylidenefluoride-co-trifluoroethylene) fiber-TiO_2_ nanorod structure (IL-FNS) fabric dielectric layer, and they were interleaved with each other to form the double interlocked structure. In order to develop reliable and durable tactile sensors that can be applied to robots in extreme environments, Jin et al. developed a frictional electric three-layer braided electronic skin (TSW e-skin) based on the self-powering characteristics of triboelectric sensing devices (Fig. 11b) [149]. The prepared fabric electronic skin had excellent mechanical strength (~ 20 MPa), thermal stability (154.5 °C), long-lasting superhydrophobicity (> 150 °C), and corrosion resistance (pH 1–13), providing ideas for sensitive motion perception and tactile recognition of human and robot limbs in various extreme environments.

Piezoresistive devices based on changes in contact area for tactile sensing have been widely studied, but balancing high sensitivity and wide linear detection range has become a major challenge for researchers. The unique layered structure of the fabric allows stress to be evenly distributed between layers while increasing the contact area, further enhancing sensitivity and linear detection range. Based on the hierarchical structure and multilayer geometric shape of fabrics, Pyo et al. reported a highly sensitive and flexible resistive tactile sensor composed of carbon nanotubes (CNTs) and nickel-coated fabrics (Fig. 11c) [150]. The full fabric sensor exhibited significant pressure sensing performance and high sensitivity (26.13 kPa^−1^) over a wide pressure range (0.2–982 kPa). In addition, the changes in electrode spacing and contact area significantly affect the performance of capacitive sensors. To improve the deformation ability of the interlayer in multilayer fabrics, Su et al. developed a unique velcro tactile sensing fabric (Fig. 11d) [151]. Results showed that the sandwich frame of the fabric sensor significantly enhanced the compression deformation ability of the fabric layer, balancing the sensitivity, sensing range, and linearity of the device. The all-textile TS demonstrated outstanding sensitivity with a one-layer structure (0.036 kPa^−1^) over a pressure range of 0.2–5 kPa and retained a sensitivity of 0.002 kPa^−1^ in a four-layer structure over a wide pressure range of 0.2–110 kPa.

The impact of mechanical and chemical erosion or wear such as sweat and oil stains is a significant issue for fabric-based tactile sensors. Therefore, the environmental stability and mechanical robustness of the sensing fabric are undoubtedly important. Luo et al. proposed a wearable human–machine interface based on textile materials. The interface integrated tactile sensors and vibration tactile actuators, which was digitally designed and rapidly prepared (Fig. 11e) [152]. This not only promotes its application in the field of wearable devices, but also effectively expands the application scenarios of robots that integrate tactile sensing fabrics. In addition, the quantitative measurement and spatial resolution of tactile sensors are possibly affected by geometric deformation and interference from adjacent pixels. Deng et al. designed a spiral expansion structure of core sheath fibers for tactile sensing textiles (TST) to overcome the aforementioned challenges, which exhibited pressure sensing and spatial sensitivity independent of bending (Fig. 11f) [153]. The microstructure and conductive path in core sheath optical fibers maintained the same configuration under different bending stresses. Therefore, this TST presented identical pressure sensitivity under different radii of curvature.

### Other Bionic Structures

Currently, researchers have successfully commenced the replication of biological structures by employing a wide variety of materials, including diverse plant and animal organs [154, 155]. This methodology has been validated as an efficacious approach for constructing low-cost tactile sensors that possess high sensitivity and a broad response range.

**Fig. 12:**
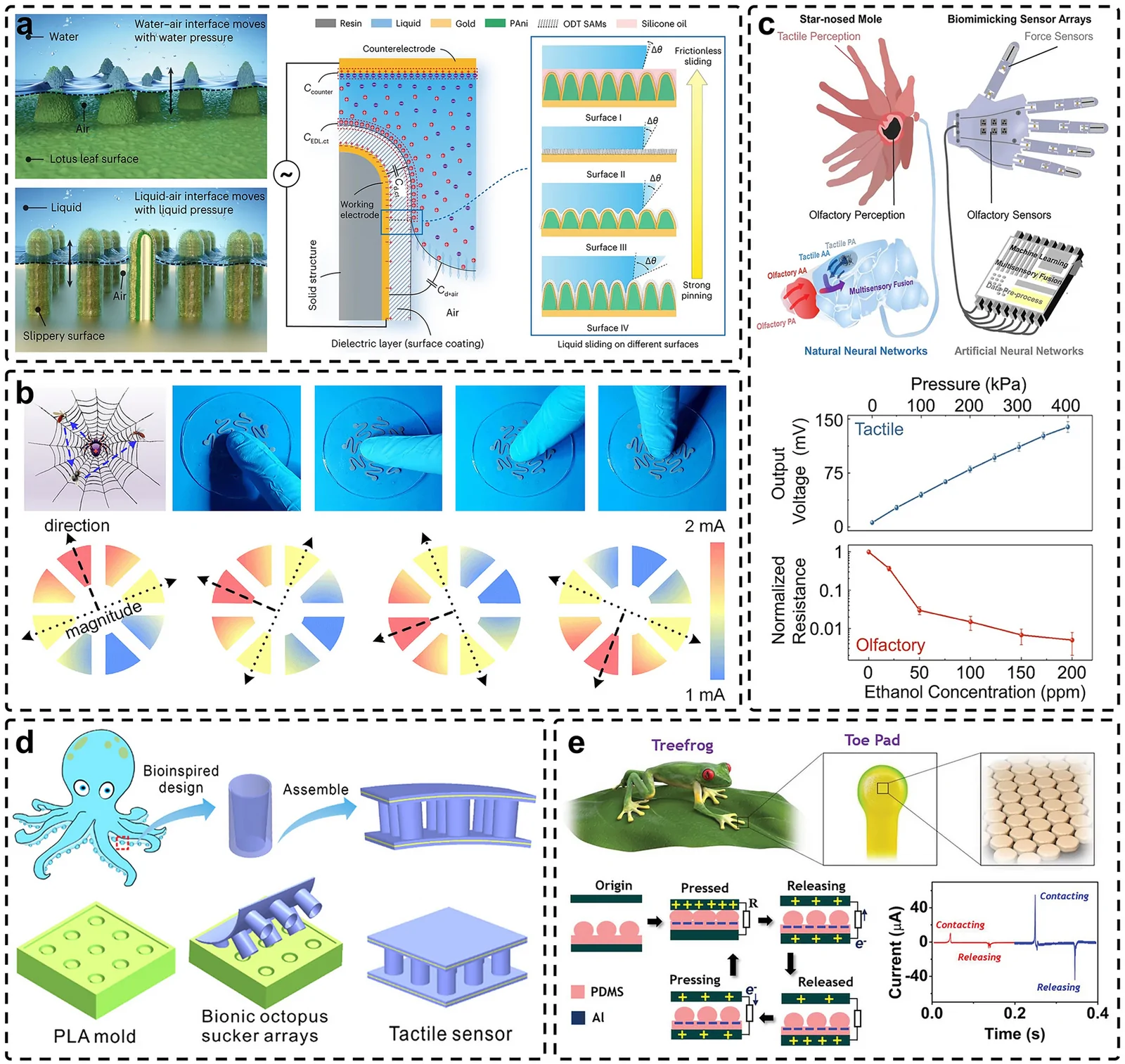
**a** Pressure sensors inspired by air retention on the surface of lotus leaves [156]. 2023, Springer Nature. **b** Spider web-like flexible tactile sensor [157]. Copyright 2021, American Chemical Society. **c** A star-nose-like tactile-olfactory bionic sensing array [158]. Copyright 2022, The Author(s). **d** Octopus sucker structure bionic tactile sensor [159]. Copyright 2022, American Chemical Society. **e** Tactile sensors inspired by the structure of treefrog toe pads [160]. Copyright 2019, WILEY–VCH

Inspired by the air retention phenomenon on the surface of submerged lotus leaves, Cheng et al. harnessed the pinning-free contact line motion within a multiscale structured solid–liquid-liquid–gas multiphasic system to develop a novel aero-elastic capacitive pressure sensor known as eAir, as shown in Fig. 12a [156]. Through the creation of a super-slippery interface and the construction of electrodes at both nano- and microscopic scales, they attained a nearly frictionless contact line motion and thereby realizing a nearly ideal pressure sensing performance.

In addition to the characteristics of plants, the functional structures of diverse animals have also been extensively mimicked. It is well known that spiders are capable of discerning the direction of their prey by detecting the disparities in the vibrations of their webs in various directions. Inspired by this biological process, Zhao et al. proposed and fabricated a flexible dual-parameter pressure-strain devices with a spider-web-like structure. Through comparing the responses of different outputs, the magnitude and direction of the external force can be concurrently monitored. The mechanism of this process is illustrated in Fig. 12b [157]. Of note is the fact that the core component of this device is the three-dimensionally structured pressure-sensitive layer.

**Fig. 13:**
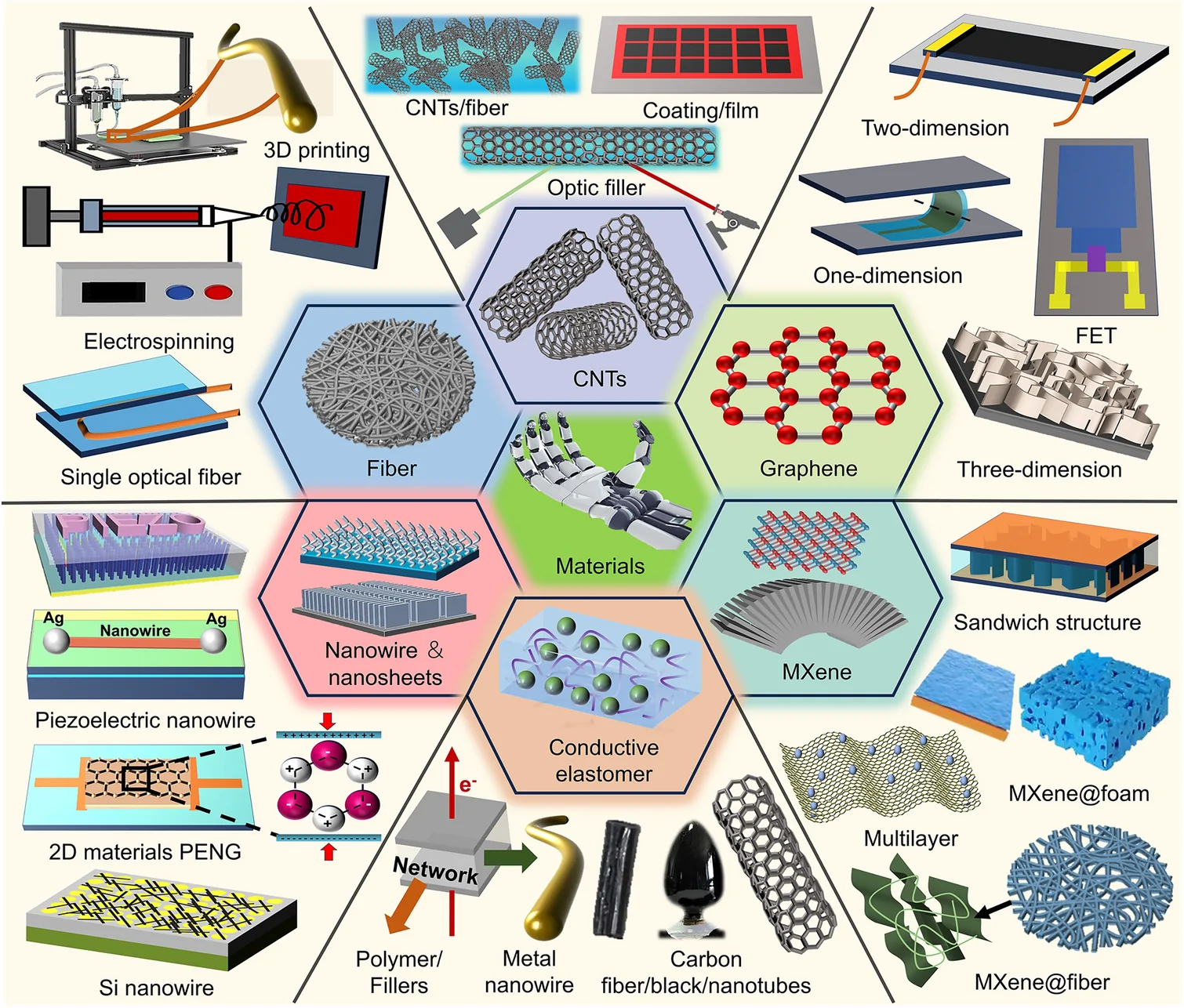
Functional materials applied in fabricating tactile sensors include graphene, carbon nanotubes, MXene, fibers, and other nanomaterials

The star-nosed mole is widely recognized as possessing the most powerful tactile perception capacity among mammals. This remarkable ability originates from the high-density tactile units that overlay the nose of the star-nosed mole. Tao and his team reported a star-nose-inspired tactile and olfactory sensing array that was mounted on a robotic hand. This array facilitated the real-time acquisition of the local topography, stiffness, and odor of an object when it was being touched, as depicted in Fig. 12c [158]. Such system incorporates a series of silicon-based force and gas sensors which possess high sensitivity and stability. Besides, Guo et al. proposed an arrayed tactile sensor, which increased the linear sensing range (8–500 kPa). The device was composed by integrating two conductive silver adhesive electrodes and a PDMS dielectric layer with an octopus sucker structure, as shown in Fig. 12d [159]. Inspired by the fact that the toe pads of tree frogs exhibit remarkable frictional capabilities, Oh et al. employed a customizable non-close-packed microbead array for the purpose of efficient dielectric triboelectric surface design, as shown in Fig. 12e [160]. By emulating the friction pads of tree frogs, they achieved a significant enhancement in the electrification performance and reliability of triboelectric nanogenerators (TENGs) (Fig. 13).

The three fundamental principles of bionic sensor design are morphological biomimicry, material biomimetics, and functional biomimicry. In recent years, skin-inspired, textile-inspired, and the aforementioned other bio-inspired structures represent the cutting-edge and hot topics in current flexible sensor research. They seek to address the limitations of original structures in aspects such as sensitivity, stretchability, comfort, multifunctional integration, and environmental adaptability. The impacts of distinct biological features on tactile sensor performance are compared in the Table 1.

**Table 1:** Relationships between several biomimetic structures and their biological counterparts

Biosystem	Extracted Feature	Sensor Implementation	Performance Impact	References
Human skin	Slow/fast adaptation receptors	Piezoresistive piezoelectric module integration	Dynamic and static stimulus perception	[144]
	Fingerprint	Ridged micropattern	Multimodal sensing	[147]
	Epidermis, dermis, and subcutaneous tissue	Stacking of triboelectric and capacitive layers	Improved material identification accuracy	[148]
Lotus leaves surface	Air retention phenomenon	Micro/nano electrode arrays	Excellent linearity (R^2^ = 0.99944)	[156]
Spider web	3D mesh structure	3D patterned pressure-sensitive layer	Direction perception of force	[157]
Star-nosed mole	High-density receptors	Silicon-based sensor arrays	Tactile and olfactory fusion	[158]
Octopus sucker	Array of micropillars with open apex	PDMS micropillar arrays	Increased linear sensing range (8–500 kPa)	[159]
Treefrog toe pads	Hexagonal micropillar array	Array of interfacial micropillar beads	Microstructure increases the contact friction limit	[160]

## Materials and Fabrications

The sensing performance of tactile sensors largely depends on the intrinsic properties of materials. Due to the inability of traditional rigid pressure sensor components to meet the requirements of working conditions such as bending or stretching, flexible devices have attracted more and more attentions. To achieve the optimal combination of mechanical and sensing performance, transforming rigid materials into nanomaterials or developing new functional materials becomes an effective method to conquer this problem. Here, we have summarized the commonly used functional materials for tactile sensors and the advantages and limitations of commonly used materials, which included a discussion on hybrid material systems.

### Graphene

Graphene has attracted much attention since its discovery and become one of the most influential nanomaterials. Graphene has a two-dimensional carbon atomic layer structure, which can be assembled into various types of macroscopic structures through different manufacturing methods to obtain electromechanical properties with application potentials [161–163]. Because of its high Young's modulus, ballistic transport of carriers, charge carrier behave mass less Dirac fermions and capability of modifying band gap with applying strain, it has become a favorite in the field of flexible electronics [164–168]. Different forms of graphene (including pure graphene, oxidized graphene, and reduced graphene oxide) have been reported to be applied in the construction of tactile sensors.

**Fig. 14:**
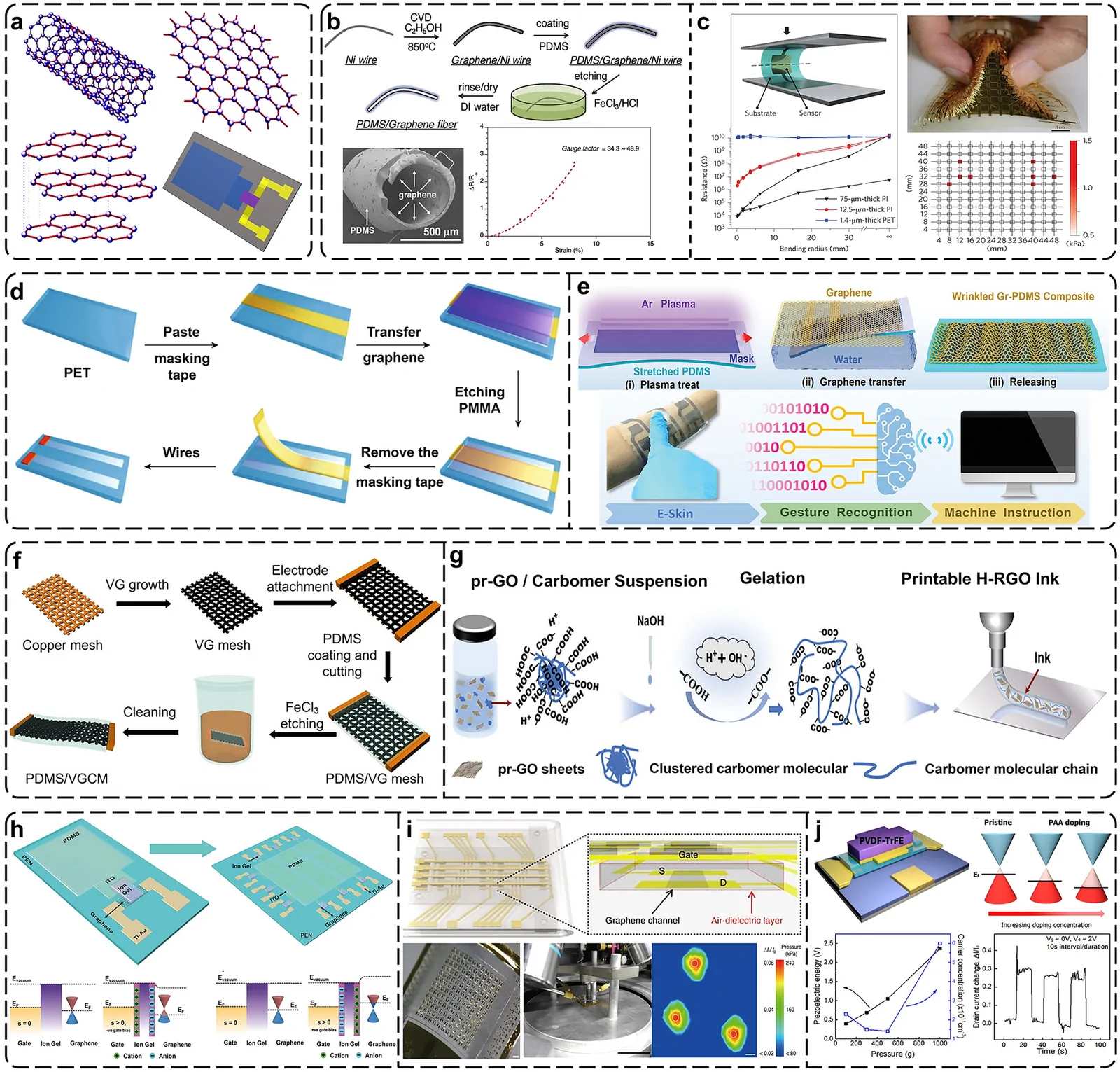
**a** Graphene materials with different nanoscale; **b** strain sensor based on hollow tubing graphene fibers (TGFs) with dimethylpolysiloxane (PDMS) coating [169]. Copyright 2017, Elsevier. **c** Bending insensitive pressure sensor[170]. Copyright 2016, Springer Nature. **d** Tactile sensors based on graphene film/PET [171]. Copyright 2018, Springer Science Business Media. **e** Conformal wrinkle graphene sensors and human–computer interaction applications [172]. Copyright 2021, Wiley‐VCH. **f** Vertical graphene canal mesh for strain sensing [173]. Copyright 2022, American Chemical Society. **g** Preparation of H-RGO inks and haptic sensing applications of devices [174]. Copyright 2022, Wiley‐VCH. **h** Graphene triboelectric tactile sensor array [175]. Copyright 2016, WILEY–VCH. **i** Pressure-sensitive graphene FETs with an air dielectric layer [176]. Copyright 2017, The Author(s). **j** Graphene-silicon Schottky diode tactile sensors [177]. Copyright 2019, WILEY–VCH

The high aspect ratio of one-dimensional materials greatly improves the transmission efficiency of stimuli whereas the nature of graphene two-dimensional materials makes the preparation of one-dimensional structure difficult. Nakamura et al. used nickel wires as templates to obtain one-dimensional hollow tube CVD graphene fibers (TGF), which improved the charge conduction efficiency of graphene [169]. The preparation process is shown in Fig. 14b. Electrospinning technology is a kind of spray film making process under strong electric field, which can effectively control the spatial arrangement of fiber materials. In the work proposed by Lee et al., the prepared composite nanofibers of carbon nanotubes and graphene had one-dimensional response ability and were insensitive to interference signals such as bending [170]. The sensor array based on this material had the ability to measure the vertical pressure distribution of a soft object in three-dimensional space, as shown in Fig. 14c.

With the deepening of the research in the field of electronic skin, intrinsic tensile properties have become the focus of researchers. This is due to the inevitable severe mechanical deformation in the detection process, which leads to the decline and even damage of the sensing performance of the device. Xu et al. deposited two groups of unconnected graphene film materials on PET film by CVD, realizing sensitive tactile sensing. The device preparation process is shown in Fig. 14d. The prepared device showed high sensitivity and light transmittance [171]. In addition, the electrical properties of graphene can be effectively improved by doping, defect engineering, stress engineering and other treatments. Jiang et al. used the mismatched deformation of the substrate after plasma treatment to construct wrinkles and prepared a thin shape preserving wrinkled graphene elastomer composite with intrinsic tensile nanoscale [172]. This conformal wrinkle structure provided the device with strong tensile resistance (100% strain) and strain insensitive characteristics, as shown in Fig. 14e. Compared with two-dimensional graphene film, vertical graphene has the advantages of three-dimensional scale sensing, showing excellent strain sensing performance. Ma et al. developed a vertical graphene pipe network (VGCM) for ultra-low strain sensing [173]. The vertical graphene pipe network played a role of stress concentration and realizes the induction of 0.1‰ strain. The manufacturing process of strain sensor based on VGCM is shown in Fig. 14f.

Utilizing 3D-printing technology, it becomes possible to construct three-dimensional graphene composites and integrate microstructure units with varying pressure-sensitive characteristics, mirroring the dual mechanoreceptors found in human skin. Liang et al. designed a graphene direct-writing ink formulated with carbomer hydrogel, enabling direct-writing printing with a low solid content [174]. Additionally, they crafted laminated graphene with diverse structures, which were subsequently integrated into piezoresistive sensor arrays, as depicted in Fig. 14g.

Compared to piezoresistive and capacitive sensors, field effect transistor (FET) pressure sensors possess distinct advantages such as excellent signal amplification, high array uniformity, high spatial contrast, and ease of integration with circuits. Modulating carrier transport in the semiconductor channel through the coupling of triboelectric potential with FET represents a novel modulation approach. Khan et al. developed a graphene-based triboelectric tactile sensor, utilizing a single-electrode mode triboelectric nanogenerator (S-TENG) and a GFET in coplanar coupling, as illustrated in Fig. 14h [175]. However, due to the inability of solid dielectrics in the dielectric layer to respond sensitively to external forces, Shin et al. successfully devised an unconventional method to fabricate a fully integrated active matrix array of pressure-sensitive top-gate graphene transistors featuring an air dielectric layer [176]. The device structure is illustrated in Fig. 14i. In addition, the piezoelectric potential can also serve as a gate modulation voltage. Chen et al. proposed a piezoelectric graphene artificial sensory synapse leveraging a piezoelectric potential-powered/modulated bilayer graphene transistor (Fig. 14j). In contrast to traditional artificial sensory neurons, the piezoelectric potential readily powered the synaptic device, offering a novel direction for the development of low-power artificial afferent nerves equipped with high-efficiency perception and neuromorphic computing capabilities [177].

### Mxene

Mxene, as an emerging two-dimensional layered material, exhibits excellent electrical, mechanical, and biocompatibility properties similar to those of graphene [178]. Meanwhile, MXene-based composites have proven to be promising candidates for flexible tactile sensors, owing to their exceptional tensile properties and metallic conductivity [179, 180]. The devices application research of MXene primarily focuses on electrochemical [181], biological [182], humidity [183, 184], optical [185–187], and other penetrating sensors. The classification methods of tactile sensors based on piezoresistance, capacitance, piezoelectricity, and triboelectricity have been widely recognized and reviewed. Here, we aim to reclassify and discuss the structural characteristics of MXene-based tactile sensors, with the hope of generating new insights for device fabrication.

**Fig. 15:**
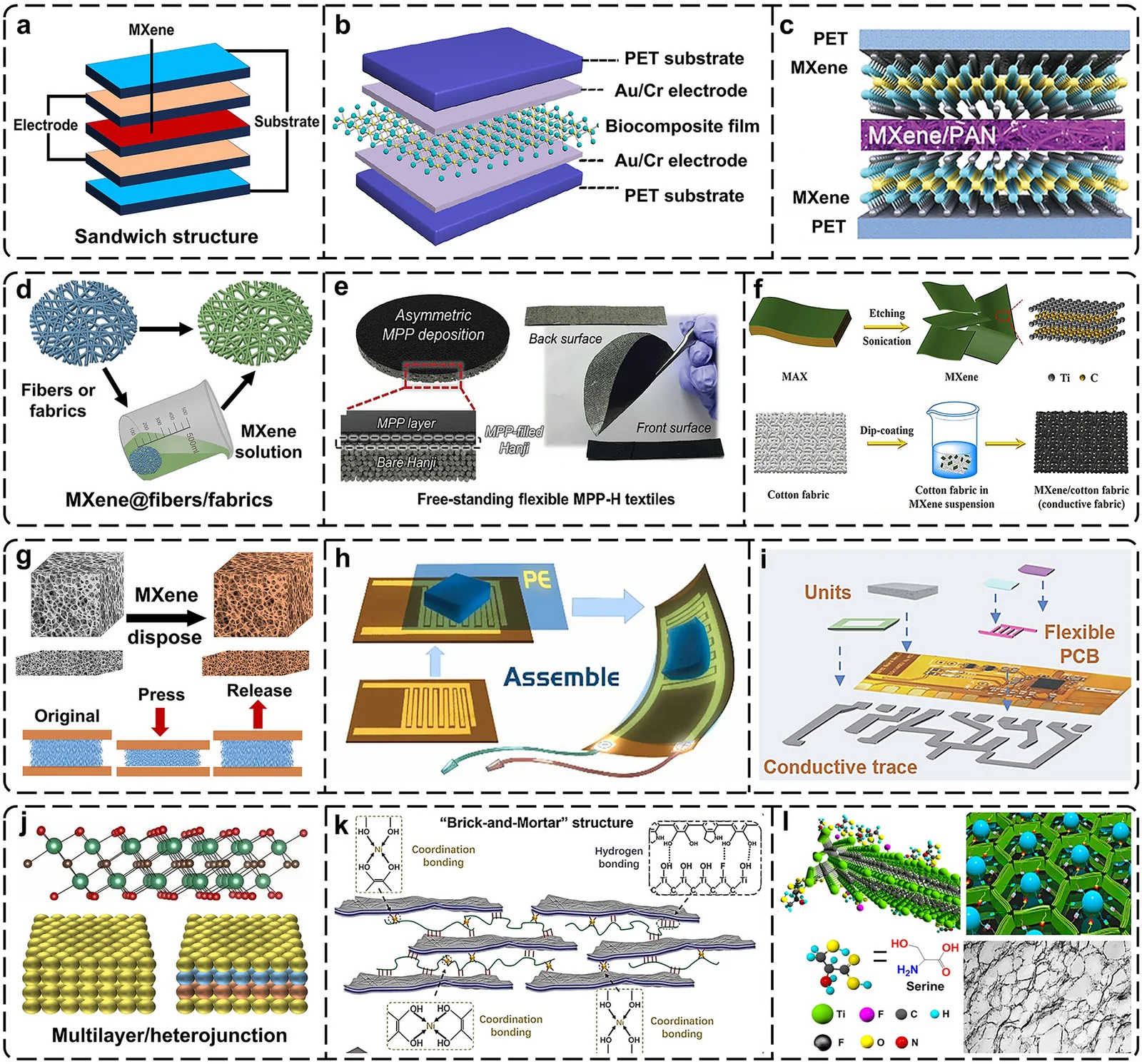
**a** Sandwich structure devices with MXene as the functional layer; **b** schematic diagram of SF/Mxene composite membrane flexible pressure sensor and array [188]. Copyright 2020, Elsevier. **c** Manufacturing process of flexible pressure sensor based on MXene/PAN [189]. Copyright 2021, Wiley‐VCH. **d** MXene/fiber composite structural functional layer; e MXene/PVA/PAA Hanji (MPP-H) multifunctional intelligent textile [190]. Copyright 2024, by Elsevier. **f** MXene/cotton fabric device [182]. Copyright 2020, Elsevier. **g** MXene/fiber functional layer. **h** Fabrication of the pure Hygel-based multifunctional sensing system as a robotic skin [192]. Copyright 2022, Elsevier. **i** Water-modulated super-attribute gel (hygel) electronic skin [193]. Copyright 2023, American Chemical Society. **j** Multilayer/heterojunction devices. **k** Brick-and-mortar structure [194]. Copyright 2019, American Chemical Society. **l** Serine-modified MXenes network [195]. Copyright 2024, American Chemical Society

The multilayer structure of sandwich-structured devices can effectively transmit electrons, evenly conduct current from the external circuit, reduce resistance loss, and thereby enhance the electrical efficiency of the device. Simultaneously, the multilayer stacking structure effectively disperses mechanical stress, preventing the device from fracturing or being damaged when subjected to bending, vibration, and other external forces. In recent years, with the in-depth study of MXene material properties, sandwich devices featuring MXene as the functional layer have garnered widespread attention from researchers. However, due to the inherent two-dimensional nature of MXene, the disadvantage of low aspect ratio and inability to form continuous macroscopic structures is observed. Wang et al. self-assembled the original Ti₃C₂Tₓ MXene nanosheets using silk fibroin (SF) as a bridging agent, transforming them into continuous wavy layered macroscopic structures as the functional layers for flexible sensing devices [188]. The composite film functional layer showed ideal flexibility and a low elastic modulus (1.22 MPa), high sensitivity (25.5 kPa^−1^), a low detection limit (9.8 Pa), and strong mechanical durability (over 3500 cycles). In addition, the pressure sensor assembled with a flexible pressure sensor array could be used to simultaneously map the spatial distribution and position of touchable pressure. The device array structure is shown in Fig. 15b. In additi0n, MXene is also regarded as a good candidate for flexible electrodes because of its outstanding conductivity. Wang et al. developed an MXene/PAN (polyacrylonitrile) composite thin film-based flexible pressure sensor with a uniform Ti₃C₂Tₓ MXene electrode and demonstrated excellent device performance, featuring a high sensitivity of 104.0 kPa^−1^, a fast response/recovery time of 30/20 ms, and a low detection limit of 1.5 Pa. The structure of MXene/PAN composite film is shown in Fig. 15c [189].

Mxene nanosheets hold great potential for the development of strain and pressure sensors based on conductive textiles owing to their high hydrophilicity and the ease with which they can be integrated with textile materials. The straightforward preparation procedure of the composite functional layer is illustrated in Fig. 15d. In order to further enhance the durability of the device, Kim et al. utilized crosslinked MXene (Ti_3_C_2_T_x_), poly (vinyl alcohol) (PVA), and poly (acrylic acid) (PAA), filtering them onto traditional Korean paper, Hanji, through vacuum filtration (as shown in Fig. 15e) [190]. The electronic fabric materials possessing flame retardancy, rapid Joule heating capabilities as well as durability and washability were successfully obtained. Zheng et al. successfully fabricated a piezoresistive pressure sensor based on wearable MXene/cotton fabric (MCF) by means of a simple and cost-effective dip coating process, as shown in Fig. 15f [191]. In these cases, the adhesion of MXene was achieved through the combination of the hydroxyl groups on the surface of the cotton fabric and the functional groups of MXene. When adjusting the concentration of the MXene dispersion within the range of 0.1–0.4 mg mL^−1^, the resistance of the device underwent a significant change, ranging from 1946 to 168 Ω.

Owing to the natural interconnection network within the sponge structure, the continuity of its internal microstructure will be markedly altered under the influence of external forces. Analogous to the preparation process of functional layers based on fabric materials, researchers frequently prepare them through a two-phase filling method. Cheng et al. filled the PU (polyurethane) sponge with MXene/rgo aerogel clusters (Fig. 15h) [192]. The support structure based on the sponge material effectively addressed the issue of the aerogel's susceptibility to collapse during the process of compression and release. The pressure sensor based on the MGP-sponge exhibited high sensitivity (224 kPa^−1^), and could detect ultralight objects (a grain of rice, 21 mg). In order to more closely mimic human tactile sensations, Duan et al. put forward a water-modulated bionic super-attribute gel (hygel) electronic skin with a reversible gel-solid transition, as shown in Fig. 15i [193]. Mxene nanosheets were chosen as the conductive fillers within the hygel system to construct a conductive percolation network. Through mechanical cutting and double assembly, a multifunctional sensor system based on hygel was fabricated, which incorporated a flexible printed circuit board (PCB) and multifunctional sensors. This system was capable of responding to temperature, humidity, contact, pressure, and strain, and it exhibited high accuracy.

In the working process of sensor devices based on nanosheet materials, there is an inescapable phenomenon of strain-induced crack propagation, which gives rise to a conflict between sensitivity and extensibility. Researchers hope to solve this problem by referring to the synergistic interaction between layered micro- or nanostructures and ionic bonds in nature. Liang et al. constructed a bioinspired Ti_3_C_2_T_x_-AgNW-PDA/Ni^2+^ strain sensor based on this concept, as shown in Fig. 15k [194]. The wearable strain sensors featuring different geometric shapes were manufactured by directly screen-printing the nanocomposite gel onto the stretchable polyurethane substrate, followed by drying it for 3–5 min. At the same time, the self-healing ability represents another approach to addressing the issue of interface damage. Guo et al. reported a self-healing flexible sensor in which a serine-modified MXenes network was constructed within a rubber-based supramolecular elastomer (Fig. 15l) [195]. The MXene nanosheets established hydrogen bonding interfaces through intermolecular interactions with the elastic molecular chains, thereby enabling the nanostructured Ti₃C₂ MXenes/rubber-based supramolecular elastomer (NMSE) to regain its original properties after complete fracture.

### CNTs

Carbon nanotubes (CNTs) are a type of one-dimensional quantum material with a special structure. Their radial size is on the order of nanometers, while their axial size is on the order of micrometers. Besides, they possess excellent intrinsically mechanical properties. Specifically, their elastic modulus and tensile strength are claimed as high as 1 TPa and 100 GPa, respectively, and their elongation at break ranges from 15% to 30% [196–198]. Thus, they are ideal materials for the preparation of flexible sensors. CNTs are frequently utilized as fillers within elastomer films for the purpose of fulfilling the flexibility requirements and attaining the electromechanical properties of the working layer in flexible sensor devices. Simultaneously, along with the in-depth exploration of fabric fiber-based materials, they are being utilized more and more frequently to fabricate functional films through combination with fiber materials. Moreover, on account of their outstanding optical properties, such as optical polarization, optical correlation, and luminescence performance [199, 200], among others, they are also extensively employed in the realm of optical tactile sensing materials.

**Fig. 16:**
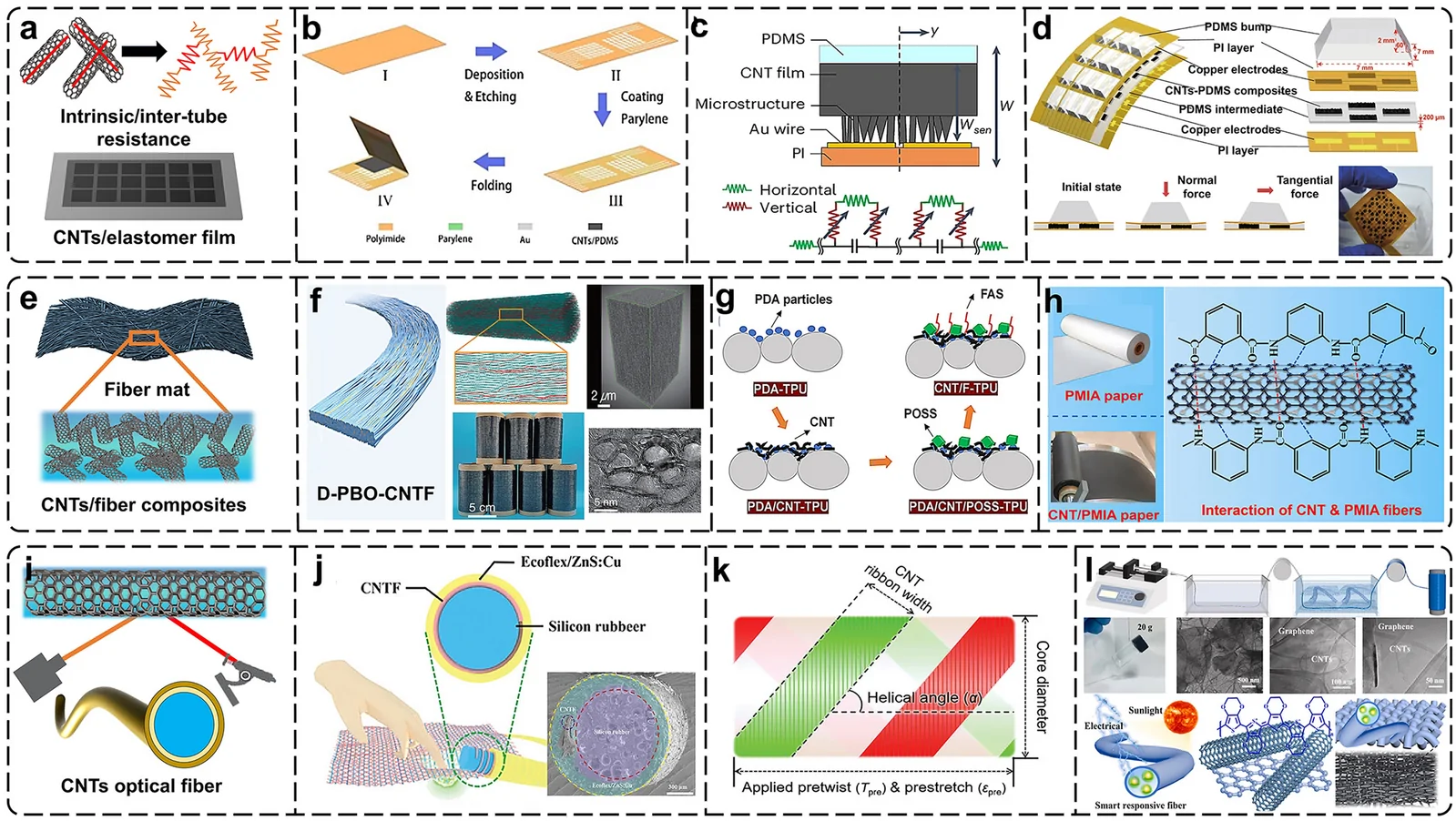
**a** Devices based on CNTs/elastomer film; **b** schematic diagram of the manufacturing process of CNTs/PDMS tactile sensor arrays [197]. Copyright 2017, Elsevier. **c** CNTs/PDMS-based tactile sensors with randomly distributed microcone structures [201]. Copyright 2024, Springer Nature. **d** CNTs/PDMS tactile sensors for three-dimensional force detection[202]. Copyright 2019, The Author(s). **e** CNTs/fiber composites; **f** flexible piezoresistive pressure sensor based on carbon nanotubes (CNTs)/carbon cloth (CC) [203]. Copyright 2022, Wiley–VCH. **g** Schematic diagram of the manufacturing process of superhydrophobic CNT/F-TPU fiber mats [204]. Copyright 2023, American Chemical Society. **h** Schematic diagram of the preparation process of CNT/PMIA electrothermal paper [205]. Copyright 2021, American Chemical Society. i CNTs/optical fiber composites; **j** schematic diagrams of the principle and cross-sectional SEM images of MLTENGF [206]. Copyright 2024, The Author(s). **k** The single-fiber double helix buckle-type optical fiber [207]. Copyright 2024, Wiley–VCH. **l** Multistimuli-responsive phase change optical fibers [208]. Copyright 2023, Elsevier

The microstructure of CNTs-based devices has a great influence on their electronic properties. Compared with random CNT networks, aligned CNT arrays exhibit enhanced conductivity and thermal conductivity in parallel directions. In the study of Shi et al., the cross-stacked CNT films were chemically hybridized with graphene by chemical vapor deposition (CVD), which increased the strain sensitivity of CNT films by 5–10 times, as shown in Fig. 16b [197]. Likewise, on the basis of a flexible silicon-based material substrate, Yi and his colleagues developed a tactile oral pad equipped with a touch sensor array, which was fabricated from a composite material consisting of CNTs and silica gel (Fig. 16c) [201]. CNTs were also employed as conductive nanofillers within the silicone elastomer matrix, and concurrently, randomly distributed spiky microstructures were introduced with the aim of enhancing the sensing performance. Besides the sensing performance of the functional layer, the three-dimensional structure of the device array also has a considerable impact on the sensing performance. Liu et al. showcased a 4 × 4 flexible tactile electronic skin sensor via fabricating CNTs/PDMS nanocomposite for the purpose of detecting three-dimensional contact forces [202]. The structure of the device and its cross sections in the initial state, when subjected to normal force, and when subjected to tangential force are shown in Fig. 16d.

CNTs are further deemed an effective filler for building functional fiber materials. In order to further simulate the human tactile perception range, Chen et al. proposed an interlocking structure Ni@Carbon as an intelligent tactile sensor. CNT arrays were used for ultra-sensitive physiological signal detection and real-time monitoring. The preparation process is shown in Fig. 16f. The CNT arrays are vertically grown and carbon fibers are woven into carbon cloth (CC). When pressure is applied to the sensor, the CNT array and the warp and weft carbon fibers show delamination deformation, which makes the pressure sensor have ultra-high sensitivity (~ 120 kPa^−1^) and wide pressure range (~ 0.15–10 kPa) [203].

Given the variable working environments of tactile sensor devices, such as humid, acidic, or alkaline conditions, the development of sensing materials that can work efficiently in different environments has drawn extensive attentions. Meng et al. proposed a simple layer-by-layer self-assembly method to prepare superhydrophobic stretchable conductive polymer nanofiber composites, which possess high chemical and mechanical stability, as shown in Fig. 16g [204]. Furthermore, paper-based materials have drawn extensive attention on account of their features such as being lightweight, highly pliable, and easily attainable. Yang and his colleagues came up with a lightweight, sturdy, tough, and flexible electrothermal paper that is based on CNT/PMIA (poly-p-phenylene terephthamide) fibers, as depicted in Fig. 16h [205]. The PMIA fiber network acts as the framework, and meanwhile, CNTs, functioning as highly conductive fillers, are co-refined together with PMIA fibers so as to accomplish the uniform dispersion of CNTs within the fibers.

Flexible dual-mode sensing optical fibers can perceive mechanical stimuli and convert them into digital visual signals. Zhang et al. built a stretchable self-powered mechanoluminescent triboelectric nanogenerator fiber (MLTENGF) based on lightweight carbon nanotube fibers [206]. The device had high stability and realized non-contact sensing. MLTENGF features a helical CNTF assembled in a sandwich structure, with its core of silicone rubber foam round bars and the outer layer made of silicone rubber composites (Fig. 16j). During the assembly process of optical fiber materials in devices, issues related to the interconnection of the anode and cathode as well as the interface contact were likely to occur. To tackle this problem, Son and his colleagues put forward a single-fiber system (Fig. 16k) [207]. Furthermore, they developed a highly stretchable multiresponsive phase change intelligent fiber by means of wet spinning [208]. This fiber featured a dual network composed of carbon nanotubes/PU and PEDOT:PSS, showing high sensitivity to a variety of external stimuli such as mechanical, electrical, thermal, and optical ones. The working mechanism of the composite fiber is illustrated in Fig. 16l.

### Conductive Elastomer

Conductive elastomers fall into a distinctive category of materials, featuring two prominent properties, namely conductivity and elasticity. Generally, they are fabricated using an elastomer as the base material. Typical elastomer bases include rubber, polyurethane, and the like. Based on this, a variety of conductive fillers like carbon nanotubes, metal nanoparticles, and conductive polymers are incorporated into it. When this material is exposed to external forces, it is capable of undergoing elastic deformation and, simultaneously, retaining a certain degree of conductive ability throughout the deformation process. Precisely due to these characteristics, conductive elastomers demonstrate unique application advantages within the realm of tactile sensors.

**Fig. 17:**
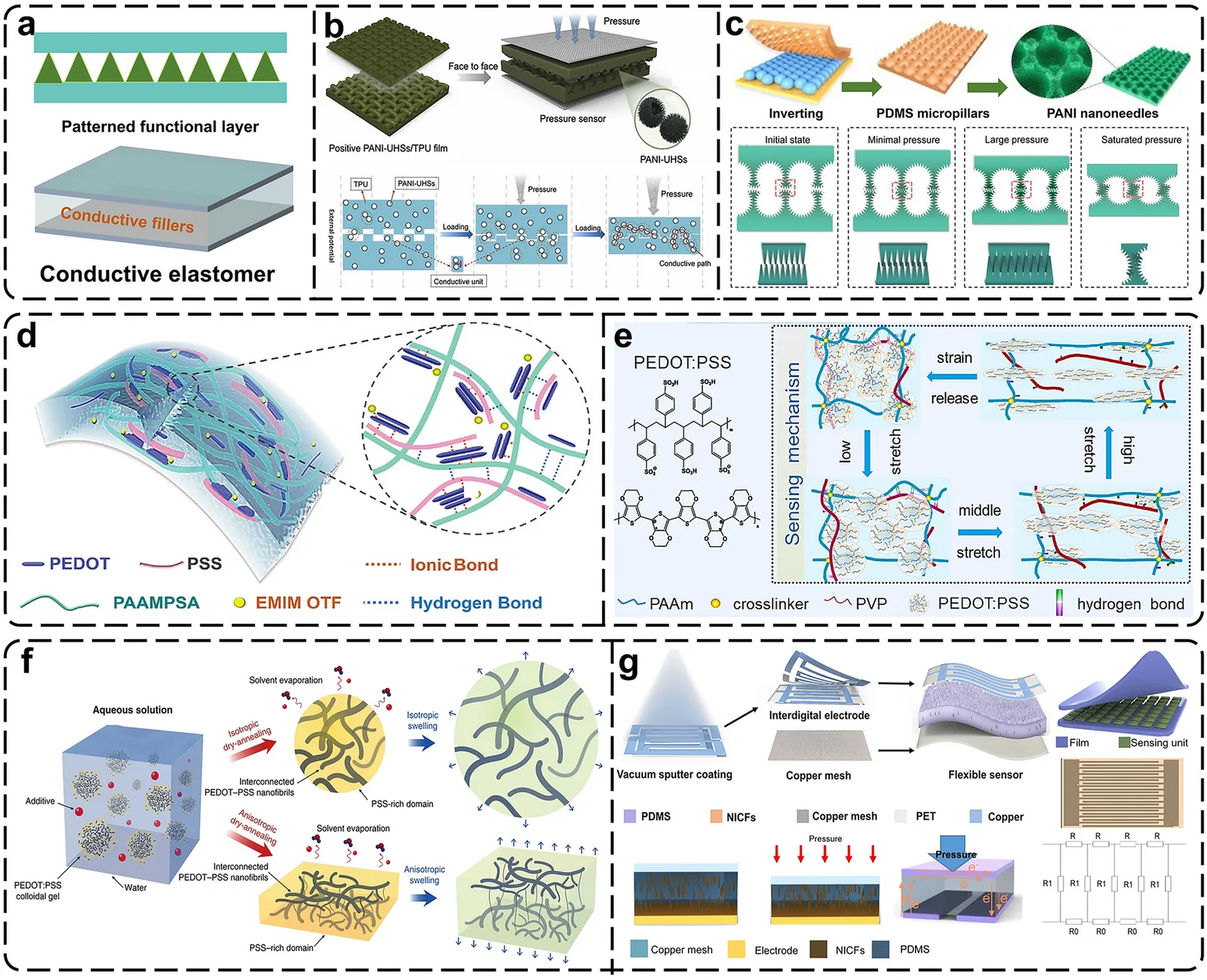
**a** Conductive polymer tactile sensing functional layers; **b** flexible piezoresistive sensors based on binary spike/sea urchin-like nanoparticle composite films [209]. Copyright 2022, Elsevier. **c** Hierarchical structure of PDMS micropillars decorated with PANI nanoneedles [210]. Copyright 2022, Elsevier. **d** Schematic diagram of the PEDOT:PSS/PAAMPSA/IL composite material [214]. Copyright 2022, Wiley‐VCH. **e** Schematic illustration of the preparation process and reversible stretching mechanism of SCH [215]. Copyright 2024, Elsevier. **f** Flexible tactile sensors coupled with pyramid and dome microstructures fabricated based on SCCF materials [216]. Copyright 2019, Springer Nature. **g** Schematic diagram of the manufacturing process of MAPS [217]. Copyright 2022, American Chemical Society

There are two significant aspects that demand consideration in this research, specifically, the conductive electrodes of the sensor and the deformation behavior of the elastomer. Researchers frequently resort to patterning strategies with the aim of enhancing the sensitivity and selectivity of sensor devices and bolstering the signal response. Huang and his colleagues put forward a flexible piezoresistive sensor founded on a micro/nanohierarchical interlocking structure, which was inspired by the intricate interlocking structures found in nature and incorporated the quantum tunneling effect that exists between the sharp structures of nanoparticles (as depicted in Fig. 17b) [209]. An elastomer film featuring an interwoven mesh structure was developed by utilizing a mixture of conductive particles and a thermoplastic polyurethane (TPU) substrate with a hollow sea urchin-like surface characteristic. In a similar vein, Li et al. constructed a hierarchical structure consisting of polydimethylsiloxane (PDMS) micropillars that were adorned with polyaniline (PANI) nanoneedles, as shown in Fig. 17c [210]. The primary interlocking of these nanoneedles, along with the secondary interlocking of the micropillars when subjected to high pressure, brought about high sensitivity (with a value of 258.7 kPa^−1^), a low detection limit (as low as 0.68 Pa), a rapid response time (merely 30 ms), and outstanding cyclic stability (capable of maintaining performance over 10,000 cycles).

PEDOT:PSS, which is the abbreviation for poly (3,4-ethylenedioxythiophene): polystyrene sulfonate, is an organic polymer composite boasting excellent conductive properties. The well-structured conjugation of PEDOT grants it a relatively high level of conductivity. Meanwhile, PSS, being a water-soluble polymer, plays the role of dispersing and stabilizing PEDOT. This allows the composite to be conveniently processed in the form of a solution and also endows it with favorable film-forming characteristics [211–213]. Despite the fact that remarkable progress has been achieved in the research and development of stretchable and self-healing conductive materials, it still poses a significant challenge to simultaneously preserve and completely restore these functions both before and after the healing process. Liang and his colleagues reported a highly stretchable and autonomous self-healing conductive film, which was composed of a conductive polymer PEDOT:PSS and a soft polymer (poly(2-acrylamido-2-methyl-1-propanesulfonic acid), abbreviated as PAAMPSA) [214]. The preparation process is illustrated in Fig. 17d. This polymer composite film demonstrated a favorable pressure sensitivity of 164.5 kPa^−1^, an extremely fast response time of 19 ms, and outstanding durability throughout more than 1500 consecutive presses.

In order to strike a balance between the high filler density, which is essential for attaining high conductivity of the film, and its stretchability, Li et al. put forward a 3D microprinted hydrogel tactile sensing device. The cross-linking process of the materials is shown in Fig. 17e [215]. Through customizing the density of both chemical and physical bonds, an equalization between high stretchability and high sensitivity was successfully accomplished. This sensor was cleverly fabricated by simultaneously manipulating the coupling of hydrogen and covalent bonds within conductive polymer nanoparticles, with particular emphasis on PEDOT:PSS. The resultant sensor offers an extremely wide detection range of 1500% along with a high gauge factor (GF) of 16.6. In a similar vein, with PEDOT:PSS being used as the raw material, Lu et al. designed the interconnection network of PEDOT:PSS nanofibers by a simple method and produced high-performance pure PEDOT:PSS hydrogel, as shown in Fig. 17f. The volatile additive dimethyl sulfoxide (DMSO) was mixed into PEDOT:PSS aqueous solution and then subjected to controlled dry annealing and rehydration [216]. The developed hydrogels demonstrated exceptional bioelectronic compatibility, featuring: high conductivity (up to 40 S cm^−1^ in aqueous media), significant stretchability (> 35% strain), low modulus (~ 2 MPa), robust mechanical/electrochemical stability, and tunable isotropic/anisotropic swelling behavior under physiological conditions.

Moreover, the orientation of the fillers can be regulated under the impetus of an external field, thus enabling the acquisition of elastomeric film materials in which the fillers are arranged in a directional pattern. Yang et al. showcased an ultra-high-sensitivity tactile sensor realized by means of a magnetically aligned conductive composite material (Fig. 17g) [217]. This sensor was assembled through the process of directionally embedding nickel-plated carbon fibers (NICF) into a PDMS substrate within the context of a magnetic field. Simultaneously, with the aid of sandpaper, a surface spiny structure was fabricated. The MAPS that was consequently obtained demonstrated an exceptional pressure sensitivity of 15, 525 kPa^−1^.

### Other Functional Materials

**Fig. 18:**
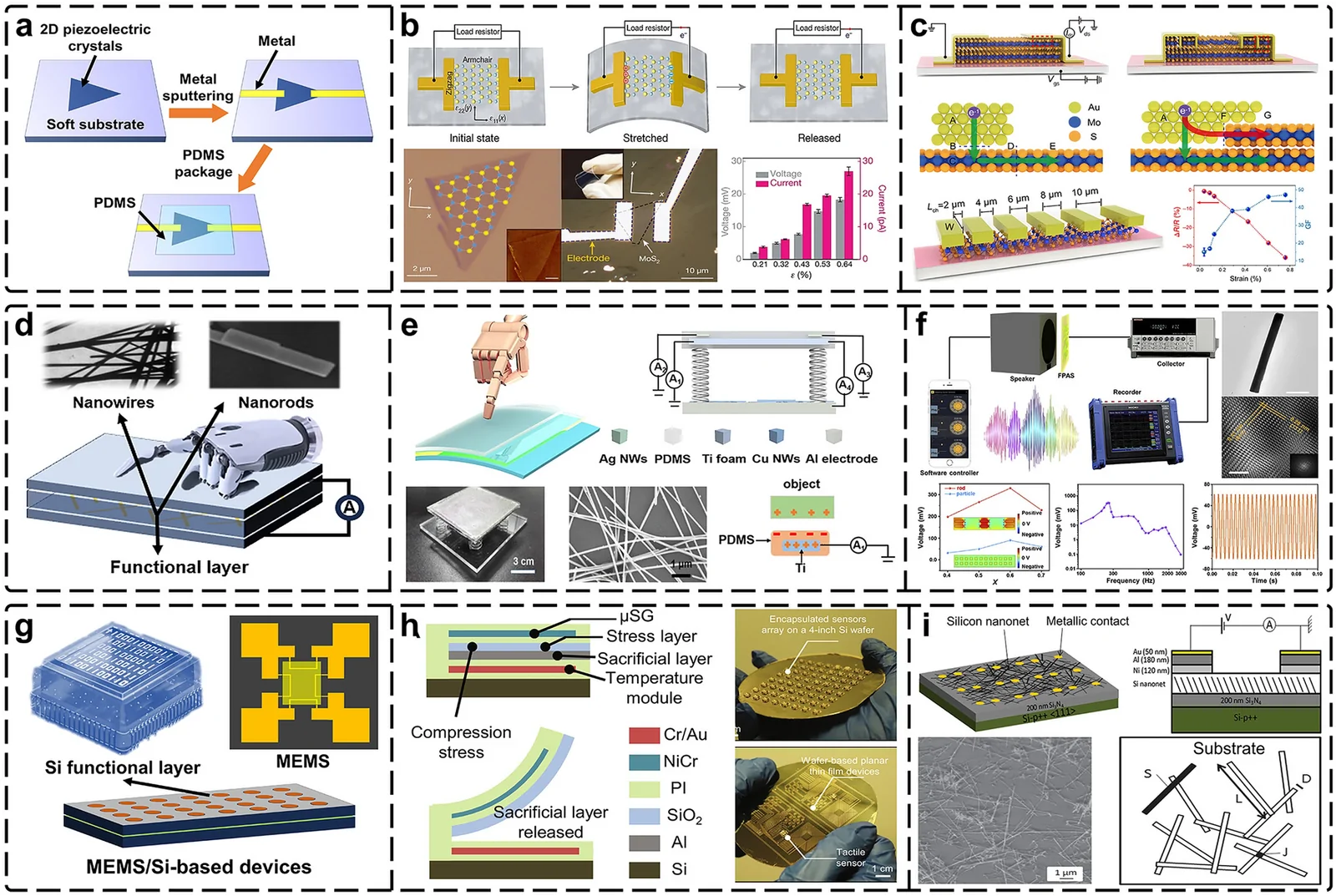
**a** The common preparation processes of two-dimensional material sensor devices; **b** Operational mechanism of single-layer MoS₂ devices [222]. Copyright 2014, Springer Nature. **c** Schematic diagram of the MoS2 FETs for contact resistance test [223]. Copyright 2024, The Author(s). **d** Sensor devices based on nanowire and nanorod materials; e The structure and working principle of triboelectric devices [224]. Copyright 2017, American Chemical Society. **f** Self-powered acoustic sensors based on KNN-based ceramic nanorods [225]. Copyright 2022, Elsevier. **g** MEMS/Si-based devices; **h** schematic diagram of the formation and integration of 3D μSG [231]. Copyright 2024, The American Association for the Advancement of Science. **i** Percolating silicon nanowire networks with a decline in their conductive performance over time [232]. Copyright 2006, Springer Nature

Aside from the commonly used materials previously mentioned, the sensing characteristics of nanowires and single-atom-layer crystals have become the focal points of intense research [218, 219]. Thanks to their high crystallinity and the capacity to endure substantial strains, they can be utilized in various aspects such as sensors, transducers, energy conversion, and electronics. For instance, silicon nanowires have a piezoresistive coefficient that exceeds that of traditional bulk materials [220, 221]. Meanwhile, with the gradual refinement of the theory of piezoelectricity and the development of piezoelectric devices, the sensing applications of 2D thin-layer materials have drawn more attention from researchers. The device structures crafted from 2D thin-layer materials are commonly prepared by depositing the 2D material layers as well as the output electrodes on a flexible substrate, as illustrated in Fig. 18a. Wang et al. carried out the first experimental study on the piezoelectric properties of two-dimensional molybdenum disulfide (MoS₂) and showed that the cyclic stretching and releasing of thin MoS₂ flakes with an odd number of atomic layers can produce oscillating piezoelectric voltage and current outputs (Fig. 18b) [222]. Upon this foundation, Luo et al. manufactured a FET (field-effect transistor) device for the purpose of testing contact resistance, employing two-dimensional MoS₂ materials [223]. The remarkable improvement in the electrical performance of the dendritic bilayer MoS₂ FET is due to the plentiful dendritic structures, which considerably enlarge the edge contact area between the gold electrodes and MoS₂. The device structure is depicted in Fig. 18c.

Nanowire and rodlike materials possess a host of advantages. In terms of physical properties, nanowires and nanorods have exceedingly small diameters, allowing them to possess a large specific surface area. Nanowires and rodlike materials are usually compounded with flexible films as fillers. Wang et al. developed a flexible, thin tactile sensor. The embedded silver nanowire electrodes precisely located the positions of small objects with a planar resolution of 2 mm (Fig. 18e) [224]. The coupling contact separation mode unit of the flexible sensor can linearly detect pressures within the range of 40 to 140 N. Taking KNN-based ceramic nanorods as the filling material, Li et al. prepared a self-powered acoustic sensor device, as shown in Fig. 18f [225]. The ceramic nanorods were dispersed as fillers in the PDMS matrix.

Microelectromechanical systems (MEMS) take advantage of the microfabrication techniques within the MEMS framework to integrate mechanical structures, sensing elements, and the like onto minuscule chips or devices. Among these components, the sensing element serves as the crucial core of the tactile sensor, being specifically designed to directly perceive external tactile stimuli [226–228]. When it comes to the preparation of sensing elements, silicon-based materials have been widely adopted. Among them, SiO₂ finds extensive applications in various fields including integrated circuit manufacturing, flat panel display manufacturing, optoelectronic device manufacturing, and LSI packaging material manufacturing owing to its outstanding insulating properties, high-temperature stability, mechanical strength, and chemical stability [229, 230]. Han et al. tackled the problems of multi-dimensional force decoupling and device integration constraints by introducing a set of flexible and modular tactile sensors fabricated using microelectromechanical systems (MEMS) technology [231]. The key to their success lies in incorporating a layer of silicon dioxide (SiO₂) with internal stress, which enables the construction of a 3D microstrain gauge (μSG) array for accurately measuring the amplitude and direction of mechanical stimuli. The structure of the device is depicted in Fig. 18h.

In comparison with bulk materials, the unusually large piezoresistive coefficient present in silicon nanowires has drawn extensive attention from researchers. Yang et al. reported that the longitudinal piezoresistive coefficient of 50-nm p-type silicon nanowires with a resistance of 10 Ω cm along the < 111 > direction is nearly 36 times that of bulk silicon (Fig. 18i) [232].

### Hybrid Material Systems

The application of hybrid materials in tactile sensors is driving a paradigm shift in technology, with its core value lying in multi-dimensional performance breakthroughs and enhanced scenario adaptability. From the perspective of intrinsic electrical properties, the synergistic conduction effects between high-carrier-mobility materials (carbon nanotubes, metallic nanowires) and graphene significantly can enhance sensing sensitivity. Regarding intrinsic mechanical properties, blending low-modulus elastomers with high-modulus materials (carbon nanotubes, graphene) enables tunable sensitivity and detection range, though interfacial bonding strength remains a critical challenge.

Hybrid material systems leverage three synergistic mechanisms: compositional complementarity ("conductive + elastic" components), structural cooperation ("micronano-hierarchical architectures"), and interface optimization ("chemical bonding") to potentially overcome the trilemma trade-off among sensitivity, stretchability, and stability in tactile sensors. Future efforts must prioritize resolving scalable manufacturing and dynamic reliability under mechanical fatigue. We herein summarize the merits and constraints of distinct materials for tactile sensors, along with the performance augmentation and prevailing technical challenges of hybrid material systems, as systematically summarized in Table 2.

**Table 2:** Analysis of the Advantages and Limitations of Tactile Sensor Materials

Materials	Advantages	Limitation
*Single Materials*		
CNTs	High flexibility and toughness High carrier mobility	Poor dispersubility Directional arrangement difficulty
Graphene	High electrical conductivity Mechanical fatigue resistance	Weak interfacial bonding Difficulty in scalable manufacturing
MXene	High specific surface area Excellent film-forming ability	Low mechanical strength High production costs
Conductive Elastomer	Intrinsically stretchable Tailorable substrates	Viscoelasticity-induced hysteresis Long-term load-induced creep
Fiber	Low-cost weaving technology Exceptional wearability	Poor durability Low spatial resolution
Nanowires	High carrier mobility Excellent compatibility with flexible substrates	Mechanical instability High production costs
*Hybrid Material Systems*		
Materials	Core Advantages	Technology challenges
Mxene/Graphene	Suppress interlayer slipping Collaborate on conductivity	Poor long-term stability
CNTs/Elastomer	Sensitivity and stretchability	Dispersion uniformity
Mxene/Nanowires	Nanowire-bridged MXene lamellae	Oxidation of MXene
Fiber/Elastomer	Multi-directional detection and durability	Interface delamination

## Performance-Oriented Design

### Sensitivity

The sensitivity of tactile sensor devices denotes the extent of their responsiveness to external tactile stimuli, such as pressure, force, and vibration. It serves as a vital metric for gauging how precisely and sensitively a tactile sensor can detect minute alterations in tactile sensations. The factors that exert an influence encompass aspects like material properties (including piezoelectric coefficient, electrical conductivity, and electromechanical coupling performance), microstructure design (such as micropores and micropillar surfaces) [233, 234], and the precision of manufacturing processes (photolithography, deposition, and 3D printing) [235–237]. The performance assessment of tactile sensor devices can be conducted from two perspectives: static testing and dynamic testing. During static testing, a constant pressure value is typically imposed on the sensor, and the resultant output electrical signals (like voltage, resistance, and capacitance) are measured. The sensitivity is then evaluated by analyzing the correlation between the output signals and the applied pressure. In contrast, during dynamic testing, the dynamic tactile stimuli encountered in real-world applications, such as vibration and impact, are simulated. Vibration signals with varying frequencies and amplitudes are generated by specific apparatus and applied to the sensor, and the dynamic response characteristics of the sensor, including response time and frequency response of the signals, are monitored. Different types of tactile sensors will have different calculation methods based on their sensing principles.

For tactile sensors that work based on the piezoresistive principle, the sensitivity calculation formula is as follows:

5.1.1 $$S_{R} = \frac{\Delta R}{{R_{0} \times \Delta P}}$$

For tactile sensors that operate on the basis of the capacitance principle, the sensitivity calculation formula is as follows:

5.1.2 $$S_{C} = \frac{\Delta C}{{C_{0} \times \Delta P}}$$

Among them, ${S}_{\mathrm{R}}$ and ${S}_{\mathrm{C}}$ respectively represent the sensitivities based on the changes of resistance and capacitance; ΔR and ΔCdenote the amounts of change in resistance and capacitance; ΔP is the amount of change in the applied pressure.

In tactile sensors based on piezoelectric materials, the sensitivity is determined by measuring the relationship between the amount of electric charges generated and the applied pressure:

5.1.3 $${S}_{\mathrm{Q}}=\frac{Q}{\Delta P}$$

Among them, ${S}_{\mathrm{Q}}$ represents the sensitivity based on the piezoelectric effect. Q is the polarized charge amount of the piezoelectric material, and ΔP is the amount of change in the applied pressure.

### Linearity

Linearity serves as an indicator that gauges the linear correlation between the output signal of a tactile sensor and the applied tactile stimuli (such as pressure, force, displacement). More precisely, it embodies the extent of proximity between the actual output characteristic curve of the sensor and the ideal straight line [238, 239]. Under ideal circumstances, the relationship between the output and the input manifests as a straight line. A high level of linearity implies the precision of the sensor in measuring the applied stimuli. Meanwhile, the linear output leads to more convenient data processing and simplification [240, 241]. Linearity is typically influenced by the following factors, encompassing the materials and structure of the sensor, the manufacturing process, as well as the testing environment [242–244]. Linearity is generally expressed in terms of relative error, and the formula is as follows:

$${\mathrm{Linearity}}\, = \,\left( {{\text{Maximum Deviation Value }}/{\text{ FullScaleOutput Value}}} \right)\, \times \,{1}00\%$$

Among them, the maximum deviation value denotes the greatest difference between the actual output curve and the fitted straight line throughout the entire measurement range. The full-scale output value represents the output value of the sensor under the stipulated maximum input stimulus.

### Hysteresis

The hysteresis phenomenon pertains to the disparity in output values that a sensor demonstrates during the process of increasing and decreasing its input and output values. This phenomenon originates from numerous factors. Specifically, the viscoelasticity of elastomeric materials renders it impossible for them to revert to their original state once the external force is withdrawn, thereby giving rise to the hysteresis phenomenon [245, 246]. Additionally, the internal design of the sensor, such as the electrodes and plate spacing in capacitive tactile sensors, can lead to an uneven distribution of the electric field when the input quantity varies, consequently causing the hysteresis phenomenon. The existence of the hysteresis phenomenon will considerably diminish the accuracy of the output results. Simultaneously, in control systems predicated on tactile sensing signals, it will bring about unstable outputs, thereby resulting in confusion within the control logic.

With regard to the improvement measures for the hysteresis phenomenon, they are primarily contemplated from the perspectives of material selection, structural enhancement, and the elevation of the manufacturing process. Selecting materials endowed with better elastic recovery properties empowers them to return to their initial state more expeditiously and accurately when subjected to and released from pressure, thereby mitigating the hysteresis [247]. A rational structural design is capable of reducing component friction, optimizing the layout of sensing elements, enabling the changes in the input quantity to be responded to more smoothly and curtailing the hysteresis. More precise manufacturing processes, along with enhanced dimensional accuracies and surface roughness control, can guarantee that all components of the sensor fit together flawlessly after assembly, thereby diminishing the hysteresis caused by manufacturing process errors.

### Drift

The so-called drift phenomenon of tactile sensors, specifically, refers to the circumstance where the output signal of the sensor varies over time in an undesirable manner, gradually departing from its initial state or reference value when there is no actual alteration in external tactile stimulation. During the actual functioning of the sensor, numerous factors can trigger the drift phenomenon [248, 249]. Among them, environmental factors exert a significant influence. For instance, once environmental conditions like temperature and humidity change, they might disrupt the output signal of the sensor, leading to its drift. Additionally, the impairment of the working stability of the materials utilized in the sensor can also serve as a cause for the drift phenomenon. To effectively preclude the occurrence of this drift phenomenon, researchers typically adopt a series of targeted measures during the design of the devices. For example, they frequently incorporate temperature sensors into the devices to fulfill the function of temperature compensation for the device signals, thus minimizing the risk of drift brought about by temperature fluctuations. Meanwhile, in terms of material selection, they are inclined to opt for materials with high stability and will also conduct surface protection treatment on these materials. Through such means, the occurrence of the drift phenomenon can be comprehensively averted to ensure that the tactile sensors can operate more stably and accurately.

### Response Time and Reliability

The time it takes for the output signal of a device to accurately mirror the stable state corresponding to an external stimulus or to attain the specified level of output change, starting from the moment the device receives the external stimulus, is referred to as the response time [250]. Typically, the response time is associated with the characteristics of the sensor's sensitive elements as well as the performance of the functional layer of the elastic body. An overly large or small elastic modulus will result in a slowdown of the transmission of tactile stimuli and excessive deformation of the elastic body. Devices relying on the piezoresistive effect require a certain period of time for the resistance change to stabilize. Meanwhile, capacitive-based sensor devices may encounter response delays due to the improper design of the capacitor plate materials [251]. Moreover, the amplification circuits, filtering, and noise reduction processing that are commonly present in device design will lengthen the signal processing time. The determination of the response time can be broadly categorized into two major types: step response testing and impulse response testing, which respectively correspond to the time needed for step and impulse signals to reach a stable state from the moment the stimulus is applied.

Specifically, the repeatability of a tactile sensor pertains to the capacity whereby, when the sensor undertakes multiple measurement operations on the identical tactile stimulus, the resultant output can either remain consistently the same or display an extremely high level of similarity. With regard to this crucial parameter, the focal aspects are mainly centered around two key elements: Firstly, it concerns the degree of consistency attained during the manufacturing process of the sensitive components within the sensor. Secondly, it involves the stability exhibited by the functional layer of the elastic body throughout the actual working process. It is worth noting that this repeatability parameter is, to a significant extent, influenced by a multitude of environmental factors, such as temperature variations, diverse humidity conditions, and external electromagnetic interference, among others.

**Fig. 19:**
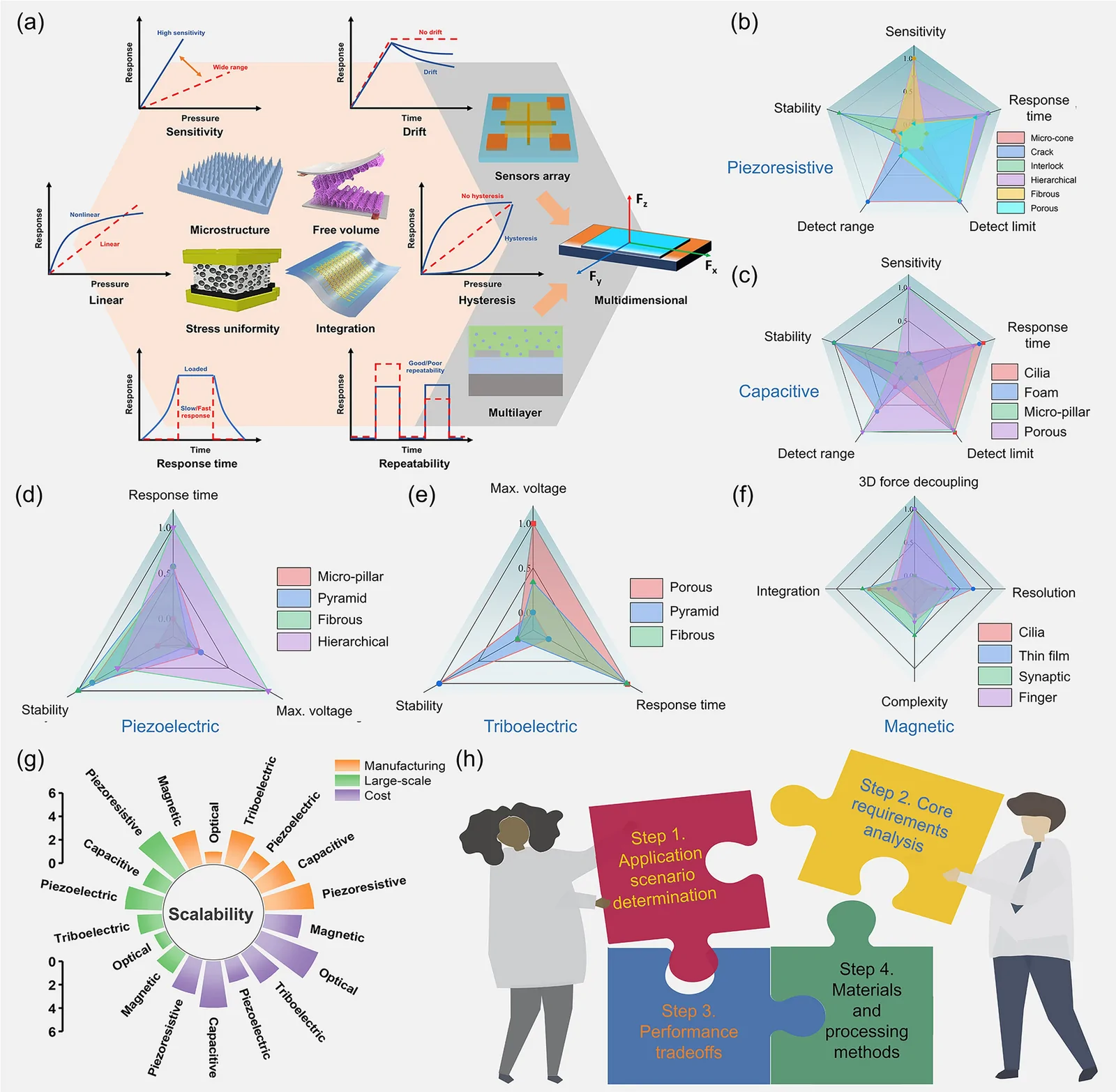
**a** The performance assessment indicators for tactile sensors encompass sensitivity, linearity, hysteresis, drift, response time, repeatability, and other aspects. **b**–**f** The key performance comparison of tactile sensors with different mechanism and structural designs. **g** Comparative analysis of scalability in manufacturing, mass production, and cost across different tactile sensing mechanisms. **h** Reference workflow for rational design of tactile sensors

The heterogeneous nature of performance metrics across tactile sensing modalities stemming from non-uniform parameter units and dimensional inconsistencies presents formidable challenges in establishing unified criteria for holistic sensor evaluation. To address this, we conducted a comprehensive review of emerging tactile sensor technologies, systematically categorizing their operational principles, key performance indicators (KPIs), and structural configurations, as tabulated in Table 3. For cross-modal benchmarking, critical parameters sharing identical dimensional definitions were normalized using min–max scaling. This enabled the quantitative visualization of comparative performance through a radar chart array (Fig. 19b–f), where each axis represents a normalized KPI and vertices denote sensor-specific values. Figure 19g illustrates the scalability of different tactile sensor types under emerging evaluation criteria, including manufacturing difficulty, scalability potential, and cost.

**Table 3:** Performance comparison of tactile sensors with different working mechanisms and structures

Mechanisms	Materials	Structures	Sensitivity	Detect limit	Detect range	Response time	Stability (cycles)	References
Piezoelectric	Ag NWs/PDMS	Micro-cone	15.66 kPa^−1^	0.2 Pa	N/A	42.4 ms	4000	[58]
	Mxene/PDMS	Micro-cone	62 kPa^−1^	0.1 Pa	0–3.0 KHZ	15 ms	N/A	[59]
	PET/PDMS	Crack	GF = 113.7	N/A	0–120% strain	N/A	1000	[66]
	Mxene@ZnO/PU	Interlock	236.5 kPa^−1^	82 Pa	0–260 kPa	100 ms	10,000	[69]
	GR/CNT/PDMS	Interlock	0.0338 kPa^−1^	N/A	0.062–450 kPa	100 ms	3000	[70]
	MXene	Hierarchical	528.87 kPa^−1^	0.6 Pa	0–30 Pa	45 ms	8000	[72]
	WPU/SWCNT/CNF	Lattice	7.31 kPa^−1^	N/A	2.8–25.7 kPa	N/A	10,000	[73]
	S-MXene	Porous	9.859 kPa^−1^	12 Pa	0–200 kPa	224 ms	1000	[74]
Capacitive	PDMS	Micro-cone	44.5 kPa^−1^	0.14 Pa	0–35 kPa	N/A	5000	[75]
	PDMS/CB	Foam	35 kPa^−1^	N/A	0–16 kPa	N/A	100	[79]
	Graphene/PDMS	Microcilia	0.0079 kPa^−1^	30 Pa	30 Pa-300 kPa	60 ms	5000	[83]
	PDMS	Porous	0.21 kPa^−1^	5 Pa	0–5 Pa	112 ms	3000	[84]
	PVA/H_3_PO_4_	Gradient dome	0.214 kPa^−1^	6.67 Pa	10–100 kPa	90 ms	8000	[85]
	PDMS/MWNTs	Gradient Slant	0.12 kPa^−1^	3 Pa	0–130 kPa	46 ms	3000	[86]
Piezoelectric	PDMS/PVDF	Micro-pillar	346.5 pC/N	N/A	0.009–4.3	N/A	N/A	[95]
	PVDF/DA	Fibrous	7.92 V/N	15 V	1.5–40 N	N/A	10,000	[99]
	PZT	Hierarchical	0.062 kPa^−1^	N/A	0–40 kPa	23 ms	N/A	[100]
	BCZT	Droplet-shaped	310 pC/N	43 V	0–16 N	N/A	5000	[102]
Triboelectric	Cellulose	Spherical surface	9.21 kPa^−1^	120 V	4.17–100 kPa	70 ms	2000	[109]
	Ecoflex	Hierarchical	N/A	43 V	N/A	N/A	1000	[110]
	PDMS/Ag NWs	Pyramid	N/A	0.35 kPa	N/A	120 ms	1200	[112]
	PU/PTFE/Al	Sensor array	N/A	13 V	N/A	1.23 ms	5000	[24]
	Iontronic gel	Phase-locked	0.383 kPa^−1^	37 V	4.2–100 kPa	69 ms	2000	[113]
Magnetic	PDMS/NbFeB	Microcilia	1.43 µT/(m/s)	2.1 μN	0–60 μN	N/A	10,000	[114]
	Ni/Fe/IrMn	Synaptic	0.126 mV/KPa	6 Pa	6 Pa-400 kPa	40 ms	2000	[118]
	Ecoflex/NbFeB	Finger-like	0.83 μV/N	0.04 N	8–98.68 kPa	N/A	10,000	[120]
	NbFeB	Thin film	N/A	2.1 mm	3600 mm^2^	N/A	5206	[121]
	NbFeB	Thin film	N/A	1.2 mm	48,400 mm^2^	N/A	N/A	[122]
Optical	PDMS/Optical fiber	Droplet-shaped	− 6.398 MHz/N	0.8 mN/0.2℃	0–40 mN	N/A	1000	[126]
	PDMS/Optical micro/nanofibers	Bulb-shaped	0.108 mN^−1^	0.031 mN	0–4.4 N	N/A	3000	[127]
	PDMS/Optical fiber	Knot	125.21 MPa^–1^	N/A	0–1250 kPa	50 ms	4000	[129]

A systematic tactile sensor design encompasses a multidimensional framework integrating sensing mechanism, material selection, performance characteristics, and scalability considerations. As outlined in Fig. 19h, this systematic design process involves the following sequential steps: (1) definition of target application scenarios; (2) identification of core performance requirements; (3) determination of the sensing mechanism through performance trade-off analysis; and (4) selection of materials and fabrication methods. Such a design principle will advance application-oriented tactile sensor design.

## Systems Integration and Intelligent Algorithms

The new generation of tactile sensing systems is advancing in the directions of miniaturization, integration, multifunction, and intelligence. Apart from enhancing the performance of individual sensors, the integration of the entire system also constitutes a significant research area worthy of in-depth exploration. A comprehensive tactile sensor system is expected to incorporate multifunctional sensors that bear similarities to those found in the human body, an independent and reliable power supply system, as well as highly efficient wireless communication capabilities [252–254]. When it comes to the design of multifunctional sensors, researchers typically resort to two fundamental design strategies. The first one is the in situ multifunctional integration of a single device. This approach involves endowing a single sensor device with multiple functions during its manufacturing process, allowing it to perform various sensing tasks simultaneously without the need for additional separate devices. The second strategy is the physical combination of multiple sensor devices with distinct functions. By carefully assembling different types of sensor devices together, a more comprehensive sensing functionality can be achieved, covering a wider range of sensing requirements [255, 256].

With regard to the power supply aspect, as the theories of piezoelectricity and triboelectricity have been continuously refined and improved over time, self-powered devices based on these principles have witnessed extensive research and development efforts. Moreover, another highly effective means to attain energy autonomy for tactile sensors is through the integration of intrinsically transparent tactile sensing units with perovskite solar cells. The transparency of the tactile sensing units ensures that they do not interfere with the light absorption and conversion processes of the perovskite solar cells, while the solar cells, in turn, can provide a stable and sustainable power source for the tactile sensors, enabling them to operate independently without relying on external power supplies.

In order to facilitate signal processing and seamless data transmission, the integration of sensors with wireless communication components has been put forward as a viable solution. This integration allows the tactile sensors to not only detect and collect relevant data but also transmit this data wirelessly to other devices or systems for further analysis and utilization. By combining the sensing capabilities of the sensors with the communication capabilities of the wireless components, a more intelligent and interconnected tactile sensor system can be established, which holds great potential for various applications in fields such as robotics, healthcare, and wearable technology.

**Fig. 20:**
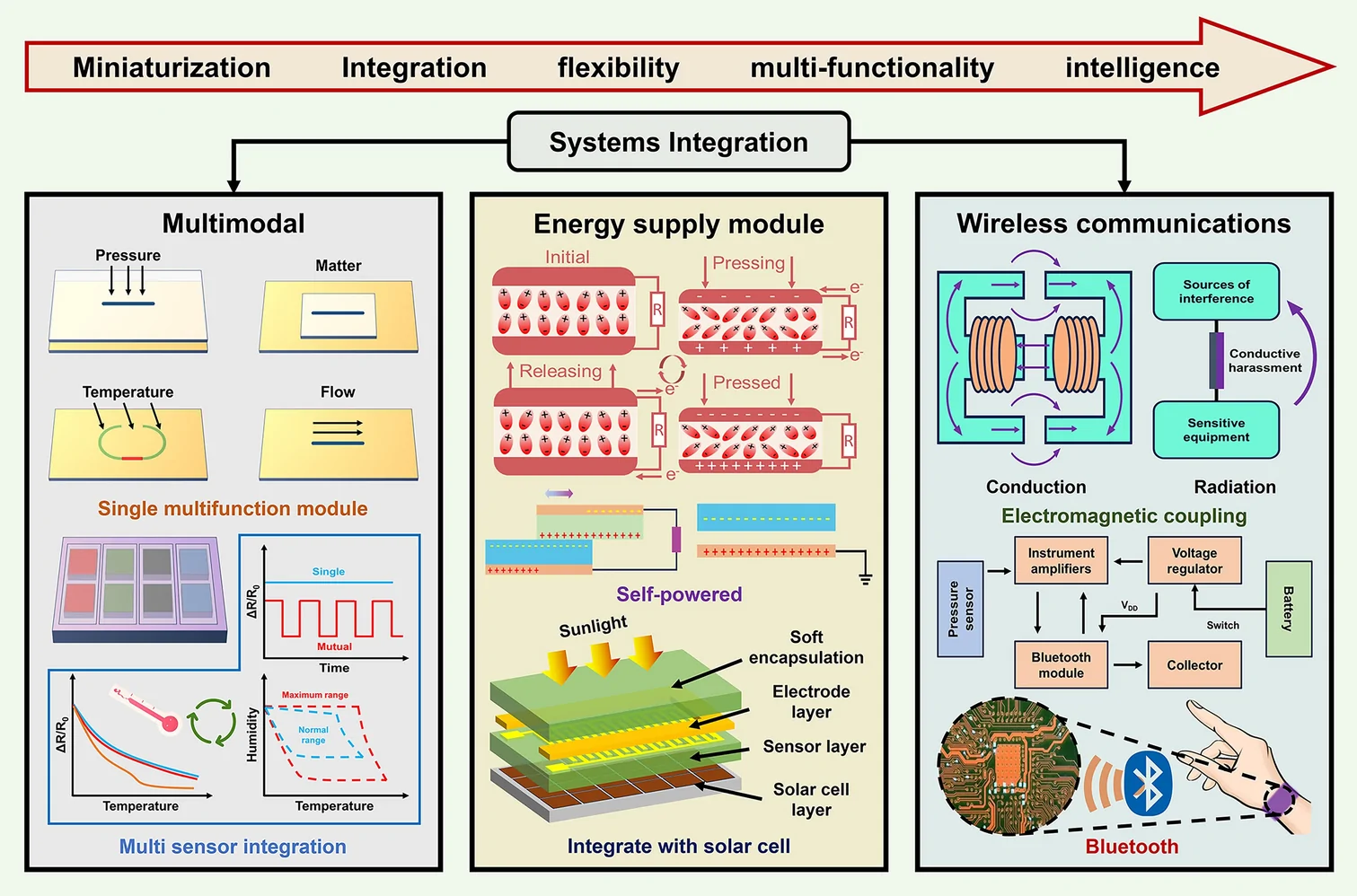
System integration of tactile sensors includes multifunctional sensing modules, energy-autonomous modules, and wireless communication modules

In addition, efficient and reliable system integration necessitates addressing three core challenges: electronic interface compatibility, signal processing complexity, and practical deployment. The selection of integration methods requires co-design across materials, structures, circuits, and systems. At the material-structure level, direct integration of sensitive materials with substrates can be achieved through coating, injection molding, or 3D printing. Alternatively, photolithography or thin-film deposition enables seamless material-substrate bonding. For sensor-circuit integration, rigid-flex interconnects (e.g., soldering) or multilayer stacking are commonly employed. At the system level, traditional modular components are interconnected via standardized interfaces. Wireless transmission offers superior flexibility (eliminating physical cable constraints), enhanced convenience (simplifying system architecture), and broader scenario adaptability (avoiding infection risks associated with wired connections in brain–computer interfaces) compared to traditional interfaces. This co-design philosophy collectively facilitates diverse practical application scenarios. The integration process of the complete tactile sensing system is shown in Fig. 20.

### Novel Integration Technologies

**Fig. 21:**
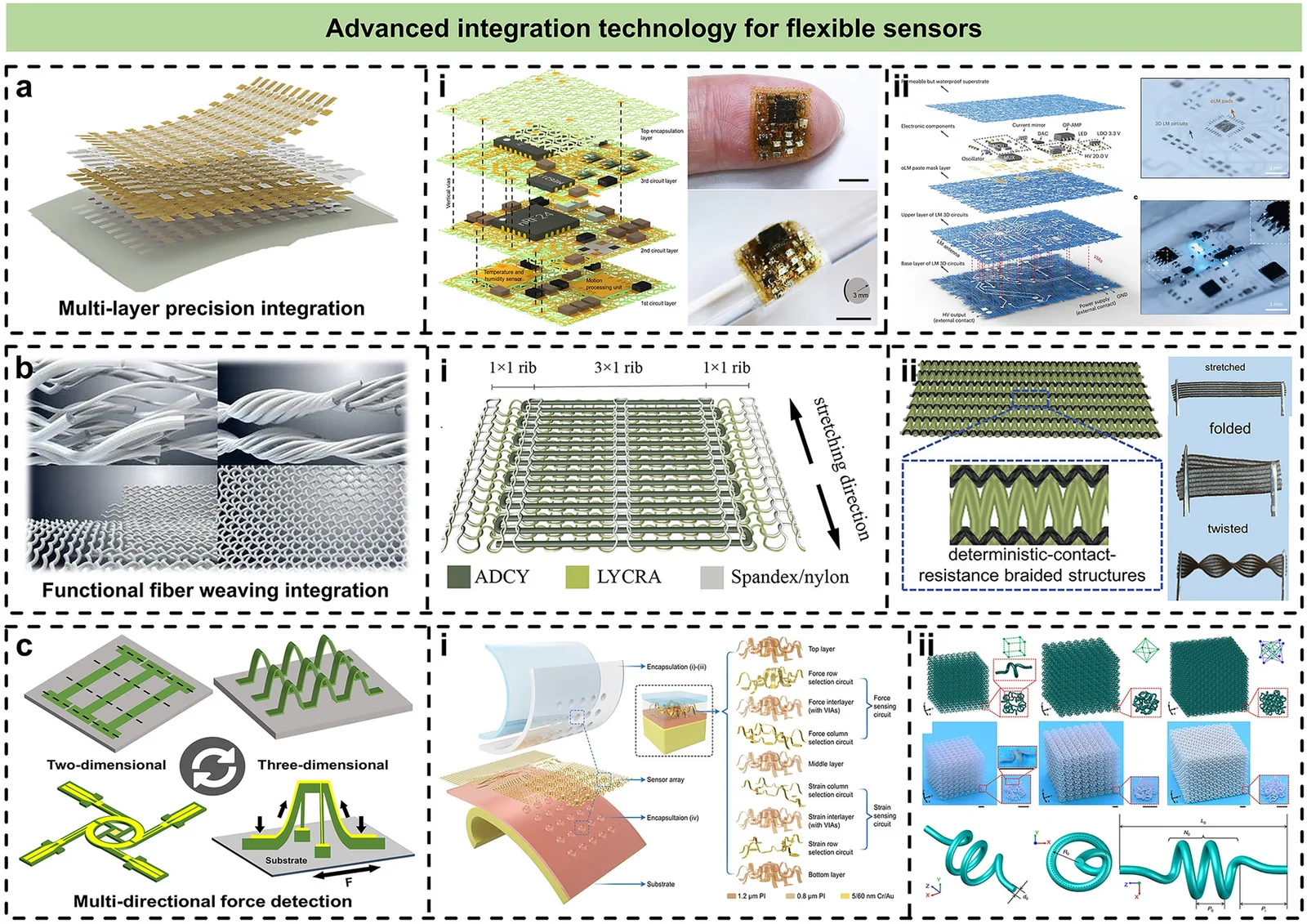
**a** Integrated sensing of multilayer structures: (i) integration/packaging strategy based on SMNM [257], (ii) permeable 3D integrated electronic skin [258]. Copyright 2022, The American Association for the Advancement of Science. Copyright 2024, Springer Nature. **b** Integrated sensor components with textile structures: (i) Textile resistive strain sensor (TRSS) [259], (ii) Manufacturing process of the strain sensor based on DCRBS [260]. Copyright 2024, Donghua University. Copyright 2022, Wiley–VCH. **c** Advanced integration technologies of three-dimensional structurals: (i) integration technology of three-dimensional bionic electronic skin [261], (ii) 3D network materials with different 3D topological structures [262]. Copyright 2024, The American Association for the Advancement of Science. Copyright 2020, The Author(s)

With the continuous progress of sensing technology, the demands placed on tactile sensors regarding smaller dimensions, lower power consumption, and enhanced reliability are escalating on a daily basis. Zhang et al. put forward a laminated grid packaging technology for miniaturized and highly integrated flexible electronic devices [257]. This technology enabled the realization of inorganic flexible electronic devices that possess high elongation rates, high coverage ratios, and mechanical properties similar to those of human skin, thereby resolving the conflict between the high elongation rate and high coverage rate of the packaged flexible electronic devices. In this research, stacked multilayer network materials were introduced as a universal platform for integrating individual components and stretchable interconnections, without imposing any fundamental limitations on their deformation. The schematic illustration of the miniaturized electronic system (11 mm × 10 mm) fabricated based on this strategy is presented in Fig. 21a(i). To achieve high-density and multifunctional stretchable electronic products, it is necessary to develop 3D-stretchable integrated systems. Zheng et al. reported a permeable 3D integrated electronic skin (P3D-eskin), which combines high-density inorganic electronic components with an organic stretchable fiber substrate by using 3D patterning, multilayer LM circuits, and hybrid liquid metal (hLM) solders [258]. The 3D integration between different layers was achieved by designing the vertical penetration of LM to form stretchable vertical interconnect access (VIA). The hierarchical integration process of the device is shown in Fig. 21a(ii).

Besides the hierarchical integration approach, the advanced integration strategy relying on knitting techniques has caught researchers' attention too. Yet, it is still tough to make sensors that can be seamlessly integrated into clothing. Liu et al. put forward a textile resistive strain sensor (TRSS). Via an industrial knitting process, conductive yarns covered with waterproof and antioxidant acrylic/copper composite fibers were selectively inserted into a highly elastic substrate (Fig. 21b(i)) [259]. These conductive yarns were folded and closely piled up. The contact resistance changes due to the separation of adjacent yarn segments during stretching, enabling strain sensing. Similarly, Su et al. proposed a stretchable strain sensor based on the deterministic contact resistance braiding structure (DCRBS), as shown in Fig. 21b(ii) [260]. In the DCRBS, polyester yarns tightly bound silver fibers and latex threads to form a periodic "Y"-shaped structure, which prevented fiber slipping during stretching, leading to small relaxation and high repeatability of the strain signal, and thereby showed excellent performance and was easy to integrate into electronic clothing.

The 3D-structured electronic skin is a novel sensing technology, equipped with multidimensional perception capabilities and enhanced sensitivity. Meanwhile, due to its structural characteristics on the three-dimensional scale, it has better integrability. Inspired by the hierarchical perception characteristics of human skin, Zhang et al. reported an electronic skin with a 3D architecture (denoted as 3DAE-Skin), in which the force and strain sensing components are arranged in a 3D layout, mimicking the Merkel cells and Ruffini endings in human skin. The sensor device exhibits excellent decoupled sensing performance in terms of normal force, shear force, and strain, and the device structure is shown in Fig. 21c(i) [261]. Inspired by the 3D network structures of curved filamentous structures in biological tissues, Zhang et al. reported a class of soft 3D network materials. This material system utilizes lattice configurations with different 3D topological structures. The developed soft 3D network materials provide J-shaped stress–strain responses under compression and stretching, almost along any loading direction. The 3D network structure is shown in Fig. 21c(ii) [262].

### Multisensory Functions Integration

**Fig. 22:**
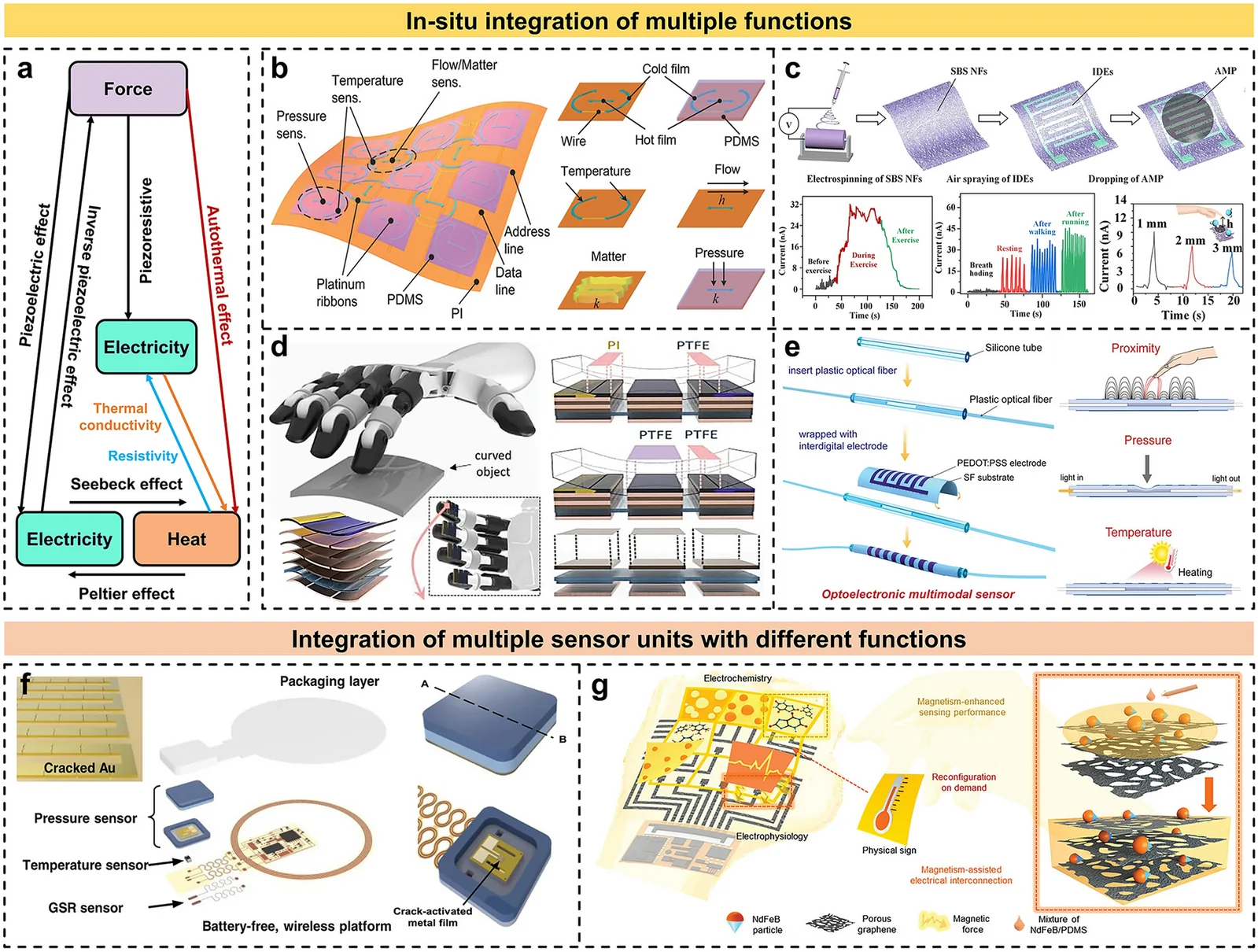
**a** Common sensing mechanisms of tactile sensors and the piezothermal mechanism, which is analogous to the piezoresistive sensing mechanism; **b** flexible e-skin with staggered arrangement of multifunctional sensing units [263]. Copyright 2017, WILEY–VCH. **c** Fabrication process of multimodal sensors based on SAMP [264]. Copyright 2024, The Author(s). **d** Intelligent robot finger equipped with TMTS sensors [265]. Copyright 2024, Wiley‐VCH. **e** Schematic diagram of the structure of a flexible photoelectric multimodal sensor [266]. Copyright 2023, Wiley‐VCH. **f** Schematic diagram of a battery-free wireless sensing platform including a crack-activated pressure sensor, a temperature sensor, and a GSR (galvanic skin response) sensor [267]. Copyright 2021, The Author(s). **g** Schematic diagram of reconfigurable soft electronic devices in HMGN [268]. Copyright 2024, Wiley‐VCH

Increasingly in-depth research on multimodal devices has led researchers to focus more on developing multifunctional responsive materials and devices with new sensing mechanisms. The diverse working mechanisms of tactile sensors fundamentally represent the conversion processes between mechanical stimuli and electrical signals. Nevertheless, human tactile sensations encompass not only responses to external forces but also the intuitive perception capabilities regarding parameters like temperature and humidity. In a manner analogous to the alteration in resistivity of the functional layer of sensor devices brought about by external forces in the piezoresistive effect, Zhu and his colleagues put forward a sensing mechanism predicated on the change in thermal conductivity of materials induced by external forces [263]. Concurrently, a multifunctional electronic skin founded on thermal sensation was also proposed, which exhibits functions that closely approximate those of human tactile senses. These functions include the perception of temperature and pressure stimuli, as well as the discrimination of substance types and the sensing of wind forces, as depicted in Fig. 22b. The thermally sensitive platinum (Pt) thin film deposited on a flexible polyimide substrate was utilized to mimic multiple sensors on human skin. The Pt thin film was patterned into multiple strips that formed an array, thereby configuring multisensing elements. The Pt elements were electrically heated to the temperature of human skin. When the heated Pt element comes into contact with a certain substance, due to conductive heat transfer, the temperature of the hot element will respond to the thermal conductivity of the substance, which furnishes a means of distinguishing substances. This device allows for the in situ integration of multiple functions within a single sensor device, and its uncomplicated structure renders large-scale preparation feasible.

As an essential part of tactile perception, humidity sensing plays a crucial role in enabling the conveyance of more intricate tactile information. Zhang and his colleagues developed a skin-conformal and breathable humidity sensor. This sensor was fabricated by utilizing MXenes composite materials along with electrospun elastomeric nanofibers that feature patterned electrodes, thereby accomplishing the recognition of motion states as well as emotional patterns. The fabrication process of this device is illustrated in Fig. 22c [264]. Owing to the high efficacy and low cost of the triboelectric mechanism, it has been extensively researched for the purposes of measuring pressure and identifying materials to augment robotic perception. Lee and his team proposed a triboelectric multimodal tactile sensor (TMTS) with a multilayer structure design [265]. This sensor is capable of simultaneously identifying different materials, curvatures, and pressures, thus decoupling different modalities to attain more precise detection. Two functional elements were integrated. A paper and a surface textured Ecoflex with three columns are dedicated to pressure sensing at its base (Fig. 22d). Also based on the design concept of in-situ integration of multimodal sensing, Wang et al. reported a flexible photoelectric multimodal sensor. This sensor is capable of detecting and decoupling proximity/pressure/temperature by integrating an optical waveguide and interdigital electrodes (IDE) into a compact fiber sensor [266]. As shown in Fig. 22e, on a regenerated silk fibroin (SF) substrate, the optical waveguide composed of PEDOT:PSS patterns and the flexible interdigital electrodes (IDE) are encapsulated into a compact fiber sensor. The fabricated device has achieved self-decoupling multimodal perception for proximity/pressure/temperature stimulus sensing.

Apart from the in situ integration strategy aimed at multiple functions, integrating numerous sensors with distinct sensing capabilities into a single sensing platform represents another viable implementation approach. In the study conducted by Park et al., a battery-free, wireless, multimodal sensor along with a mobile system, which was designed for the continuous measurement of pressure, temperature, and electrodermal activity at the skin interface, was introduced, as illustrated in Fig. 22f [267]. This sensing platform was crafted by integrating pressure, temperature, and electrodermal sensors, thereby attaining a multimodal sensing system endowed with multiple functions. Likewise, based on the design notion of integrating multiple devices, Han et al. made use of laser-induced porous graphene as the sensing material and doped it with permanent magnetic particles to fabricate hard magnetic graphene nanocomposites (HMGN) [268]. These composites were capable of self-assembling onto a flexible supporting substrate in a reversible and reconfigurable fashion through magnetic force. Owing to the fact that HMGN possesses the dual functions of serving as both a sensing material and a connecting component within soft electronics, it may demonstrate continuous, multimodal, and customizable measurement of various types of signals. The structure of the device is depicted in Fig. 22g.

### Energy Supply

The energy supply module holds a vital position within the tactile sensing system. When integrated into the system, it is capable of furnishing a continuous and stable power supply to the sensor components, thereby guaranteeing the uninterrupted flow of sensing signals. Moreover, in light of the diverse working mode requirements across various scenarios, a power supply equipped with the functionality of flexible output adjustment ensures that the power consumption aligns precisely with the specific application scenarios. The stability of the power supply not only has an impact on the accuracy of the sensor's signals but also on its response speed. Additionally, it provides a foundation for the further integration with the communication module. Generally speaking, there are two common approaches to realizing the energy supply of the device: One is through integration with the solar cell system, and the other is by constructing self-powered sensor components using triboelectric and piezoelectric materials.

**Fig. 23:**
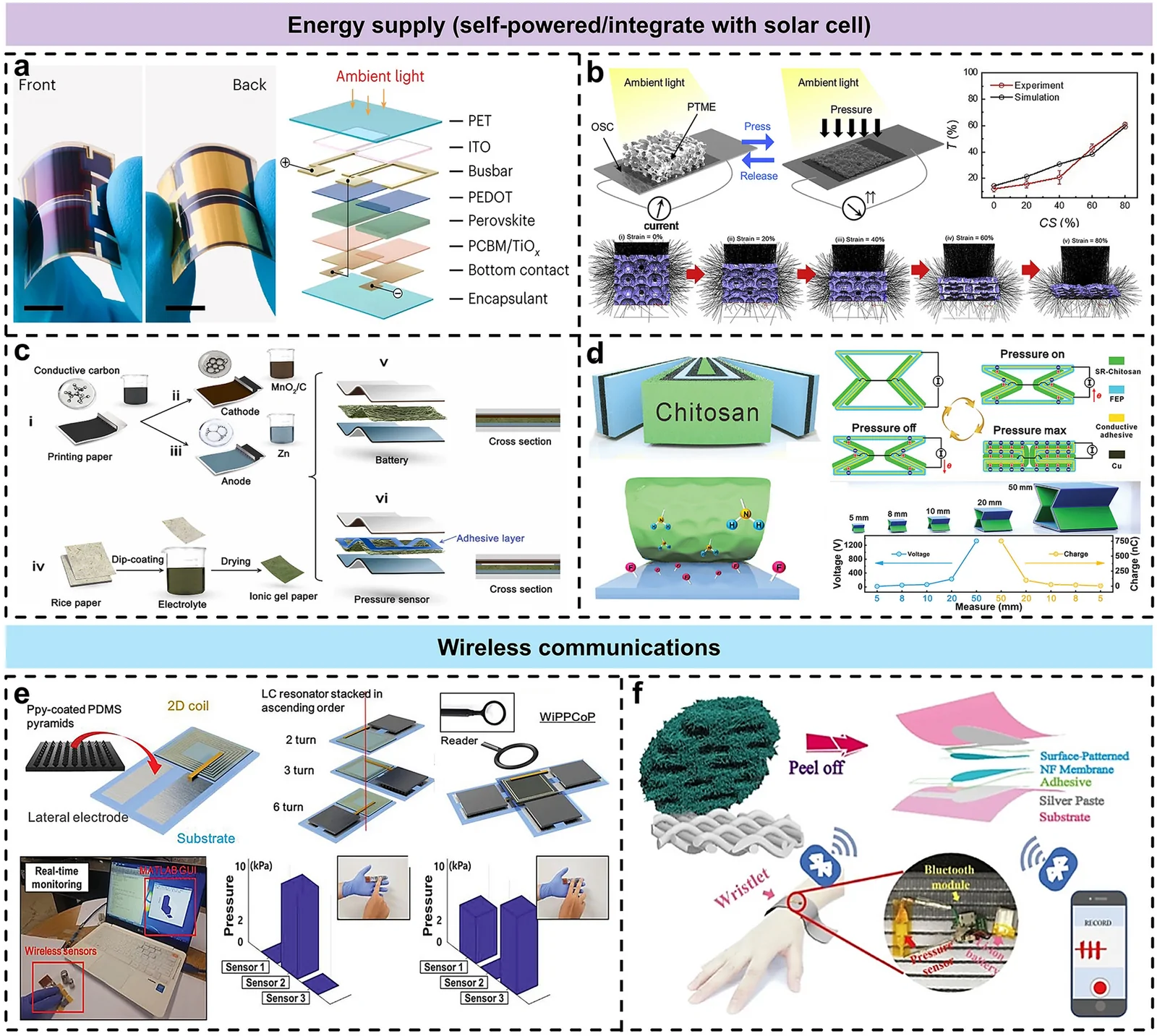
**a** Three-dimensional model of the FPSC module architecture [269]. Copyright 2023, Springer Nature. **b** Integration of the microporous elastomer (PTME) with the thin-film organic solar cell [270]. Copyright 2020, Elsevier. **c** Paper-based sandwich solid Zn-MnO2 battery tactile sensor [271]. Copyright 2024, Elsevier. **d** Triboelectric tactile sensor made of origami-structured biomass materials [272]. Copyright 2022, Wiley–VCH. **e** Wireless pressure sensing platform based on electromagnetic coupling [275]. Copyright 2019, WILEY–VCH. **f** Bluetooth wireless sensing module integrated with tactile sensors [276]. Copyright 2018, American Chemical Society

The commercially dominant photovoltaic technology based on silicon is suitable for large-scale solar energy collection. However, due to the drawbacks of being fragile and bulky, silicon cells are difficult to be practically applied in flexible sensors. Perovskite solar energy has witnessed rapid development thanks to its inherent properties, such as long carrier diffusion length, high absorption coefficient, solution processability, small exciton binding energy, high tolerance to structural defects, adjustable bandgap, and high photoluminescence quantum yield. Min et al. reported an autonomous wearable biosensor powered by a flexible perovskite solar cell (FPSC), which can provide continuous and non-invasive metabolic monitoring (Fig. 23a) [269]. The device adopts a p-i-n structure and is fabricated by laminating flexible perovskite layers with light-transmitting functional materials, realizing the effective integration of the energy supply module. Similarly, in line with the design concept of integrating sensor components and solar cell modules, Choi et al. put forward a new type of self-powered pressure sensor (the self-powered pressure sensor based on piezoelectric transmittance, abbreviated as PTSPS). This sensor was fabricated through the integration of a microporous elastomer (PTME), piezoelectric transmittance (which alters the optical transmittance upon the application of pressure), and a thin-film organic solar cell (OSC) [270]. The operational mechanism of this device lies in transforming the alterations of external mechanical stimuli into changes in the light transmittance of the PTME layer. Subsequently, the sensing of external stimuli was achieved via the response of the thin-film organic solar cell. The distinct working states of the PTME layer under varying compressive strains of the device are depicted in Fig. 23b.

In addition, tactile sensors relying on the triboelectric and piezoelectric principles have emerged as promising contenders for energy-autonomous sensor components, thanks to their uncomplicated structures and high level of integration-friendliness. Nevertheless, given that the output of such devices hinges on the rate of change of external stimuli, they are not applicable to the measurement of static mechanical properties. To surmount this constraint, Li et al. devised a self-powered sensor capable of measuring both static and dynamic pressures by integrating it with a chemical battery. Through a meticulous structural design, a double-sided tape (3 M) frame was employed to separate the cathode and electrolyte of the solid-state Zn–MnO_2_ battery, as shown in Fig. 23c [271]. This ensured that they remained out of contact during the initial stage, thereby enabling the formation of self-powered pressure sensors in various sizes. As the external load augments, the contact area between the electrode and the electrolyte expands continuously. This, in turn, brings about a reduction in the interfacial resistance of the sensor and an elevation in the output current. In order to further gather the information acquired from tactile perception, Wang et al. introduced a brand-new visual tactile sensor, as shown in Fig. 23d [272]. This sensor accomplished self-powered tactile feedback without the necessity of an external power supply, thereby freeing the sensor from the shackles of energy concerns. By utilizing biomass materials possessing outstanding triboelectric properties and implementing a combined technique of origami and surface engineering, the friction area of the triboelectric nanogenerator (TENG) was enlarged. Simultaneously, the visualization device was mounted on the wristband, enabling the completion of real-time tactile feedback through the alterations of the LED light strip.

### Wireless Communication

The transmission and processing of sensing signals constitute the terminal modules within a complete tactile sensing system. For highly integrated sensing platforms, wireless transmission is the favored approach. Generally speaking, wireless sensing technologies can be broadly categorized into two main types: electromagnetic coupling and Bluetooth communication. Electromagnetic coupling takes place between the internal LC or RLC resonator and the external resonator that is connected to the readout system. When a stimulus is applied, it will cause a change in the resonant frequency within the effective inductance or capacitance of the internal resonator [273, 274]. Here, the offset resonant frequency (f) is defined by the following formula:

6.4.1 $$f=\frac{1}{2\pi \sqrt{{L}_{\mathrm{e}}{C}_{\mathrm{e}}}}$$

Here *L*_e_ and *C*_e_, respectively, represent the effective inductance and capacitance of the internal resonator.

The coupling of multiple tactile information sources can substantially augment the complexity of the signals, which is an issue that lies beyond the capabilities of conventional devices to address. Lee et al. introduced the WiPPCoP (Wireless Parallel Pressure Cognition Platform, as shown in Fig. 23e), which employed a vertically stacked two-dimensional coil architecture to wirelessly receive pressure information from multiple sensors concurrently (i.e., in parallel) at a single location [275]. Subsequently, it amalgamated these signals into a representative signal pattern. Each coil within this configuration was characterized by a specific number of turns. The sensing coil assigned a distinct resonant frequency within the reflection coefficient spectrum to each sensing element, thereby facilitating the discrimination of signals. On the other hand, Bluetooth represents another prevalent wireless sensing module. The integration of Bluetooth sensing systems has been reported on multiple occasions. For instance, Zhong et al. presented a wireless wearable pressure sensor, which accomplished the efficient transmission of tactile sensor signals to mobile phones through Bluetooth communication, thereby demonstrating its application potential within the realms of real-time human–machine interfaces and intelligent robots [276]. The device structure and the Bluetooth module are shown in Fig. 23f.

### Machine Learning Algorithms

Tactile sensors are continuously evolving in the directions of miniaturization, multifunctional sensing, and expandability. Given that multifunctional sensors are typically associated with the coupling of multiple signals, they not only bring about more precise sensing information but also present challenges regarding operating speed, signal decoupling, and information extraction. Machine learning tools, which possess the merits of high accuracy, strong robustness, and rapid computation, have emerged as a widely favored signal processing technology [277, 278].

Sensor input signals generally fall into two main categories: analog signals and spike signals. Typical tactile sensors like piezoresistive, piezoelectric, and optical ones usually output continuous signals with arbitrary values, which are categorized as analog signals [279–281]. In contrast, spike signals obtained within the realm of neurology are, in essence, the occurrences of characteristic events with temporal precision [282, 283]. Regarding different types of signals, researchers have devised a diverse range of machine learning algorithms for the purpose of processing and making use of these signals. The signal processing process in machine learning can be segmented into several components: data acquisition, preprocessing, feature extraction, and the application of machine learning algorithms [284]. Once tactile sensors have gathered signals, the data possessing different characteristics need to be converted into a unified standard. The process of achieving feature extraction through preprocessing is a common procedure across all data processing operations. High-dimensional tactile signals present challenges to feature extraction, and these challenges are typically tackled during the intermediate stage of machine learning. On the one hand, the selection of an effective feature extraction strategy can considerably diminish the complexity of machine learning algorithms. On the other hand, proficient machine learning algorithms typically accomplish feature extraction satisfactorily during the intermediate stage. The appropriate combination of these two aspects offers a potent impetus to researchers' data processing endeavors.

**Fig. 24:**
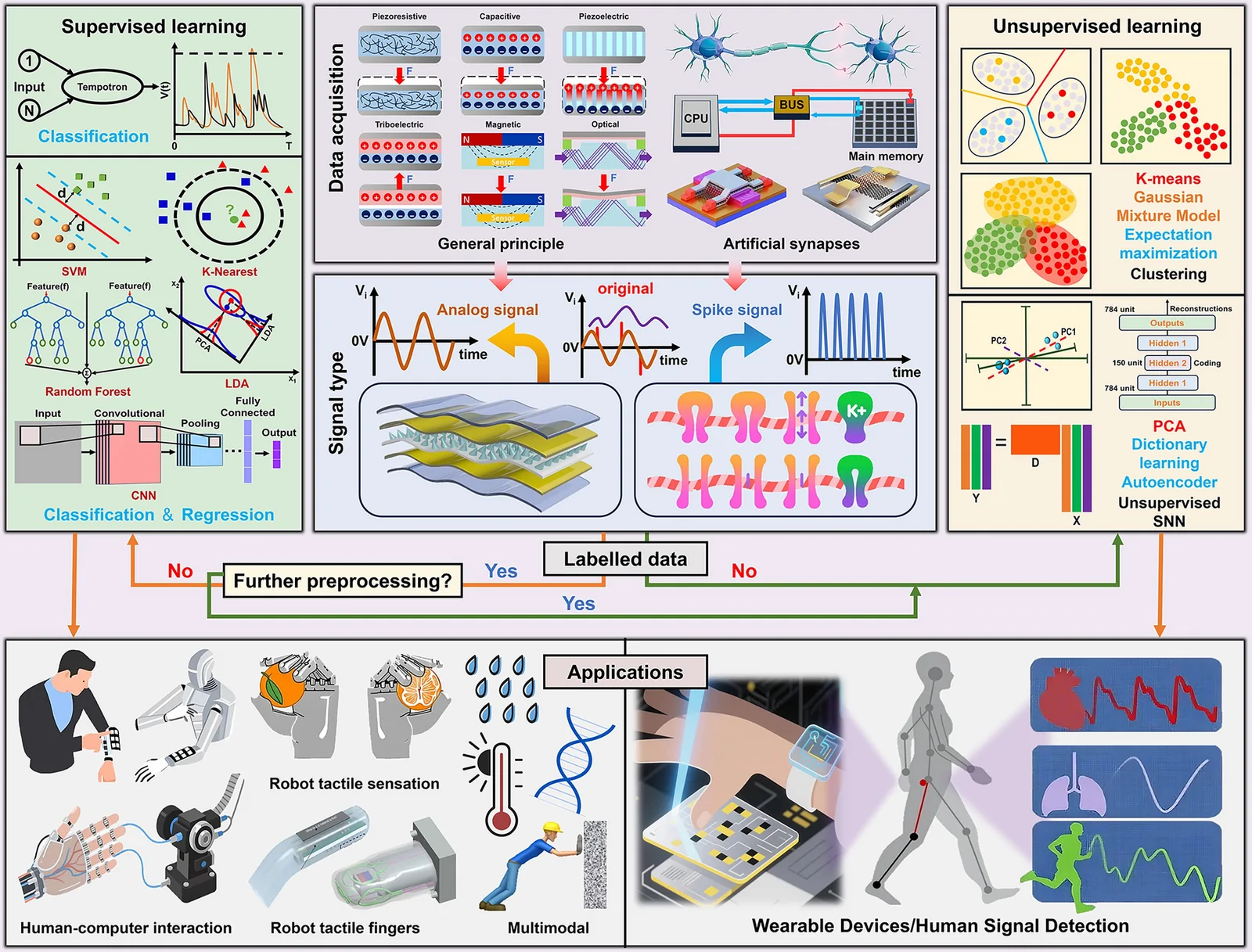
Summary of machine learning tasks, algorithms, and application scenarios. Copyright 2022, The American Association for the Advancement of Science. Copyright 2024, The Author(s)

Machine learning tasks can be categorized into four main types: classification, regression, clustering, and dimensionality reduction [37, 285, 286]. In the context of classification and regression tasks, the data are labeled, and the algorithms function in a predictive capacity, thereby falling within the realm of supervised learning [287, 288]. Classification tasks are concerned with discrete labels, whereas regression tasks are focused on handling continuous labels. Illustrative examples of such tasks include those related to the surface characteristics of objects and the spatial distribution of object surface loads. In contrast, for clustering and dimensionality reduction tasks, the data remain unlabeled, and the underlying patterns need to be unearthed through exploration. These tasks are thus classified under the umbrella of unsupervised learning. In addition to supervised learning and unsupervised learning, reinforcement learning is another typical type of machine learning algorithm. Reinforcement learning is often applied in the field of intelligent robot manipulation to make decisions regarding the actions of robots. The commonly used machine learning algorithms and their application scenarios are shown in Fig. 24.

#### Unsupervised Learning

**Fig. 25:**
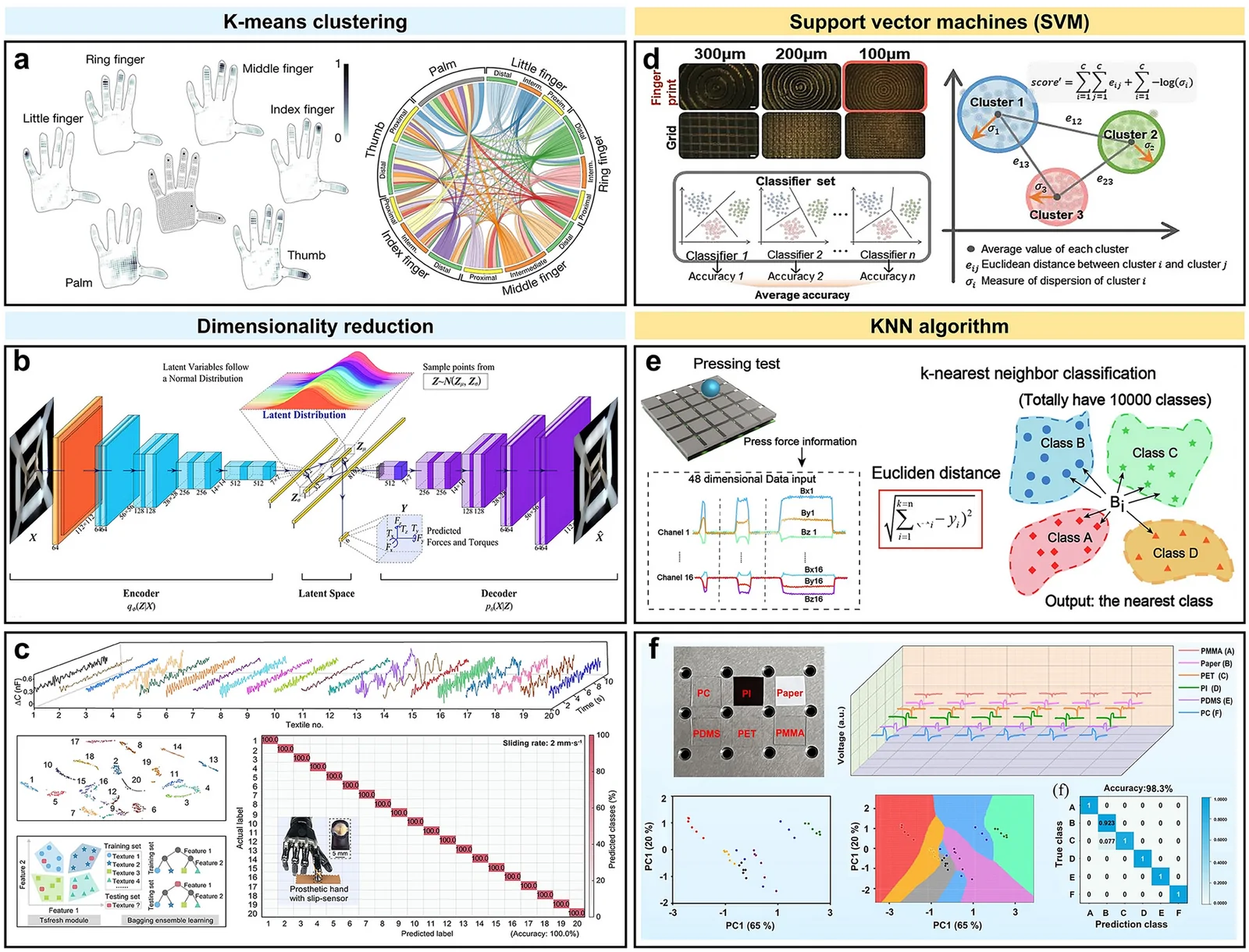
**a** Perform data clustering using the k-means clustering algorithm [289]. Copyright 2019, Springer Nature. **b** Perform dimensionality reduction using autoencoders [290]. Copyright 2023, WILEY–VCH. **c** Conduct dimensionality reduction with the t-SNE algorithm [291]. Copyright 2023, The Author(s). **d** Use support vector machines (SVM) to detect force, touch location, and hand gestures [38]. Copyright 2023, WILEY–VCH. **e** Recognition using the K-nearest neighbors (KNN) algorithm [122]. Copyright 2021, The American Association for the Advancement of Science. **f** The contact force estimation method based on long short-term memory (LSTM) [292]. Copyright 2025, Elsevier

K-Means is a prevalently utilized clustering algorithm within the realms of data mining and machine learning. Its principal objective is to partition a given dataset into K clusters, ensuring that the similarity among data points within the same cluster is high, while that between different clusters remains low. The fundamental process entails determining the value of K, initializing the cluster centers, allocating data points to respective clusters, updating the cluster centers, and iterating this sequence. In the domain of tactile sensing, K-Means clustering has been employed to considerably diminish the redundancy of continuously gathered data (refer to Fig. 25a) [289]. Sundaram devised an expandable tactile glove for data collection, which is composed of a piezoresistive film and 548 conductive threaded electrodes, with the aim of exploring the characteristics of human grasping. Data is collected when participants grasp various objects, and subsequently, N frames are fed into a deep neural network for classification. To curtail the data redundancy during the evaluation, the strategy of maximizing data variance is adopted. Firstly, the dimension of the tactile signal is reduced, and then K-Means clustering is utilized to identify N clusters. For any randomly selected input, this frame will be complementary to the N-1 frames from other clusters, and subsequently, they will all be input into the deep neural network jointly.

In high-dimensional datasets, due to the excessive number of dimensions, it is almost impossible to perform direct visualization. Humans can hardly intuitively understand and grasp the data distribution and relationships in high-dimensional space. Dimensionality reduction algorithms can map high-dimensional data to low-dimensional spaces (such as two-dimensional or three-dimensional), enabling the data to be presented in a graphical manner. High-dimensional data implies higher consumption of computational resources. By reducing the dimensions of the data, the resource consumption during the computational process is significantly decreased. To find the low-dimensional representations of tactile data, principal component analysis (PCA), dictionary learning, and autoencoders are three commonly used methods by researchers. Directions with larger variances contain more information. Therefore, PCA is proposed to find the principal components that are the directions where the data projections change the most. PCA can identify meaningful directions from unlabeled data, thus processing high-dimensional data to a manageable size for further data analysis. Sparse dictionary learning aims to find the sparse representation of data on a dictionary in the form of linear combinations of dictionary atoms. For a dataset, dictionary learning will find a fixed-size dictionary in which the data representation has the maximum sparsity using the atoms in the dictionary.

An autoencoder is a type of feedforward neural network that encodes itself in the intermediate layer and then predicts the original input in the output layer. For example, Guo et al. used the supervised variational automatic encoder (svae) to learn 6D force and torque (FT). The soft, delicate and reactive grasp realized by tactile intelligence enhanced the underwater interaction of the gripper, greatly reduced the cost, improved the reliability and robustness, and paved the way for intelligent grasp (see Fig. 25b) [290]. Dimensionality reduction proves to be extremely beneficial when it comes to extracting features from data. Bai and his colleagues utilized t-Distributed Stochastic Neighbor Embedding (t-SNE) to project high-dimensional data onto a 2D space, with the aim of visualizing the data while maintaining its global and local structures intact [291]. As depicted in Fig. 25c, different clusters of various datasets collected from twenty types of textiles are presented, which clearly indicates that the data points of the objects can be effectively visualized and clearly distinguished within the 2D space.

Unsupervised learning, as a methodological approach, is frequently utilized within the realm of data analysis or functions as an intermediate stage in the data processing workflow. When endeavoring to unearth the underlying data patterns in the absence of human supervision, the presence of noise typically poses numerous challenges to the algorithm regarding accurate clustering or feature identification, and on occasion, it might even result in contradictory findings. In light of this, it is of crucial importance to establish preprocessing as an essential preliminary step prior to engaging in unsupervised learning. The applications of unsupervised learning are mainly in the fields of classification and dimensionality reduction, while it does not work well for recognition tasks.

#### Supervised Learning

Supervised learning is a machine learning task using labeled data. Given input data (usually as feature vectors) and corresponding output labels (target values), the model aims to learn the input–output mapping to accurately predict output for new, unseen input data. Typical algorithms in tactile sensing are SVM, KNN, decision trees, and LDA. SVM was initially for finding the optimal separating hyperplane in high-dimensional space to classify two types of data points. Initially, it could only handle linear problems but was extended to solve nonlinear ones with the kernel method. SVM can also estimate continuous-valued multivariate functions, handling regression tasks like vibration sensing and force estimation. Lu et al. reported a soft optical robotic hand that could encode tactile stimuli and recognize contact force, position, and hand gestures (Fig. 25d) [38].

KNN computes distances among data samples without extra parameters, based on the assumption that similar points are close. For classification, it first gets all samples, calculates distances between points and their neighbors, and then selects the k nearest neighbors. Depending on voting or averaging, it can be used for classification or regression. With the assistance of the KNN algorithm, the robot is capable of mimicking the user's gestures within milliseconds for the purpose of automatic object manipulation (Fig. 25e) [122]. Similarly, by using the KNN method, Hu et al. fabricated a large-area magnetic skin, achieving the multipoint and multiscale perception. Moreover, Li et al. designed a flexible friction voltage resistance tactile sensor, using biocompatible AF as the flexible substrate, and a scale-like oxide/conductive polymer layer as the sensing material [292]. By further combining it with KNN algorithm, motion detection with 98.9% accuracy and texture surface recognition with 98.3% accuracy are achieved. Constrained by the sharp increase in the amount of data required by the algorithm and the complex manual work, the neural network algorithm has emerged as another highly efficient machine learning algorithm.

The artificial neural network (ANN) algorithm is a remarkable computational approach that intricately mimics the highly sophisticated mechanism of signal transmission within the mammalian nervous system. This complex biological system, which serves as the inspiration for ANN, features a network of neurons that communicate with one another through electrical and chemical signals. The neuron model, often referred to as a unit within the context of ANN, is designed to closely simulate the activation and connection working principles of biological neurons. It attempts to replicate how real neurons in living organisms receive, process, and transmit information. This ANN algorithm is implemented through a variety of mathematical methods, with functions playing a crucial role. These functions are carefully crafted to define the behavior of each neuron and the overall network. For instance, they govern how the inputs to a neuron are combined and transformed to produce an output. When a "neuron" in the ANN receives inputs that reach a specific threshold value, a fascinating process is triggered. It will then be "activated" through an activation function, which is a key component in determining the neuron's response. Once activated, the neuron will send signals to other neurons located in the next layer of the neural network, thereby facilitating the flow of information throughout the network.

Here, we have selected four commonly used models within the realm of artificial neural networks for detailed introduction. These models, namely CNN (convolutional neural network), RNN (recurrent neural network), MLP (multilayer perceptron), and LSTM (long short-term memory), each possess unique characteristics and capabilities that have made them highly popular and effective in a wide range of applications, from image recognition and natural language processing to time-series analysis and many others.

**Fig. 26:**
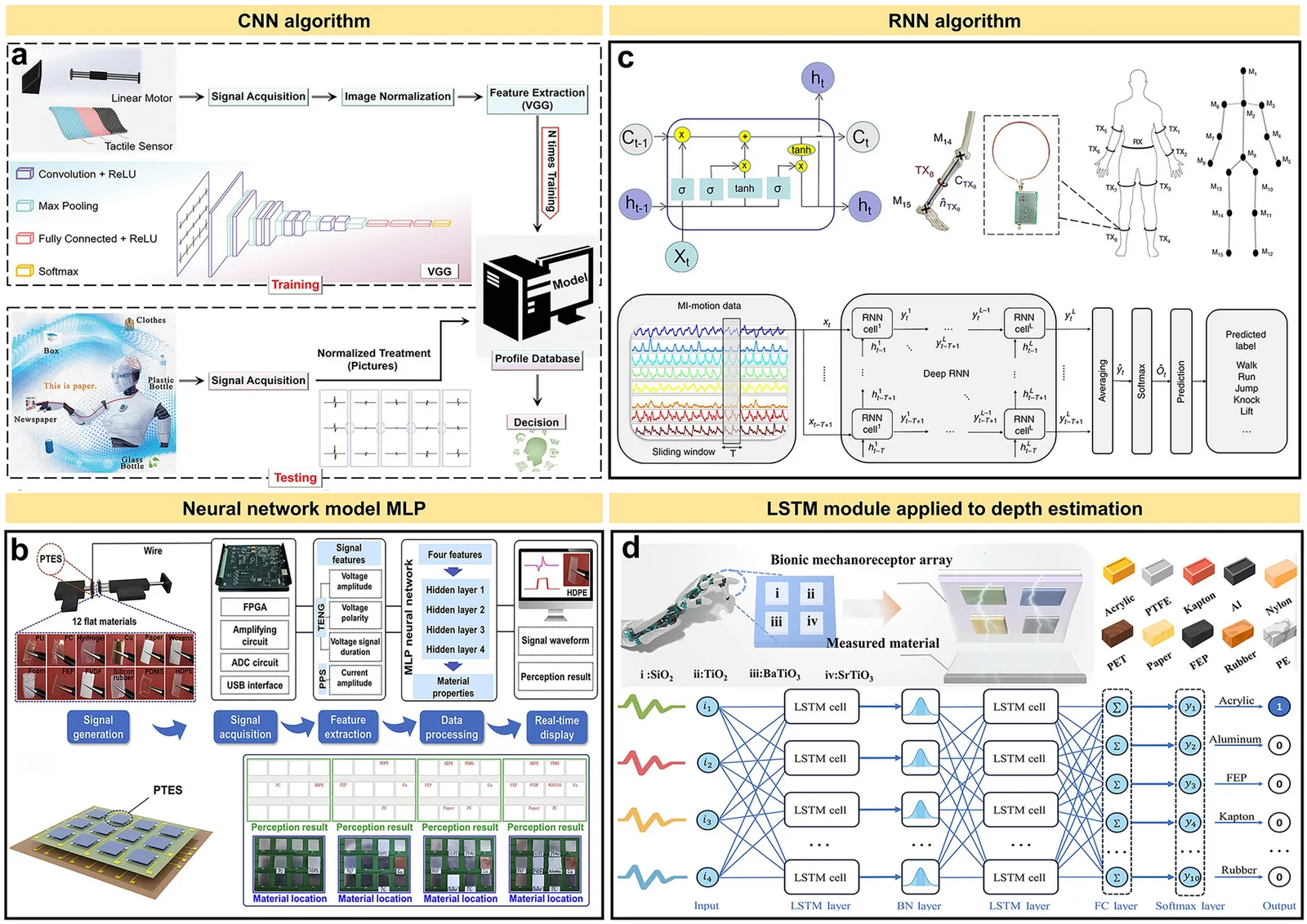
Applications of multiple models in tactile sensing include: **a** the CNN model [293]. Copyright 2022, Wiley‐VCH. **b** RNN model [294]. Copyright 2022, Elsevier. **c** MLP model [295], and **d** LSTM model [296]. Copyright 2020, Springer Nature. Copyright 2024, The American Association for the Advancement of Science

Convolutional neural network (CNN) is a deep learning model that is specifically devised to handle data possessing grid structures like images and audio. With the aid of the CNN model, Wang et al. developed a tactile sensing system for material identification, which enables the extraction of more concealed features of the identified materials and enhances the identification accuracy. The system comprises two processes, namely the training process and the testing/identification process, as depicted in Fig. 26a [293]. Firstly, the model training process is initiated. During this process, a linear motor with predefined pressure and motion procedures comes into contact with different test materials. Subsequently, the induced electrical signals are gathered, and the output voltage signals of nine test materials are examined by the TTS adhered to the front side of the linear motor. The signals from the three channels combine to form a complete image. After undergoing image normalization processing, the image is fed into the VGG model. The VGG model is trained on 80% of the data, and then the accuracy of the model is evaluated on the remaining 20% data points.

A multilayer perceptron (MLP) is constituted by multiple neurons that are arranged in distinct layers, primarily consisting of the input layer, the hidden layer, and the output layer. Specifically, the input layer is tasked with receiving the externally fed data, and the quantity of its neurons typically hinges on the number of features within the input data. The hidden layer, positioned between the input layer and the output layer, undertakes the processing and transformation of the input data. Subsequently, the output layer generates the ultimate output based on the outcomes processed by the hidden layer, with the output of each neuron denoting the probability of the corresponding category. Xiao et al. accomplished the real-time identification of material properties upon touching objects with smooth surfaces by integrating a sensing device with an intelligent material sensing system aided by the MLP neural network. This demonstrated an exceptional ability that outperforms human skin perception. The established intelligent material sensing system, depicted in Fig. 26b, comprises signal generation and collection, feature extraction, data processing and analysis, along with the real-time display of results [294].

Recurrent neural network (abbreviated as RNN) is a kind of neural network that is widely utilized in the realm of deep learning. It is particularly apt for handling sequential data and has the ability to capture the long-term dependencies within the sequence, namely the correlations between data points that are spaced far apart in the sequence. Long short-term memory (LSTM), which is a typical and commonly seen model of RNN, can remember the last output, cycle, update, and adapt by virtue of its architecture. An LSTM unit is composed of an input gate, an output gate, and a forget gate. In the study conducted by Golestani and Moghaddam, LSTM was implemented with the aim of recognizing human activities. The system is founded on the magnetic induction signals generated by the spatially distributed sensors on the human body (as depicted in Fig. 26c) [295]. The voltage gain of the system and the magnetic induction signals exhibit a strong relationship with the geographical translation of the body segments.

The LSTM is fully capable of resolving the problem of long-term dependencies. Wang et al. employed the LSTM neural network as the classifier for each layer within the cascaded classifier, as depicted in Fig. 26d [296]. Applied to process data collected by the multimodal sensor array of a bionic robotic hand, this model was trained using supervised learning to identify unknown materials. The pre-trained model achieved an overall accuracy of 99.56%, as indicated by its confusion matrix. To prevent overfitting, dropout layers were introduced during fine-tuning. The results demonstrate that the model can attain an accuracy of 99.72% in tactile perception training.

Different machine learning algorithms exhibit distinct trade-offs in classification accuracy, real-time performance, training data requirements, and hardware compatibility, making algorithm selection a critical consideration. The comparative advantages, key deployment challenges, and suitable application scenarios of various algorithm types are summarized in Table 4.

**Table 4:** Comparisons of several machine learning algorithms for signal

Algorithm	Advantages	Disadvantages	Difficulties	Applications
K-means	Intuitive clustering results	Sensitive to noise and outliers	Dynamic tactile model failure	Materials classification
PCA	High dimensionality reduction efficiency	Loss of physical spatial information	Unable to decouple mixed forces	Motion artifact removal
Dictionary Learning	Strong noise resistance	Slow convergence during training	Unstable training	Dynamic tactile feature encoding
Autoencoder	Strong non-linear feature extraction capability	High demand for data	A large amount of abnormal data requirement	Tactile representation learning
SVM	Strong robustness of high dimensional features	Slow computation of large-scale arrays	Sensitive to tactile signal drift	Static tactile recognition
KNN	Simple implementation	High sensitivity to noise	Difficulty in processing high-dimensional signals	Real time contact detection
ANN	Support multimodal input fusion	Complex parameter adjustment	Lack of dynamic features in deep networks	Multi-sensor fusion
CNN	Strong spatial feature extraction ability	Large scale annotation of data	Long range force transmission cannot be modeled	Pressure distribution, shape recognition
MLP	Flexible and easy to expand structure	Loss of spatial topology information	Data dimensionality reduction destroys local correlations	Single point signal classification
LSTM	Strong long-term capture capability	Poor real-time performance	Calculation delay	Gesture action tracking

## Multi-dimensional Challenges and Strategies

### Performance-Level Challenges and Improvement Strategies

#### Delayed Rapid Responsiveness

**Fig. 27:**
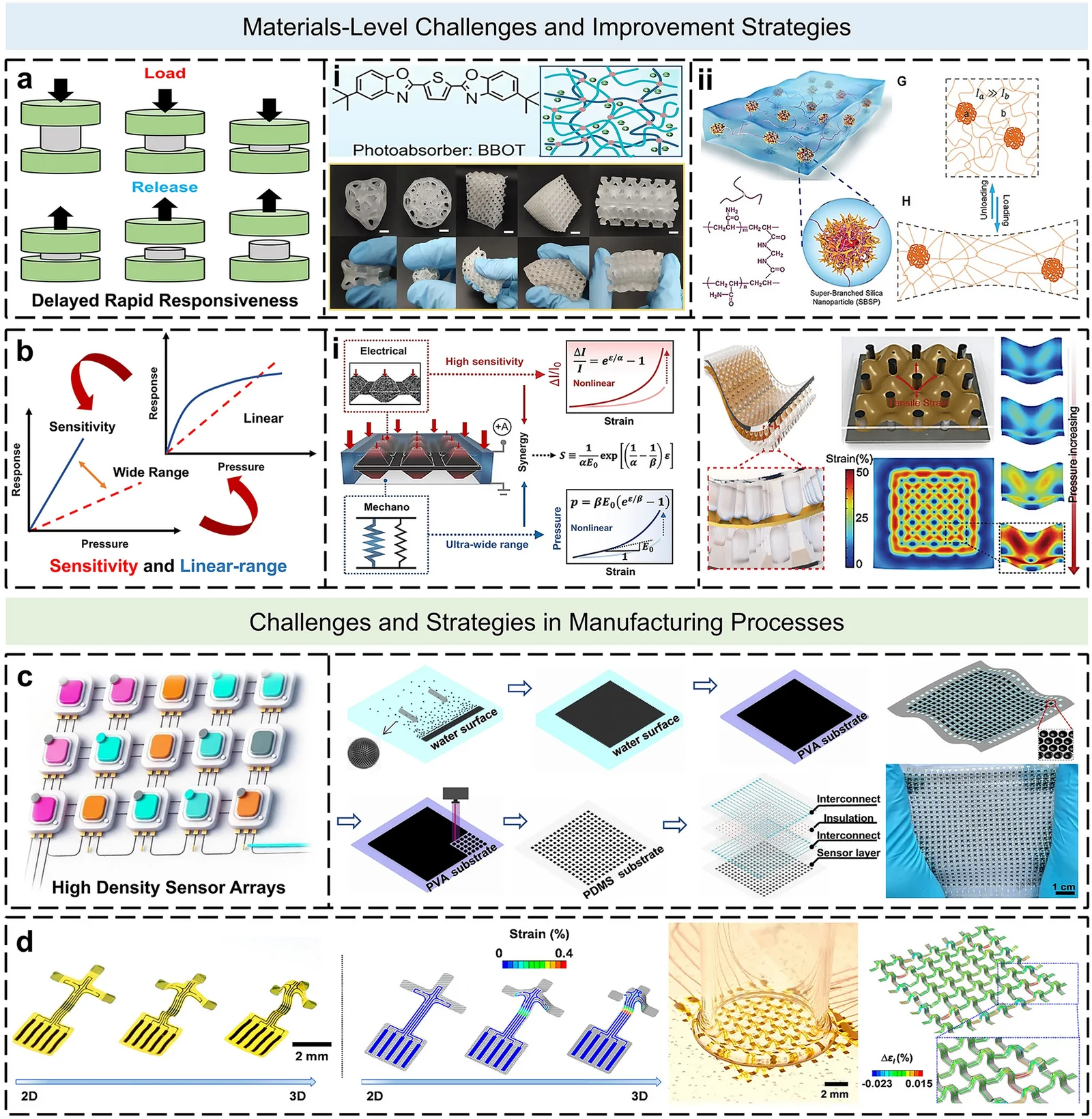
**a** Enhance the hysteresis behavior of materials by leveraging the macroscopic structure and the inherent rebound properties of materials, specifically: (i) Elastomers with microlattice structures [297], (ii) hysteresis-free hydrogels reinforced by nanoparticles [298]. Copyright 2022, Elsevier. Copyright 2022, Wiley–VCH. **b** Tunable structural stiffness: (i) the stiffness regulator (SR)—around the sensing layer [299], (ii) Stiffness-graded sensor layer design [300]. **c** Laser ablation process for fabricating high-density, uniform array structures [301]. **d** 3D cage-like sensor array [302]

The influences derived from viscoelasticity and chain relaxation of elastomeric materials are significant in the output linearity, sensitivity, and response time of sensors, as illustrated in Fig. 27a. To conquer this issue, researchers have conducted extensive explorations in regulating the macro- and microscopic structures of the elastomeric materials. Peng et al. prepared a photocurable resin containing ionic liquids (ILs) and hydrogen-bond-rich acrylate monomers (Fig. 27a(i)) [297]. Combined with an optimized lattice structure, the porous ionogel flexible sensor (PIFS) exhibits higher pressure sensitivity and lower hysteresis (2.4%), and can provide reliable signals during cyclic loading (~ 500 cycles). In consideration of enhancing the intrinsic properties of materials, Russel and his colleagues made use of a low cross-linking density polyacrylamide hydrogel with a water content of 96% and adopted hyperbranched silica nanoparticles (HBSP) as the primary connection points, thereby achieving a hysteresis-free material (Fig. 27a(ii)) [298]. The non-fatigue characteristic of the composite hydrogel is demonstrated by the invariance of the stress–strain curve when the strain ratio is 4, even after going through 5,000 cycles. When the strain ratio is 7, only 1.3% of hysteresis was noticed.

#### Conflict Between Sensitivity and Detect Range

The fundamental contradiction between the sensitivity and detection range of tactile sensors stems from the fact that enhancing sensitivity typically requires reducing structural stiffness (e.g., through flexible microstructures), which inevitably compromises the measurement range. Conversely, expanding the detection range necessitates increasing stiffness, thereby diminishing the responsiveness to weak signals. To achieve synergistic optimization of both performance metrics, Zhou et al. proposed a novel nonlinear cooperative design strategy for flexible pressure sensors by introducing an elastic spacer—termed a stiffness regulator (SR)—around the sensing layer to modulate load distribution [299]. The structure is shown in Fig. 27b(i). This approach achieved remarkable linearity with a sensitivity of 24.6 kPa^−1^ and a broad detection range spanning from 0 to 1.4 MPa. Similarly leveraging differentiated design in stiffness and modulus of the sensor layer materials, Huang et al. proposed a strain-effect strategy [300]. This approach utilizes the interlocking motion of staggered microdomes to generate tensile strain, thereby producing a positive resistance response, as shown in Fig. 27b(ii). The designed architecture simultaneously achieves both an extended detection range (45 Pa–4.1 MPa) and high sensitivity (70 kPa^−1^), demonstrating exceptional performance in flexible pressure sensing applications.

### Challenges and Strategies in Sensor Manufacturing Processes

#### Fabrication Challenges of High-density Arrays

Manufacturing and integration challenges of high-density sensor arrays are critical constraints in tactile sensing systems. The primary difficulties lie in alignment precision at the micro/nanoscale and material compatibility. The combination of transfer printing and patterning techniques offers an effective solution to these issues. Transfer printing enables batch transfer of sensor units, while patterning technology provides unique advantages in precise structural formation and uniformity control. Wu et al. demonstrated a laser ablation process for fabricating strain-sensing thin-film arrays, which were subsequently transferred onto PDMS substrates to ensure stretchability [301]. Electrical interconnections were established through precision dispensing. As shown in Fig. 27c, this approach achieved an array density of 100 pixels cm⁻^2^ while maintaining high uniformity (standard deviation < 3.81%). 3D-curved structures offer a novel approach for high-density, multifunctional sensor arrays by enabling vertical stacking (e.g., wavy or helical configurations) that surpasses planar limitations. Sang et al. demonstrated a buckling-assisted transformation of 2D precursors into 3D cage-like architectures, significantly improving spatial efficiency while endowing the curved microstructures with multi-directional force-sensing capabilities [302]. The manufacturing procedure is schematically depicted in Fig. 27d.

### Challenges and Strategies in Structure and Signal Transmission

#### Interface Modulus Mismatch

**Fig. 28:**
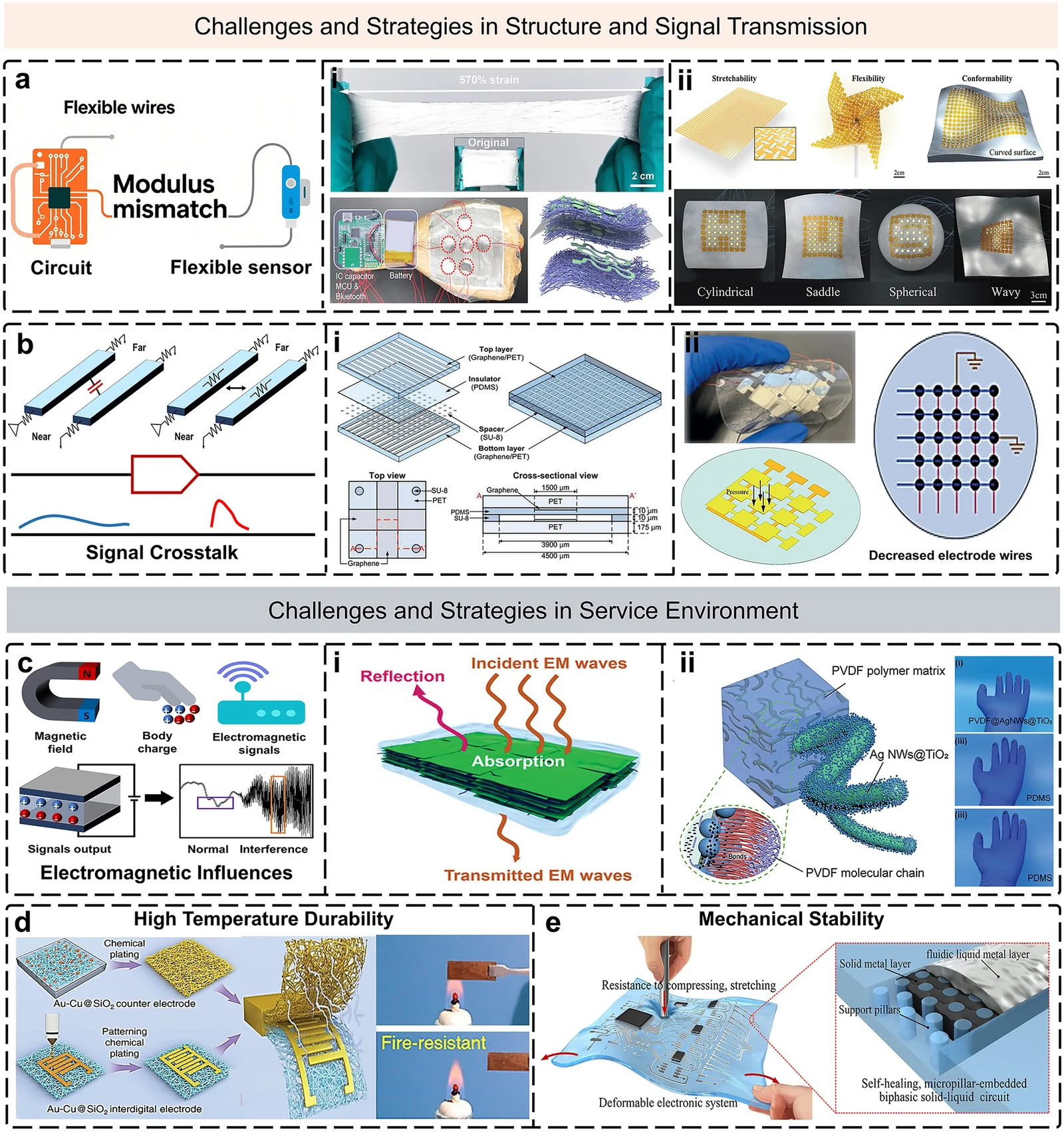
**a** Modulus mismatch at the sensor interface: (i) highly robust stretchable electrodes based on nanoliquid metal [303], (ii) Snake-skin-inspired soft-hinge Kirigami metamaterials [304]. Copyright 2022, The Authors. Copyright 2022, Wiley‐VCH. **b** Solutions to the signal crosstalk problem: (i) Capacitive tactile sensors with graphene electrodes facing each other and separated by two sets of spacers [81], (ii) simplified wiring design and crosstalk-resistant insulation layer design [305]. Copyright 2017, WILEY–VCH. Copyright 2021, The Authors. **c** Electromagnetic shielding materials: (i) Modified graphene nanosheets/cellulose nanofiber materials [306], (ii) core–shell structured AgNWs@TiO_2_ film materials [307]. Copyright 2024, Wiley‐VCH. Copyright 2023, Wiley‐VCH. **d** Flexible high-temperature-resistant ceramic electrode [308]. **e** Impact-resistant solid–liquid self-healing circuit design [309]

The sensing elements of sensors are usually connected to flexible wires, which inevitably leads to the problem of modulus mismatch at the interface. The phenomenon of interface modulus mismatch will trigger a sharp decrease in a large number of functional parameters of the device. Meanwhile, it tends to damage the device interface and undermine the durability of the device. Conformal electrodes can closely adhere to the surfaces of irregular objects. This significantly suppresses the interlayer slipping phenomenon that exists between the electrodes and the functional layers in sensor devices while providing the maximum contact area. Therefore, the research on conformal electrodes has become an important solution to this problem. Li et al. demonstrated a highly robust stretchable electrode (NHSE) based on nanoliquid metal (nLM), as shown in Fig. 28a(i) [303]. There is only a 350% change in resistance under an elongation rate of 570%. This excellent characteristic is attributed to the adaptive interface between the LM and the nanofiber scaffold, which mimics the interaction between water and a net. Meanwhile, it exhibits excellent robustness to dynamic cyclic stretching and environmental stimuli (i.e., heating, exposure to acids and bases, and immersion). Inspired by the skin of snakes, Huang and his colleagues put forward a novel type of electronic armor (E-armor), the structure is shown in Fig. 28a(ii) [304]. This E-armor not only has the mechanical flexibility and electronic functions that are akin to those of E-skin, but also is capable of safeguarding itself as well as the underlying soft components from external physical harm. It also exhibits the characteristics of stretchability, conformability, and protectability, which are highly applicable to flexible electronic products.

#### Crosstalk Suppression

Crosstalk is defined as the interference phenomenon in which the signal within one signal channel perturbs the signals in other adjacent or related signal channels across numerous fields such as electronic systems, communication lines, and signal transmissions. This phenomenon is predominantly attributed to factors like electromagnetic coupling, along with signal reflection and impedance mismatch. It can exert several detrimental effects on the system, encompassing signal distortion, a rise in the bit error rate, and a degradation of performance. To preclude the crosstalk problem from impinging on the performance of devices, researchers frequently optimize the system by way of enhancing the layout and routing of lines and implementing shielding techniques, among other approaches.

In order to avert crosstalk occurring between adjacent units within the device array, Pyo and his colleagues incorporated stiffer mechanical spacers between the sensing units, as shown in Fig. 28b(i) [81]. The sensors were composed of capacitors fabricated from a PDMS/air dielectric positioned between graphene electrodes along with SU-8 spacers. When pressure was exerted, the dielectric layer of the sensing unit would undergo deformation, thereby augmenting the capacitance between the two electrodes. Meanwhile, the confining effect of the spacers serves to preclude the deformation of non-target units.

Additionally, the development of larger-area and higher-resolution electronic devices is constrained by the substantial number of electrode wires required. While the row-plus-column wiring strategy simplifies device routing to some extent, an n × m sensing array still necessitates n × m wires, resulting in high routing complexity. Hong et al. developed a simple yet effective electrode topology that eliminates crosstalk [305]. This topology required only n + m wires for an n × m sensor array. Furthermore, by incorporating an insulating layer between sensing layers, crosstalk interference was further suppressed, as illustrated in Fig. 28b(ii).

### Challenges and Strategies in Service Environment

#### Electromagnetic Influences

The accumulation of heat within electronic components and the presence of electromagnetic interference can exert adverse impacts on the sensing performance (Fig. 28c). Yang et al. fabricated cellulose nanofiber (CNF)-based composites, which boasted high thermal conductivity (TC) and excellent electromagnetic interference (EMI) shielding properties, by utilizing CNF as a template, as shown in Fig. 28c(i) [306]. The resultant CNFs-based composites featured a high TC of 136.2 W m^−1^ K^−1^) and an outstanding EMI shielding effectiveness of 105 dB. In the context of capacitive tactile sensors, it was rather prevalent to encounter the situation where signal instability arisen due to electromagnetic interference and the proximity effect. In light of this, Pan et al. demonstrated a highly anti-interference capacitive flexible pressure sensor, which employed polyvinylidene fluoride (PVDF)@AgNWs@TiO_2_ film as the dielectric layer, as shown in Fig. 28c(ii) [307]. The core–shell structure incorporated within this sensor served a dual purpose. On one hand, it facilitated the augmentation of the initial capacitance. On the other hand, it contributed to the equilibration of the dielectric constant, dielectric loss, and breakdown strength within the dielectric layer. The sensor fabricated based on this particular material displayed a remarkably high signal-to-noise ratio when confronted with diverse interference sources.

#### Operational Limitations in Extreme Environments

The development of tactile sensors capable of reliable operation in extreme environments (e.g., extreme high/low temperature, mechanical shock, high frequency, etc.) remains an underexplored yet critical research frontier. Conventional flexible materials (e.g., PDMS) exhibit softening above ~ ‒50 ℃, while metal electrodes suffer from oxidation, limiting their applicability. Structurally, high-pressure or impact conditions often damage interconnects, degrading performance. Flexible conductive ceramic electrodes represent a promising solution for high-temperature tactile sensors. Yan et al. developed a Cu/Au nanolayer-deposited toughened SiO₂ nanofiber network, where in situ thermal reactions formed conformal Au₃Cu/CuSiO₃ interphases, enabling fire-resistant sensing (Fig. 28d) [308]. For impact resistance, Chen et al. designed a liquid–solid biphasic self-healing circuit, as shown in Fig. 28e. A lower solid metal layer provided compressive strength, while an upper liquid metal layer autonomously filled cracks under deformation, achieving instantaneous conductivity recovery during stretching/impact [309].

**Fig. 29:**
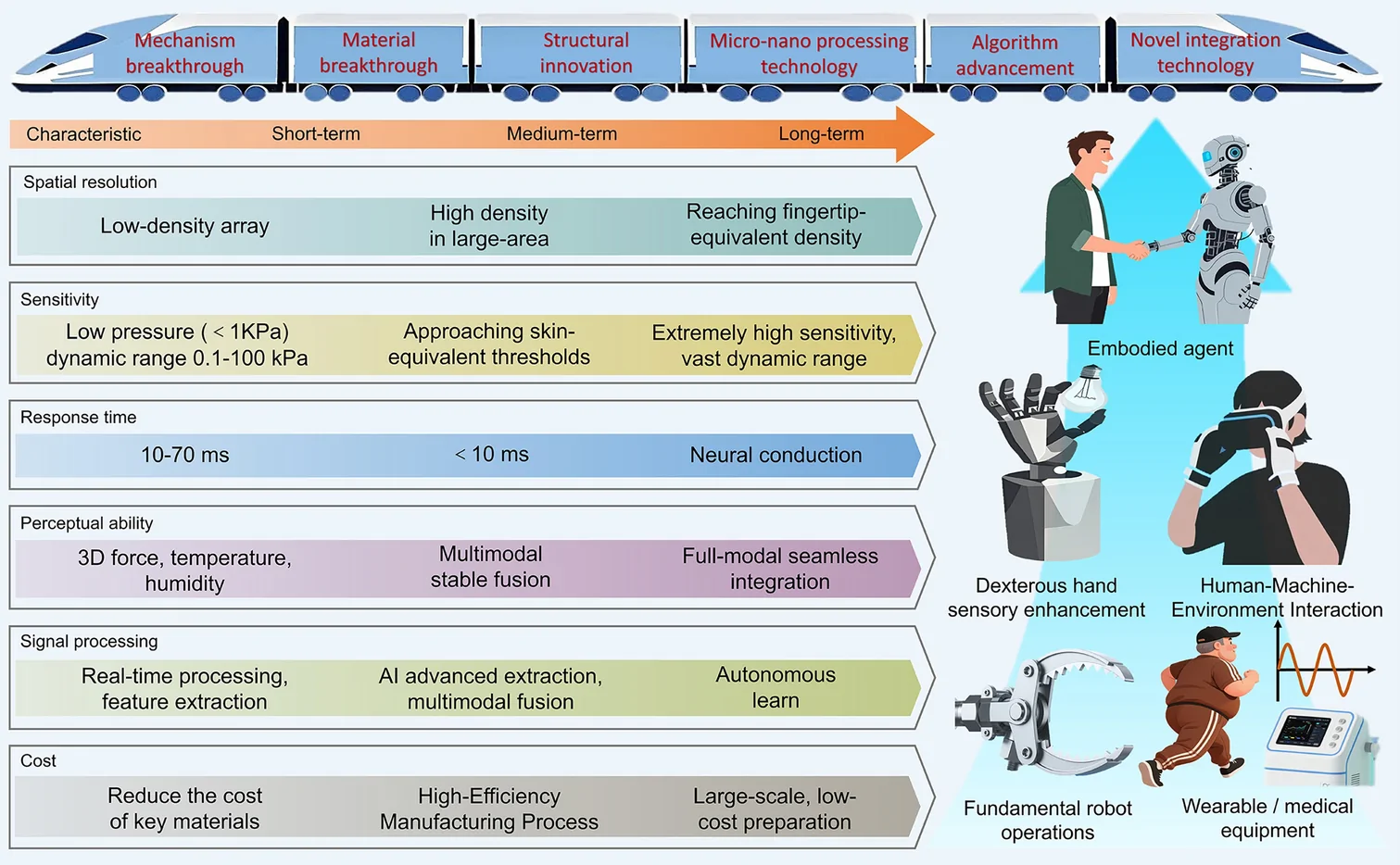
Forward-looking development roadmap and application perspectives of tactile sensors

Additionally, corrosion-resistant environments can be addressed through self-protective design strategies to isolate sensors from corrosive media. Meanwhile, complex operational environments have inspired innovative power supply architectures for sensors. For instance, thermoelectric energy harvesting or piezoelectric energy generation in high-pressure aqueous environments could enable novel application scenarios. However, more complicated service environments emerge continuously in the vast applications of flexible tactile sensors, which requires further more considerations in sophistically designing the integrity of structure and function (Fig. 29).

## Summary and Prospects

With the emerging of flexible electronics industry, remarkable progress has been achieved in the research of flexible tactile sensors, which is mainly manifested in aspects such as new materials, novel structures, as well as advanced manufacturing and integration processes. Among these, the innovation of functional materials and structures have notably enhanced the technical performance of tactile sensors, encompassing various crucial performance parameters mentioned previously. Meanwhile, advanced integration and manufacturing processes have further boosted the precision and integration level of sensors, elevated production efficiency, and are beneficial for promoting the extensive application of tactile sensors. In the realm of robotics, flexible tactile sensors are set to become the pivotal components that enable robots to attain intelligence and autonomy. Future robots will be outfitted with a substantial number of flexible tactile sensors, allowing them to perceive the surrounding environment with greater accuracy and execute more precise operations and more flexible movements. For instance, in industrial robots, flexible tactile sensors can be utilized to detect the shape, size, and surface quality of workpieces, thereby enhancing production efficiency and product quality; in service robots, these sensors can assist robots in better understanding human needs and providing more considerate services. To further accomplish the efficient development from device fabrication to robotic tactile perception, we hold the belief that more in-depth research should be conducted from the following themes:

1. Inherent performance of sensors.

The inherent sensing performance of the sensor calls for further enhancement, which encompasses several aspects: Firstly, the development of advanced packaging techniques is necessary to prevent damage during long-term operation. Secondly, the tensile capabilities of the sensor can be boosted through strategies like molecular engineering and supramolecular assembly. Thirdly, an optimized device structure can be attained by combining multiple materials. Lastly, the research and development of novel materials with new sensing mechanisms should also be carried out.

(2) New design concept with algorithms.

Traditionally, the design of tactile sensing systems has typically centered on the structure and performance of sensors. However, when machine learning tools are employed for assistance, the optimal performance that machine learning can offer is undoubtedly compromised. Developing machine learning algorithms to guide the design of sensors proves to be an effective solution to this issue. By taking into account the selection and design of algorithms for signal processing procedures, the desired performance is initially defined. The target performance, functioning as an iterative structure, provides effective guidance for the design of sensors, thereby achieving an efficient and streamlined design process of the sensing system.

(3) Advanced integration strategies.

The integration strategies for tactile sensor devices have been extensively investigated, encompassing 3D lamination technology, residual stress-induced assembly, interface delamination-induced assembly, and capillary force-induced assembly, among others. Nevertheless, the structures of the fabricated devices are prone to being rather uniform, rendering it challenging to manufacture microelectronic devices with intricate 3D architectures. Following the introduction of several mechanical traction 3D structure design strategies that progress from 2 to 3D, the functionalization within the three-dimensional realm has emerged as a prominent research focus regarding the structure of tactile sensors in the future. The development of advanced 3D electronic device processing technologies merits further exploration.

(4) Integrated sensing and driving.

Developing an intelligent system integrating tactile sensing signal input, processing, and feedback is worthy of attention. Achieving this goal surely poses more challenges to front-end sensing and integration technologies, as well as back-end data processing and signal feedback. Currently, most machine learning methods in tactile sensing come from algorithms in vision, natural language processing, etc. They either use algorithms to process tactile signals or convert them into ideal inputs for algorithms. This inevitably causes information redundancy or loss. Tactile signal algorithms specifically designed for robotic tactile systems still require further research.

(5) Standardized Performance Evaluation Methodology

The establishment of standardized performance evaluation frameworks serves as a critical bridge transitioning tactile sensors from laboratory research to industrial applications. Its significance transcends mere technical considerations, driving a qualitative leap from fragmented innovations to systematic breakthroughs in the field.
